# FM-GPT: Bayesian fine mapping for phenome-wide transcriptome-wide association studies

**DOI:** 10.1371/journal.pgen.1012126

**Published:** 2026-09-08

**Authors:** Travis Canida, Zhenyao Ye, Shao-Hsuan Wang, Hsin-Hsiung Huang, Yezhi Pan, Menglu Liang, Shuo Chen, Tianzhou Ma

**Affiliations:** 1 Department of Epidemiology and Biostatistics, School of Public Health, University of Maryland, College Park, Maryland, United States of America; 2 Department of Mathematics, University of Maryland, College Park, Maryland, United States of America; 3 Maryland Psychiatric Research Center, Department of Psychiatry, School of Medicine, University of Maryland, Baltimore, Maryland, United States of America; 4 Department of Epidemiology and Public Health, School of Medicine, University of Maryland, Baltimore, Maryland, United States of America; 5 Graduate Institute of Statistics, National Central University, Taoyuan City, Taiwan; 6 Department of Statistics and Data Science, University of Central Florida, Orlando, Florida, United States of America; The University of Chicago, UNITED STATES OF AMERICA

## Abstract

Transcriptome-wide association studies (TWAS) integrate genome wide association studies with expression quantitative trait locus reference panels to identify genes associated with traits of interest. However, linkage disequilibrium and correlated gene expression can induce spurious TWAS signals, motivating fine mapping methods to prioritize putatively causal genes within associated loci. The rapid growth of large-scale phenomic resources (e.g., electronic health records (EHRs)) has shifted genetic studies from single-trait analyses to phenome-wide investigations that jointly evaluate many closely related phenotypes. We introduce FM-GPT (**F**ine-**m**apping of causal **G**enes for **P**henome-wide **T**ranscriptome-wide association studies), a novel Bayesian fine mapping method for prioritizing causal genes across multiple correlated phenotypes with potentially mixed outcome types (e.g., continuous, binary, multinomial or count) in phenome-wide TWAS. FM-GPT performs gene-guided dimension reduction of the phenotypes and reveals pleiotropic or phenotype-specific effects of the identified genes. In simulations, FM-GPT identified true causal genes more accurately than other fine mapping methods while controlling false positives. We applied FM-GPT to two applications using data from UK Biobank: a brain-wide genetic analysis of MRI data derived regional cortical thickness measures and a phenome-wide genetic analysis of clinical phenotypes derived from EHR data. FM-GPT greatly narrowed down the set size of putatively causal genes and identified: 1. genes with pleiotropic effects on regional cortical thickness across the cerebral cortex, including five genes *BCAS3*, *LRRC37A*, *NOS2P3, ARL17B* and *UBB* on chromosome 17 regulating neuronal morphology and cortical organization; and 2. genes that influence multiple medical conditions across the circulatory, metabolic, digestive, respiratory and genitourinary systems, revealing two major axes of variation among these conditions that point to a potential trade-off in gene regulation between immune and metabolic functions. These results highlight FM-GPT’s power to disentangle complex gene–phenotype relationships in large-scale phenome-wide studies, revealing biological mechanisms underlying diverse human traits and advancing translational and comorbidity research.

## Author summary

We developed a novel fine mapping method called FM-GPT, to identify putatively causal genes from correlated noise influencing a wide range of human traits and diseases with potentially mixed outcome types (e.g., binary, count or continuous). The rapid expansion of large-scale phenomic datasets has shifted the single-trait genetic studies to phenome-wide analyses, enabling the study of genetic architecture across many related traits simultaneously. FM-GPT performs gene-guided dimension reduction of the phenotypes and reveals pleiotropic or phenotype-specific effects of the identified causal genes. When applied to the UK Biobank data, FM-GPT greatly narrowed down the set size of putatively causal genes compared to other methods. The tool identified genes with pleiotropic effects on regional cortical thickness that regulate neuronal morphology and cortical organization across the cerebral cortex. It also identified genes that influence multiple medical conditions spanning the circulatory, metabolic, digestive, respiratory, and genitourinary systems. Among these conditions, two major axes of variation emerged, revealing a potential trade-off in gene regulation between immune and metabolic functions. This work provides a clearer picture of biological mechanisms across traits and diseases, advancing translational research and the understanding of comorbidity.

## Introduction

Transcriptome Wide Association Studies (TWAS) integrate Genome Wide Association Studies (GWAS) with expression quantitative trait locus reference panels (e.g. GTEx [1]) to identify genes whose genetically regulated expressions (GReX) are associated with a trait of interest [2,3]. TWAS typically proceed in two steps: First, gene expression prediction models are trained using reference panels with matched genotype and gene expression data, and these models are then used to impute GReX in GWAS. Second, association analyses are performed between GReX and the trait of interest to identify candidate susceptibility genes. Since the introduction of the first TWAS method PrediXcan [4], many TWAS methods have been developed that employ different statistical learning methods for expression prediction [5–7], integrate information across multiple tissues [8–10], or leverage GWAS summary statistics [8,11,12]. Despite these advances, interpreting TWAS associations remains challenging: TWAS methods test genes individually without accounting for the correlation among predicted gene expressions arising from linkage disequilibrium (LD) among variants or shared regulatory architecture [2,3,13]. To address this issue, several TWAS fine mapping methods [14–16] have been developed to prioritize putatively causal gene within associated regions. However, existing TWAS fine-mapping methods are primarily designed for single-trait analyses, limiting their applicability in settings where multiple related phenotypes are analyzed jointly.

Meanwhile, the rapid expansion of large-scale phenomic datasets has shifted the focus of human genetics from single-trait studies to phenome-wide analyses. Resources such as the UK Biobank (UKB) provide extensive collections of phenotypes derived from electronic health records (EHR), imaging-derived phenotypes (IDPs) and other clinical or non-clinical measurements. Phenome-wide TWAS analyses offer an opportunity to systematically investigate how genes regulate a broad spectrum of phenotypic outcomes. However, these analyses also introduce new methodological challenges. First, phenotypes in large-scale phenomic datasets are often highly correlated, and analyzing them independently can substantially increase the multiple-testing burden while reducing statistical power. Secondly, phenome-wide datasets frequently contain mixed outcome types, including continuous, categorical, and count outcomes, which complicates joint modeling [17–19]. Thirdly, gene-phenotype relationships are inherently complex: individual genes may influence multiple phenotypes (pleiotropy), while most phenotypes are regulated by multiple genes (polygenicity). Disentangling these many-to-many relationships across both the transcriptome and the phenome remains an important and largely unresolved challenge.

To fill the gap, we propose FM-GPT (Fine-Mapping of causal Genes for Phenome-wide Transcriptome-wide association studies), a novel Bayesian fine mapping method for identifying putatively causal genes across a number of related phenotypes in phenome-wide TWAS. FM-GPT jointly prioritizes putatively causal genes for multiple correlated phenotypes while accommodating outcomes of mixed data types (e.g., continuous, binary or count). The method leverages GReX information to guide dimension reduction of the phenotypes, prioritizes putatively causal genes associated with the latent phenotype factors, and reveals pleiotropic or phenotype-specific effects of the identified genes. FM-GPT enables computationally scalable analysis of large phenomic datasets while facilitating the interpretation of complex gene-phenotype relationships and possibly heterogeneous pleiotropic effects.

Through extensive simulations, we demonstrate that FM-GPT improves the identification of true causal genes relative to existing TWAS fine-mapping approaches while controlling false positives. We further applied FM-GPT to two real data examples in the UK Biobank (UKB). In a brain-wide genetic analysis of MRI data derived regional cortical thickness measures, FM-GPT greatly narrowed down the set size of putatively causal genes compared to other fine mapping methods and identified a set of putatively causal genes with pleiotropic effects on regional cortical thickness across the cerebral cortex, including five genes *BCAS3*, *LRRC37A*, *NOS2P3, ARL17B* and *UBB* on chromosome 17 regulating neuronal morphology and cortical organization. In a phenome-wide genetic analysis of EHR data derived clinical phenotypes, we identified genes that influence multiple medical conditions across the circulatory, metabolic, digestive, respiratory and genitourinary systems, and revealed two major axes of variation among these conditions pointing to a potential trade-off in gene regulation between immune and metabolic functions. These results highlight the utility of FM-GPT for disentangling complex gene-phenotype relationships in large-scale phenome-wide genetic studies, enabling the identification of biological mechanisms across related traits and diseases and strengthening the foundation for translational and comorbidity research.

## Results

### Overview of FM-GPT method

**Fig 1:**
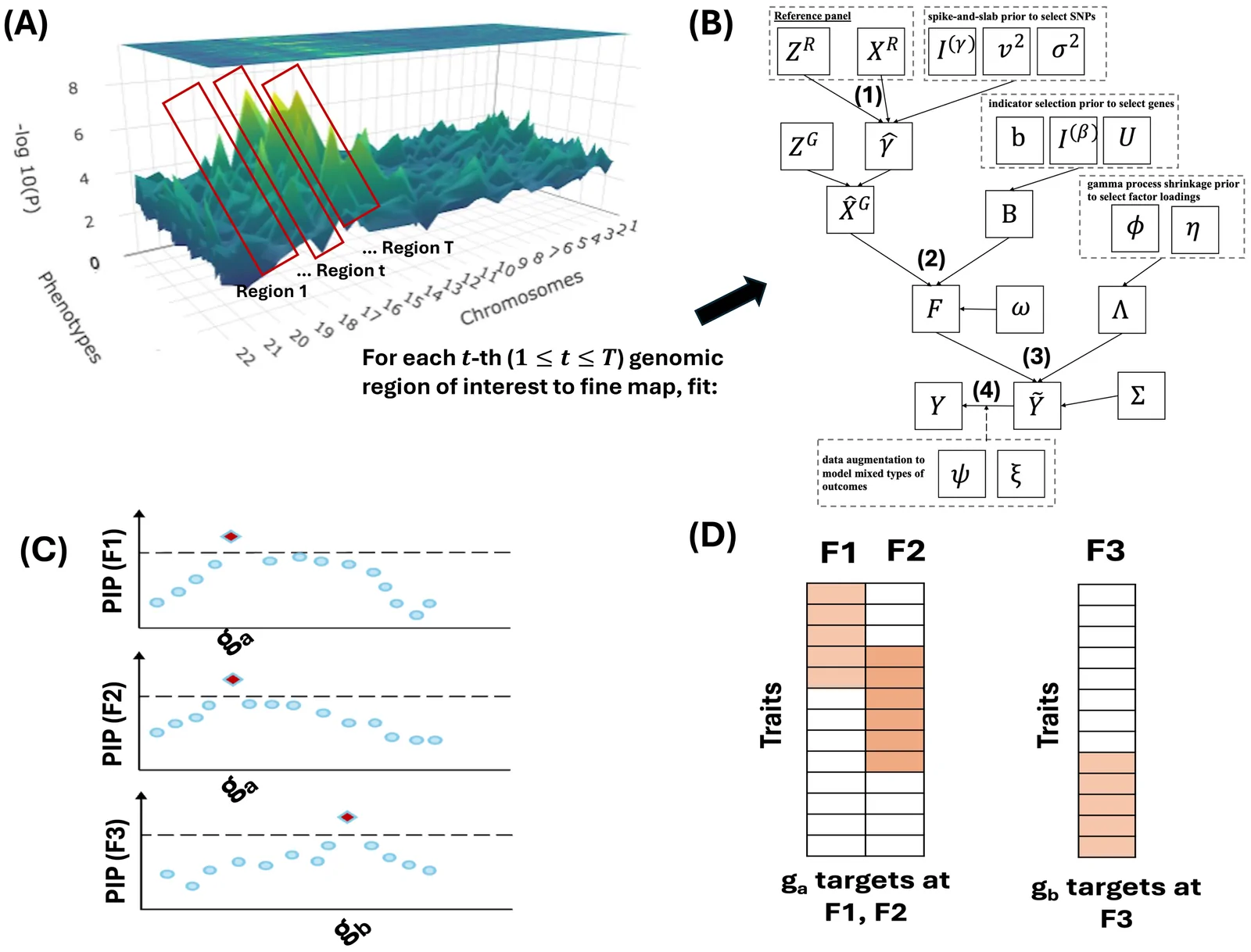
A schematic overview of the FM-GPT method. (A) Demonstration of phenome-wide TWAS results, we will apply fine mapping to each of those highlighted genomic regions. (B) A directed acyclic graph (DAG) showing the main prior settings that characterize the FM-GPT method. $Z^{R}$ and $X^{R}$ correspond to the genotype and gene expression data from reference panel; $Z^{G}$and ${\hat{X}}^{G}$ are the genotype and estimated GReX data from GWAS; F is the latent phenotypic factors and Y is the multiple phenotypes potentially of mixed data types; (1)-(4) correspond to Equation (1)–(4) in the Methods section. (C-D) Main outputs from the FM-GPT method: the putatively causal genes for the phenotype factors (C); the loadings of phenotypes on each phenotype factor (D).

Fig 1 shows a schematic overview of our FM-GPT method. Like most fine mapping methods, FM-GPT started by identifying the genomic regions of interest to fine map. For a single phenotype, regions of interest are typically selected from univariate TWAS results based on genome-wide signals. When multiple phenotypes are jointly analyzed, we first obtain the univariate TWAS results over the whole genome for each phenotype and then apply standard meta-analysis approaches, such as Fisher’s method for combining p-values across traits, to identify genomic regions showing evidence of association (e.g., highlighted part in the 3D Manhattan plot in Fig 1A). Fisher’s method is used here solely as an initial screening procedure to prioritize genomic regions for subsequent fine-mapping analysis, rather than as the final inferential step for gene discovery nor provide formal significance testing or control false positive rates. Alternative p-value combination methods that are robust to correlated tests, such as the Cauchy combination test [20], could also be employed in this screening stage. FM-GPT performs fine mapping in one genomic region at a time, where the genomic regions are defined based on LD structure (e.g., LD blocks defined by LDetect [21]).

Fig 1B shows the directed acyclic graph (DAG) of the full Bayesian model (see Methods for details). FM-GPT first trains the genotype-gene prediction models in a reference panel ($Z^{R}$ and $X^{R}$) to estimate genotype-gene weight matrix (γ), which is then used to impute GReX (${\hat{X}}^{G}$) in the GWAS cohort (see Equation (1) in Methods). Standard spike-and-slab prior (Methods section 2) are imposed to select SNPs for each gene in γ. The imputed ${\hat{X}}^{G}$ is subsequently used to assess association with the phenotypes of interest. **(2)** To jointly analyze multiple correlated phenotypes, FM-GPT integrates a Bayesian infinite factor analysis model within a Bayesian variable selection framework. Unlike conventional factor analysis that assumes a single global factor structure shared across all genomic regions, FM-GPT analyzes each genomic region separately (Fig 1A and 1B). The latent phenotypic factors are modeled as linear combinations of GReX within a genomic region (see Equation (2) in Methods). This formulation enables supervised dimension reduction of the phenotype space while allowing gene expression signals to guide the identification of latent phenotype factors. Within each genomic region, the GReX of all candidate genes is modeled simultaneously to prioritize putatively causal genes associated with these latent phenotype factors, i.e., to estimate B. Indicator selection priors (Methods section 2) are imposed to select genes associated with each factor in B. Gamma process shrinkage priors (Methods section 2) are used to impose shrunken factor loadings and identify the subset of phenotypes most strongly influenced by the genes associated with each latent factor for revealing phenotype-specific gene regulation and improving the interpretability of the inferred factors (see Equation (3) in Methods). Lastly, FM-GPT accommodates phenotypes of mixed data types - including continuous, binary, multinomial and count outcomes - through a data augmentation strategy embedded within the full Bayesian model (see section 3 and Equation (4) in Methods). The major outputs of FM-GPT method include a list of putatively causal genes with highest posterior inclusion probability (PIP) (Fig 1C) as well as shrunken factor loadings used to identify the subset of phenotypes most strongly influenced by the genes associated with each latent factor for revealing phenotype-specific gene regulation and improving the interpretability of the inferred factors (Fig 1D). We leave all technical details of the model, estimation and inference to the Methods section 1–4.

### FM-GPT accurately detects true causal genes across multiple traits while controlling false positives

To assess the performance of our proposed method, we conducted extensive simulations under a variety of scenarios and compared against representative competing methods from several categories. These included TWAS fine mapping methods GIFT [14] and MVIWAS [22], GWAS fine mapping methods PAINTOR [17] and CAVIAR [23], and a general-purpose Bayesian multivariate fine mapping method mvSuSIE [24] (see Table 1 for a comparison of the main characteristics of these methods). Among these methods, FM-GPT is the only method that allows phenome-wide analysis and accommodates phenotypes of mixed data types. In addition to prioritizing putative causal genes, FM-GPT simultaneously selects relevant phenotypes through shrunken factor loadings, improving interpretability. Details on how we implemented the competitive methods can be found in the S2 Methods.

**Table 1:** A comparison of main characteristics of representative GWAS/TWAS fine mapping methods/tools.

Methods	GWAS or TWAS fine mapping	Frequentist or Bayesian	Individual or summary based	Analysis of Multiple phenotypes?	Phenotypes of mixed data types?	Gene selection	Phenotype selection
GIFT [14]	TWAS	Frequentist	Both	N	N	Y	N
MVIWAS [22]	TWAS	Frequentist	Both	N	N	Y	N
FOGS [16]	TWAS	Frequentist	Summary	N	N	Y	N
FOCUS [15]	TWAS	Bayesian	Summary	N	N	Y	N
mvSuSIE [24]	General (GWAS/TWAS)	Bayesian	Individual	Y	N	Y	N
CAVIAR [23]	GWAS (SNP-level)	Bayesian	Summary	N	N	N (SNP only)	N
PAINTOR [17]	GWAS (SNP-level)	Bayesian	Summary	N	N	N (SNP only)	N
FM-GPT (ours)	General (GWAS/TWAS)	Bayesian	Individual	Y	Y	Y	Y

We primarily considered two simulation settings: (i) homogeneous causality (Scenario 1), where all latent phenotypic factors share the same causal genes; (ii) heterogeneous causality (Scenario 2), where causal genes differ across latent factors. Each simulation used a reference panel of size $n_{\text{1}}$=250, and GWAS sample size $n_{\text{2}}$=5000, reflecting typical TWAS settings. We considered both synthetic LD structure or LD structure directly obtained from the reference 1000 Genome [25], and used latent normal distribution or HAPGEN2 [26] to simulate the genotype data (see details in S1 Methods). We generated 10 genomic regions, each containing 10 genes with an average of 1–2 true causal genes per region. For expression generation, we assigned 10 cis-SNPs to each gene and all the cis-SNPs contribute to that gene’s genetically regulated expression, and the SNP sets were specified to be non-overlapping across genes, aligning well with the TWAS literature [4,5,11]. Phenotypes (q = 50) were generated from 1, 3 or 5 latent phenotypic factors and were either continuous or of mixed data types (continuous, binary and count). We further varied phenotypic heritability (i.e., the proportion of phenotypic variance explained by the gene expression) levels (1%, 3%, 5%) for comprehensive assessment. Detailed simulation setup including LD structure and genotype data generation, how genomic regions are selected, as well gene expression and phenotypic data generation can be found in the S1 Methods.

Fine mapping problem can be framed as a variable selection problem that aims to identify a parsimonious set of predictors (e.g. SNPs or genes) from a large number of correlated variables [27]. Thus, we evaluated the overall variable selection performance using Area Under the ROC Curve (AUC), which is independent of selection thresholds. We also compared the power and true FDR at a fixed nominal FDR level of 0.1 (FDR-adjusted p-values for the frequentist methods and Bayesian FDR for the Bayesian methods, see details in Methods section 4–5). Because CAVIAR and PAINTOR are designed for SNP-level GWAS fine mapping, their performance was assessed based on identification of cis-SNPs for the causal genes. GIFT and MVIWAS (equivalent to two-stage GIFT, see Methods section 5) are designed for single trait TWAS fine mapping, so we first applied factor analysis to the phenotypes (with one factor, three factors and five factors) and then performed gene-level analyses for each phenotypic factor, followed by meta-analysis (called “GIFT Factor” and “MVIWAS Factor”). To mitigate potential bias introduced by factor analysis, we additionally ran GIFT on each of phenotype independently and then meta-analyzed using Fisher’s method for comparison (called “GIFT Meta” and “MVIWAS Meta”). mvSuSIE accommodates multivariate phenotypes but assumes continuous outcomes only thus cannot handle mixed data types. Finally, we also evaluated the ability of FM-GPT to recover the correct factor loading structures using sensitivity, specificity and Youden index, in comparison with conventional exploratory factor analysis (EFA) and popular sparse PCA methods. For EFA, negligible loadings, e.g., < 0.1, are suppressed to zeros for fair comparison. For sparse PCA, five-fold cross-validation was used to determine the value of the sparsity tuning parameter. FM-GPT uses an infinite factor model with a gamma process shrinkage prior (see Methods section 2), which induces continuous shrinkage rather than exact sparsity, thus loadings are shrunk toward zero but are not generally estimated as exact zeros. We first applied a post-hoc unit-scaling procedure to normalize the loading vector (see S4 Methods) and then suppressed negligible loadings, e.g., < 0.1 to zeros as in EFA.

**Fig 2:**
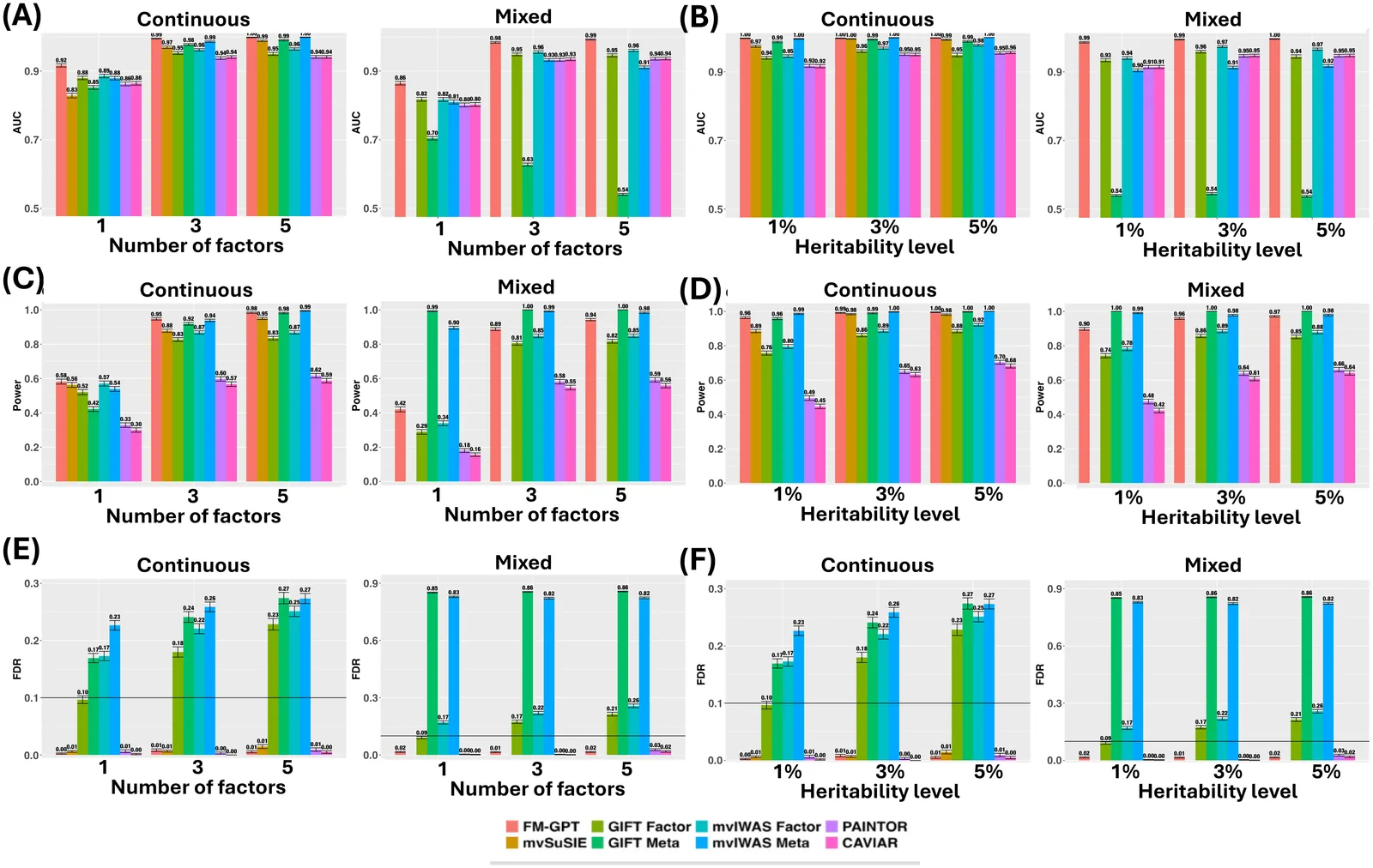
Simulation results for scenario 1 with homogeneous causality. (A) AUCs of all methods with varying number of factors for both continuous phenotypes only and phenotypes of mixed data types. (B) AUCs of all methods with varying heritability levels for both continuous phenotypes only and phenotypes of mixed data types. (C) Power of all methods at nominal FDR level of 0.1 with number of factors for both continuous phenotypes only and phenotypes of mixed data types. (D) Power of all methods at nominal FDR level of 0.1 with varying heritability levels for both continuous phenotypes only and phenotypes of mixed data types. (E) True FDR of all methods at nominal FDR level of 0.1 with number of factors for both continuous phenotypes only and phenotypes of mixed data types. (F) True FDR of all methods at nominal FDR level of 0.1 with varying heritability levels for both continuous phenotypes only and phenotypes of mixed data types.

**Fig 3:**
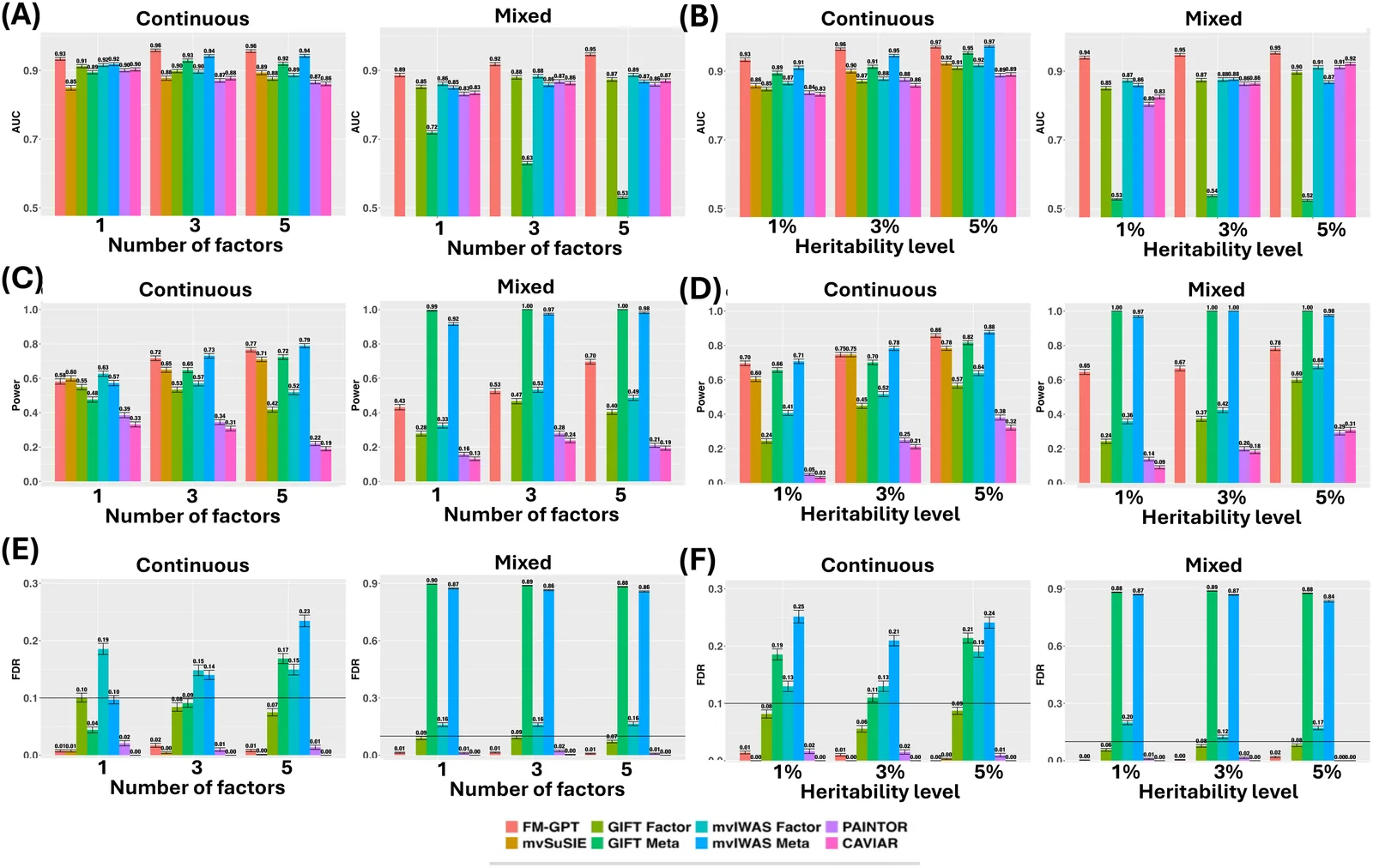
Simulation results for scenario 2 with heterogeneous causality. (A) AUCs of all methods with varying number of factors for both continuous phenotypes only and phenotypes of mixed data types. (B) AUCs of all methods with varying heritability levels for both continuous phenotypes only and phenotypes of mixed data types. (C) Power of all methods at nominal FDR level of 0.1 with number of factors for both continuous phenotypes only and phenotypes of mixed data types. (D) Power of all methods at nominal FDR level of 0.1 with varying heritability levels for both continuous phenotypes only and phenotypes of mixed data types. (E) True FDR of all methods at nominal FDR level of 0.1 with number of factors for both continuous phenotypes only and phenotypes of mixed data types. (F) True FDR of all methods at nominal FDR level of 0.1 with varying heritability levels for both continuous phenotypes only and phenotypes of mixed data types.

Under homogeneous causality case (Scenario 1), FM-GPT consistently achieved the highest AUC across varying numbers of latent factors and heritability levels, for both continuous and mixed-type phenotypes (Fig 2A and 2B), maintained a high statistical power (Fig 2C and 2D) while maintaining well-controlled FDR (Fig 2E and 2F). GIFT and MVIWAS based methods, especially GIFT Meta and MVIWAS Meta, showed high power but at the cost of substantially inflated false positives. PAINTOR and CAVIAR, which were designed for GWAS fine mapping, were generally underpowered. Although mvSuSIE effectively controlled false positives, its overall variable selection performance was inferior to FM-GPT, particularly when the number of latent factors was small or signal strength was low (i.e., low heritability). In the heterogeneous setting (Scenario 2), performance declined for all methods; however, FM-GPT remained the top-performing approach and demonstrated more pronounced advantages across all conditions (Fig 3A–3F). The results were largely consistent when we used reference LD structures and genotype data simulated with HAPGEN2 (S1 and S2 Figs). In light of the simulation results, we primarily compare FM-GPT to GIFT Factor, MVIWAS Factor and mvSuSIE for real data examples.

In evaluating the recovery of correct factor loading structures (S1a Table), sPCA has the benefit of enforcing sparsity in the loading matrix, leading to overall highest specificity. However, because of its high specificity and sparse structure, sPCA tends to be less sensitive and have too many false negatives, i.e., missing the true nonzero loadings. EFA on the other hand, has better sensitivity but lower specificity. Finally, FM-GPT has an improved specificity over EFA while in the same time maintaining the highest sensitivity among three methods. From the Youden Index (Sensitivity + Specificity – 1), we can see that in each case, FM-GPT outperforms both EFA and sPCA in discovering the latent loading structure. In addition, we also performed sensitivity analysis assessing how well FM-GPT recovers the true number of factors across simulation settings, i.e., the proportion of simulation replicates in which the true number of generated factors was contained within the model’s 95% highest-density interval for the inferred number of factors. Across the settings considered, FM-GPT accurately captured the true number of factors, supporting the robustness of the adaptive factor-selection procedure in FM-GPT (S1b Table).

In addition, we performed several sensitivity analyses to evaluate the robustness of FM-GPT. For mixed types of traits that include binary traits, we evaluated the impact of % of binary traits in mixed types, and the proportion of cases in binary traits on its performance. We also evaluated the impact of different covariance matrix structures for residuals (e.g., Compound Symmetry (CS), AR [1]) and mis-specified number of factors on its performance. Results are summarized in Supplementary (S2a–S2d Table). Overall, except for the case when factor-number misspecification can adversely affect performance, FM-GPT maintains high AUC and power with FDR under control, suggesting the robustness of the method.

### FM-GPT identifies genes with pleiotropic effects on brain-wide cortical thickness measures from structural MRI data in UKB

We applied FM-GPT to two real data applications in UK Biobank (UKB). In the first example, we investigated the genetic architecture of brain structure using structural MRI data from the UKB. Cortical thickness (CT), defined as the distance between the gray and white matter boundaries, is a key neuroanatomical phenotype linked to cognitive function, neurodevelopment, and a range of neurological and psychiatric disorders [28–30]. CT is also highly heritable, with both global and region-specific genetic influences [31,32]. Here, we aimed to leverage FM-GPT to perform TWAS and fine mapping analyses of regional CT across the entire cerebral cortex, with the goal of prioritizing putatively causal genes underlying brain-wide variations in cortical structure.

**Fig 4:**
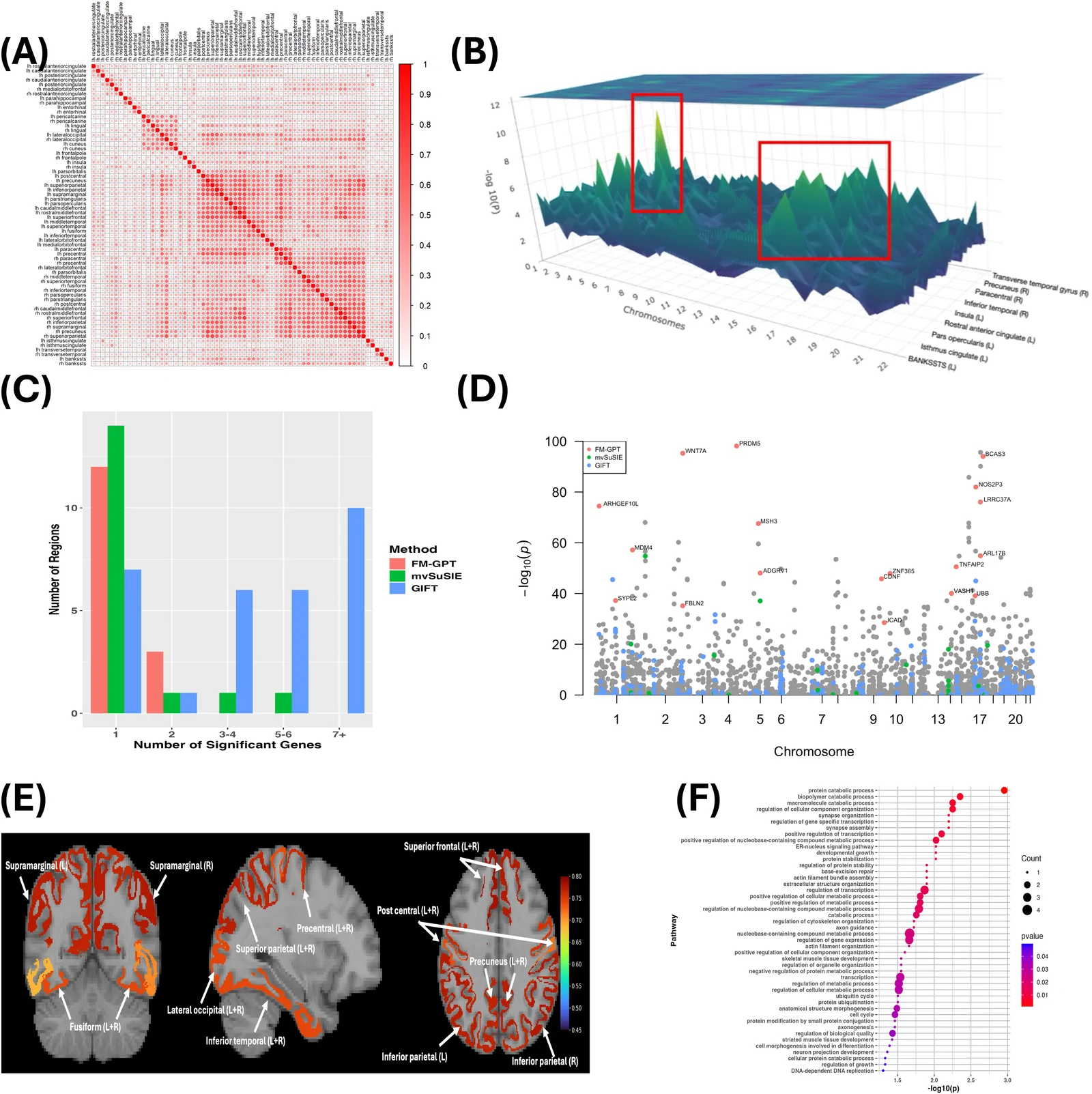
TWAS and fine mapping results for genetic study of 66 regional cortical thickness (CT) measures from UKB. (A) The Phenotypic correlation of the 66 regional CT measures; (B) 3D Manhattan plot of TWAS p-values (most significant genomic regions highlighted); (C) Proportion of regions that harbor different number of causal genes by different methods; (D) Manhattan plot of meta-TWAS Fisher’s method p-values and the putative causal genes identified by each fine mapping method; (E) Brain regions with the largest factor loadings; (F) Pathway enrichment analysis on FM-GPT selected genes results (p < 0.05).

We analyzed CT measures of 66 cortical regions (see S3 Table for a list of these brain regions) defined by the Desikan-Killiany Atlas [33], derived using FreeSurfer [34]. After restricting to genetically unrelated individuals with complete genetic and covariate data (age, sex, body mass index (BMI) and top 10 genetic principal components), the final sample included n_2_ = 26,124 individuals. As expected, regional CT measures were highly correlated, reflecting substantial shared variation across the cortex (Fig 4A).

We first conducted univariate TWAS using 13 brain tissues from GTEx(1), for each of the 66 regions (see Fig 4B for the 3D Manhattan plot; S3 Table) adjusting for covariates including age, sex, BMI and the top 10 principal components. We then aggregated evidence across regions using Fisher’s method and selected 760 genes (p < 1e-8) for downstream analysis. Using LDetect [21], we selected 355 genomic regions containing at least one TWAS significant gene as regions of interest which we would fine map next (S4 Table). Based on the adaptation scheme (see S5 Methods) and the diagnostic plots (trace plot in S3 Fig and phenotype correlation heatmap and scree plot in S5 Fig), FM-GPT determined one factor to be fit for this example.

FM-GPT identified 18 putative causal genes across 16 genomic regions at a Bayesian false discovery rate (BFDR) of 0.15 (Table 2). In comparison, mvSuSIE identified 25 genes, GIFT and MVIWAS (assuming a single factor) identified 164 and 174 genes, respectively at the same FDR cutoff (two genes selected by all methods; S5 Table). Notably, FM-GPT demonstrated substantially greater specificity, with a higher proportion of regions containing only 1–2 prioritized genes and greatly narrowed down the set size of putatively causal genes by 28–90% compared with other methods (Fig 4C and Table 2). Moreover, genes prioritized by FM-GPT were supported by stronger TWAS signals overall, whereas competing methods frequently selected genes with weak or non-significant associations, suggesting inflated false positives (Fig 4D).

**Table 2:** Summary of fine mapping results for the 355 regions by different methods in the cortical thickness example.

Methods	Number of significant genes from fine mapping (FDR < 0.15)	Number of regions with 1 causal gene	Number of regions with 2 causal genes	Number of regions with 3–4 causal gene	Number of regions with 5–6 causal gene	Number of regions with 7 + causal gene
FM-GPT	18	12	3	0	0	0
mvSuSIE	25	14	1	1	1	0
GIFT	164	7	1	6	6	10
MVIWAS	174	45	15	14	1	4

Among them, FM-GPT identified critical pleiotropic genes localized to a region on chromosome 17, including *BCAS3*, *LRRC37A*, *NOS2P3, ARL17B* and *UBB,* participated in neuronal morphology and cortical organization, highlighting a coordinated genetic program influencing distributed cortical architecture [35,36]. Importantly, FM-GPT revealed that all these genes act through a latent factor encompassing 46 cortical regions (S6 Table, see the regions with highest loadings in Fig 4E), indicating a common genetic program influencing widespread cortical architecture.

This finding highlights a key advantage of FM-GPT: rather than analyzing each region independently or relying on global summary measures, our approach jointly models brain-wide phenotypes and directly identifies shared putatively causal genes underlying coordinated variation across regions. In contrast to prior studies that examined global mean CT or region-specific GWAS separately [35,36], FM-GPT provides a unified and interpretable framework for uncovering the genetic basis of distributed brain structure. Finally, pathway analysis of the prioritized genes using Gene Ontology Biological Process database [37] revealed significant enrichment in biological processes related to protein catabolic process, protein ubiquitination and synaptic function (Fig 4F and S7 Table), underscoring the role of neuronal maintenance and synaptic integrity in shaping cortical morphology.

### FM-GPT identifies genes that influence multiple medical conditions derived from EHR data

In a second application, we applied FM-GPT to fine-map genetic associations across a broad spectrum of disease phenotypes derived from EHR data in UKB. EHR data provide a powerful resource for large-scale phenome-wide analyses but present substantial analytical challenges due to their high dimensionality, heterogeneous data types (e.g., binary diagnoses, counts, continuous traits), and complex correlation structure across diseases. These features limit the applicability of conventional fine-mapping approaches, which typically assume homogeneous phenotype types and analyze each trait independently.

We extracted 19,190 ICD10-based disease codes and performed systematic preprocessing to derive 1,403 phenotypes of mixed data types (primarily binary and count), grouped into 16 major disease categories (e.g., circulatory, metabolic, digestive, and respiratory systems) [38]. To ensure adequate statistical power and avoid extreme case–control imbalance, we retained 169 phenotypes with prevalence >1% among n_2_ = 245,687 individuals for downstream TWAS and fine-mapping analyses (S8 Table; see detailed steps on processing these EHR-derived phenotypes in S3 Methods).

**Fig 5:**
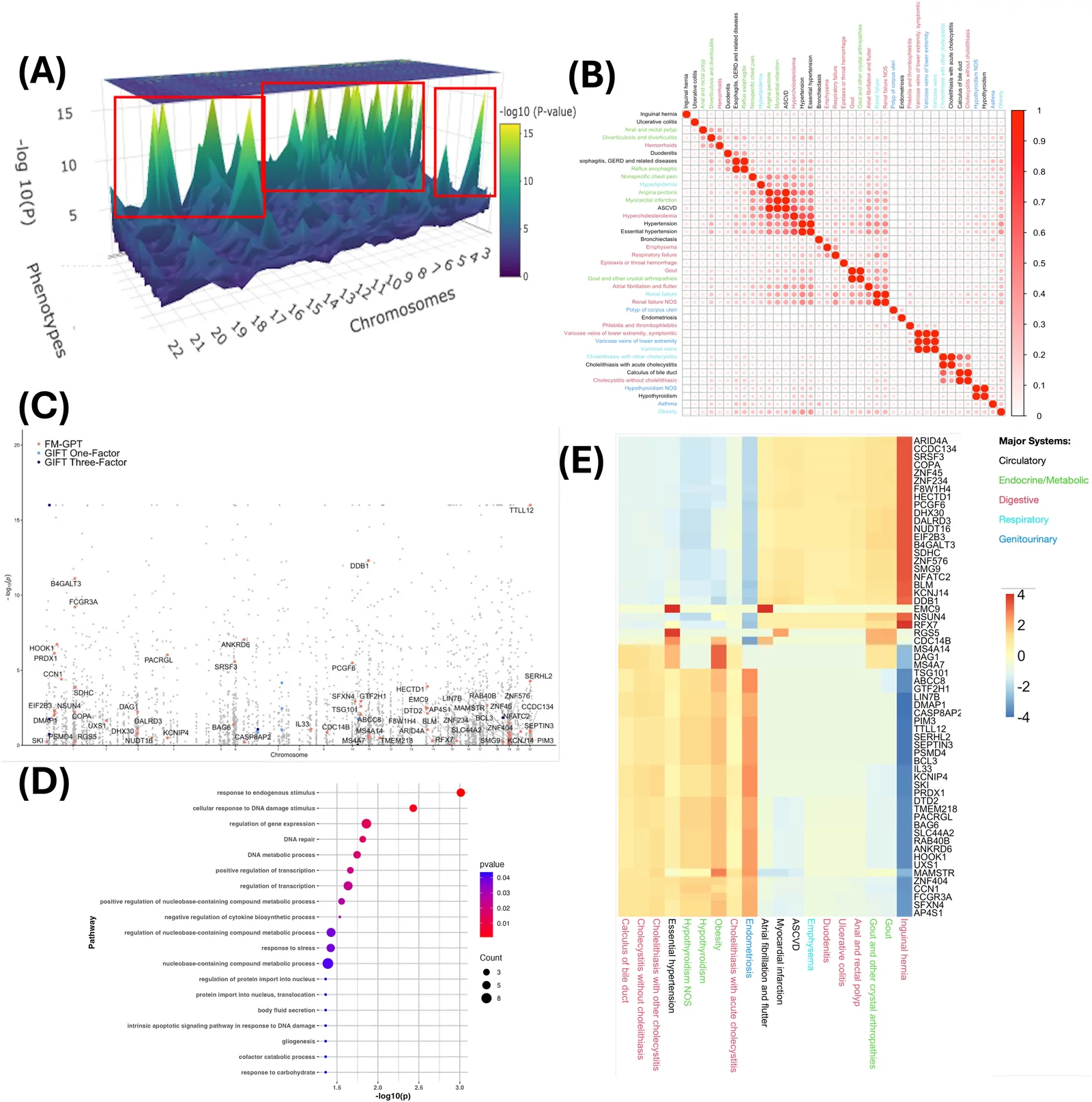
TWAS and fine mapping results for genetic study of EHR-derived phenotypes from UKB. (A) 3D Manhattan plot of TWAS p-values (most significant genomic regions highlighted); (B) The Phenotypic correlation of the 39 EHR-derived medical conditions; (C) Manhattan plot of meta-TWAS Fisher’s p-values and the putative causal genes identified by each fine mapping method; (D) Pathway enrichment analysis on FM-GPT selected genes results (p < 0.05); (E) Heatmap of identified putative causal genes and the loadings of phenotypes they target at in the dominating factor with the largest factor-loading vector norm. Note that the heatmap is a visual summary only and does not represent the full set of factors by which a gene may be associated with the phenotypes.

We first conducted univariate TWAS for each phenotype using all 50 tissues from GTEx(1), adjusting for key covariates, e.g., age, sex, BMI (Fig 5A and S9 Table). We then focused on 39 phenotypes with at least one gene showing genome-wide significant association (p < 1e-6), spanning major disease domains including circulatory, digestive, metabolic, respiratory and genitourinary systems. These phenotypes exhibited substantial correlation both within and across clinical categories (Fig 5B), motivating a joint modeling framework. We aggregated TWAS signals across phenotypes using Fisher’s method and defined 297 genomic regions containing at least one significant gene (S10 Table) for fine mapping. Based on the diagnostic plots (phenotype correlation heatmap and scree plot in S5 Fig), FM-GPT determined three factors to be fit for this example.

FM-GPT identified 60 putatively causal genes at a BFDR of 0.15 (Fig 5C). Since the phenotypes are of mixed data types (primarily binary and count) in this example, mvSuSIE failed to run. GIFT and MVIWAS with one phenotypic factor or three phenotypic factors (see Methods section 5), as well as univariate GIFT independently on top 10 phenotypes ranked by TWAS signals were applied for comparison. As compared to FM-GPT, GIFT and MVIWAS tend to select genes with weak or non-significant TWAS associations (Fig 5C and S10 Table). GIFT on univariate phenotype has severe inflation of false positives with hundreds to thousands of genes detected as putatively causal, which undermines the reliability and interpretability of fine-mapping results (S10 Table). Notably, our method identified dominant factors targeted by different genes exhibited similar phenotype-loading profiles. For each gene, the dominant latent factor, defined as the factor with the largest factor-loading vector norm, captured structured heterogeneity across disease domains, with two major axes of variation emerging (Fig 5E and S11 Table). One axis was driven by cardiovascular and inflammatory conditions, including atrial fibrillation, myocardial infarction, and ulcerative colitis, whereas the opposing axis was enriched for metabolic and hepatobiliary phenotypes, such as gallstone-related disorders, hypothyroidism, and obesity. Importantly, genes loading onto these factors exhibited distinct functional profiles. Genes associated with the cardiovascular-inflammatory axis were enriched for roles in transcriptional regulation, RNA processing, and genome maintenance (e.g., *ARID4A*, *SRSF3*, *BLM*, *DDB1*) [39,40], whereas those associated with the metabolic-hepatobiliary axis included key immune and inflammatory regulators (e.g., *IL33*, *FCGR3A*, *BCL3*) [41,42] (Fig 5D and S12 Table). These results suggest a pleiotropic genetic architecture in which shared regulatory pathways influence multiple disease domains, while also allowing for directionally distinct effects across phenotypic groups. We also performed additional sensitivity analysis to evaluate the stability of the inferred pleiotropic genetic architecture with different random initializations or random 90% subsampling. The factor-loading structures are reasonably robust to both initialization choices and moderate sample perturbation (S4 Fig).

This pattern is suggestive of a potential immune-metabolic trade-off [43], whereby genetic regulation may coordinate energy allocation between immune function and metabolic processes, however, this remains hypothesis-generating as the directionality of latent factor loadings could be inherently arbitrary. More broadly, these results illustrate how coordinated yet heterogeneous genetic effects across disease domains can be captured through joint modeling. Such patterns are difficult to detect using conventional single-trait or univariate fine-mapping approaches. By simultaneously capturing shared and phenotype-specific genetic influences across diverse and mixed-type traits, FM-GPT provides a flexible and interpretable framework for investigating the genetic architecture of complex, multi-system diseases.

## Discussion

In this study, we developed FM-GPT, a Bayesian fine-mapping framework for phenome-wide TWAS that jointly prioritizes putatively causal genes and latent phenotype factors when multiple correlated outcomes are analyzed together. By combining Bayesian variable selection with supervised factor analysis, FM-GPT is designed to recover both shared and phenotype-specific patterns of genetic regulation across traits while retaining interpretability at the phenotype level. Our simulation studies demonstrate that leveraging phenome-wide information in a joint model can accurately recover the true causal genes without inflating the false positives. In UKB applications, FM-GPT greatly narrowed down the set size of putatively causal genes compared to other methods, and highlighted genes with pleiotropic effects on regional cortical thickness measures across the cerebral cortex, as well as genes that influence multiple EHR-derived medical conditions spanning circulatory, metabolic, digestive, respiratory and genitourinary systems, and reveal potential immune-metabolic gene regulatory trade-off. These results demonstrate that FM-GPT enables the discovery of shared genetic architecture across the phenome, uncovering both universal and phenotype-specific mechanisms that are not apparent from single-trait analyses or conventional fine-mapping approaches.

Existing fine-mapping approaches are largely designed for single-phenotype analyses, limiting their direct applicability to phenome-wide settings. Applying these methods independently across traits introduces a substantial multiple testing burden and often leads to reduced power, particularly when phenotypes are correlated [44,45]. Conversely, dimension-reduction approaches such as factor analysis or principal component analysis applied a priori may oversimplify heterogeneous trait architectures, and the resulting latent factors are not guaranteed to align with underlying genetic effects [46]. FM-GPT bridges these two extremes by jointly modeling latent factor structure and gene selection within a unified framework. By allowing genetic signals to inform factor construction, the method identifies latent phenotypic structures that are more directly linked to underlying biology. This joint modeling strategy improves power to detect shared genetic effects while preserving heterogeneity across traits. In addition, FM-GPT accommodates mixed data types and enforces shrunken factor loadings, enhancing both interpretability and scalability for complex phenome-wide analyses.

The FM-GPT framework can be extended in several important directions. Firstly, incorporating multi-omics QTL resources beyond expression QTLs used in TWAS, such as splicing QTLs, methylation QTLs, and chromatin accessibility QTLs, could substantially improve resolution in prioritizing regulatory mechanisms and putatively causal genes [47–49]. Secondly, though our current implementation integrates expression prediction models across multiple tissues to maximize statistical power, future extensions could explicitly incorporate tissue- and cell-type-specific regulatory architectures. Such approaches may enhance biological interpretability, particularly for traits with well-defined cellular contexts, as demonstrated in recent single-cell and tissue-resolved genetic studies [50,51]. Thirdly, the current model does not explicitly account for causal relationships among phenotypes. Extending the framework to jointly model genetic effects and directed dependencies between traits, potentially through integration with causal inference or structural equation modeling approaches, could provide deeper insight into mechanisms of pleiotropy and mediation [46,52,53]. Lastly, further methodological development is warranted to scale to increasingly large phenomic datasets. In particular, extending FM-GPT to operate directly on GWAS summary statistics would broaden its applicability, while variational Bayesian approximations could offer additional gains in computational efficiency relative to Gibbs sampling [54]. For settings involving very large numbers of genes, incorporating principled feature-screening or preselection strategies may further improve scalability [55]. As phenomic and multi-omics resources continue to expand, methods that jointly model genetic effects across diverse traits will become increasingly critical. By integrating information across traits while preserving heterogeneity, FM-GPT provides a flexible and extensible framework that will play an increasingly important role in translating association signals into biological meaningful insights. The R package to implement FM-GPT method is freely available at www.github.com/tacanida/fm-gpt.

## Methods

In the following sections, we present an overview of the FM-GPT method, including the model formulation, prior specification and the data augmentation strategy for handling mixed data types, as well as the procedure for selecting putatively causal genes. We also describe the data and the cohort used in the two application examples.

### 1. Model of FM-GPT

Consider a reference eQTL dataset (e.g. GTEx) with $n_{\text{1}}$ individuals and a GWAS dataset with $n_{\text{2}}$ individuals and q measured phenotypes. Following standard convention in fine mapping [27], we first identify genomic regions of interest based on phenome-wide TWAS results (e.g., via meta-analysis of univariate TWAS p-values). Suppose there are T regions of interest identified, and let $p_{t}$ denote the number of genes in t-th region (1≤t≤T). We consider genes within each genomic region (based on LD blocks defined by LDetect [21]) as potentially predictors of the phenotypes under study, analyzing one region at a time. Without loss of generality, we will introduce the model formulation (section 1), prior specification (section 2) and data augmentation (section 3) using t-th genomic region as an example, and the same setting applies to all T regions of interest. For selecting putative causal genes using Bayesian false discovery rate (section 4), we will consider all genes and all genomic regions.

Let $x_{j}$ denote the $n_{\text{1}}$-vector of gene expression levels for the j-th gene in the reference dataset, for $j=\text{1},\text{2},\ldots ,p_{t}$. Let $Z_{j}^{R}$ denote the $n_{\text{1}}$x $g_{j}$ genotype matrix of the $g_{j}$ cis-SNPs for the jth gene in the reference dataset and $Z_{j}^{G}$ denote the corresponding $n_{\text{2}}$x $g_{j}$ genotype matrix in the GWAS dataset. Let $\gamma_{j}$ denote the $g_{j}$x 1 vector of cis-SNP effect sizes (i.e., the weight vector) for the jth gene and let $\hat{x_{j}}=Z_{j}^{G}{\hat{\gamma }}_{j}\;$the corresponding predicted GReX in the GWAS dataset for $j=\text{1},\text{2},\ldots ,p_{t}$.

Let $Y=\{y_{ik};i=\text{1},\text{2},\ldots n_{\text{2}},k=\text{1},\text{2},\ldots ,q\}$ denote the $n_{\text{2}}$x q phenotype matrix in the GWAS dataset, the q phenotypes are typically correlated and are assumed to be represented by m latent factors. Let $B=\{\beta_{jl};j=\text{1},\text{2},\ldots p_{t},l=\text{1},\text{2},\ldots ,m\}$ denote the $p_{t}$x m matrix of gene effects on phenotype factors. Let F denote the $n_{\text{2}}$x m matrix of factor scores, where each column $f_{l}$ corresponds to the lth factor (l=1,2,…,m), and let Λ denote the qx m factor loading matrix, where each column $\lambda_{l}$ represents the loadings for the lth factor. The phenotypes may be of mixed data types, such that each column of Y can be continuous or discrete. We assume that each column of $Z_{j}^{R}$ and $Z_{j}^{G}$ has been standardized to have mean zero and unit variance.

Fig 1B shows a DAG of the full joint model. The joint model considers the following equations for each t-th (1≤t≤T) genomic region (the same model applies over all T regions):


$$\begin{array}{c} \begin{array}{c} x_{j}=Z_{j}^{R}\gamma_{j}+\varepsilon_{j},\;\varepsilon_{j}\sim N\left(\text{0},\sigma_{j}^{\text{2}}I_{n_{\text{1}}}\right),\;j=\text{1},\text{2},\ldots ,p_{t}; \end{array} \end{array}$$ (1)

$$\begin{array}{c} \begin{array}{c} f_{l}=\sum_{j=\text{1}}^{p_{t}}\beta_{jl}\hat{x_{j}}+\epsilon_{l},\;\epsilon_{l}\sim N\left(\text{0},\omega_{l}^{\text{2}}I_{n_{\text{2}}}\right),\;l=\text{1},\text{2},\ldots ,m; \end{array} \end{array}$$ (2)

$$\begin{array}{c} \begin{array}{c} {\tilde{Y}}_{i}=\Lambda F_{i}+{\text{E}}_{i}^{\prime },\;E_{i}\sim N_{q}(\text{0},\Sigma ),\;i=\text{1},\text{2},\ldots ,n_{\text{2}}, \end{array} \end{array}$$ (3)

where each element in ${\tilde{Y}}_{i}:$


$$\begin{array}{c} {\tilde{y}}_{ik}=\;\left\{ \begin{array}{c} y_{ik},\;if\;y_{ik}\;is\;continuous\\ \frac{y_{ik}-\text{0}.\text{5}\xi_{ik}}{\psi_{ik}},\;if\;y_{ik}\;is\;discrete \end{array}\right. \end{array}$$ (4)

for; k=1,2,…,q. $\tilde{Y_{i}}$ is the i-th row of $\tilde{Y}$, $E_{i}$ is the i-th row of E and $F_{i}$ is the i-th row of F. $\tilde{Y^{\prime }}$ and ${\text{E}}^{\prime }$ denote the transpose of $\tilde{Y}$ and E, respectively. Equation (4) defines the augmented response used in the data augmentation scheme (see Section 3), whereas equation (3) links this augmented response to the continuous latent factors through the factor model. Note that equation (4) should not be interpreted as a deterministic transformation of an observed discrete random variable into a Gaussian random variable. Instead, for discrete outcomes, we introduce a Polya-Gamma auxiliary variable (ψ). Conditional on ψ, the likelihood contribution for the natural parameter θ, in this case ΛF in equation (3), is proportional to a Gaussian kernel. Although this pseudo-response is not itself assumed to be marginally Gaussian, conditional on the Pólya–Gamma auxiliary variable, the likelihood contribution for the natural parameter θ has a Gaussian kernel. This conditional Gaussian representation allows continuous and discrete outcomes to be handled within a unified latent-factor framework while preserving the original discrete likelihood after integrating over the auxiliary variable. Thus, equation (3) should be understood as a conditionally Gaussian representation of the augmented likelihood, where the left-hand side denotes either the observed continuous response or the augmented continuous pseudo-response induced by the Pólya–Gamma data augmentation scheme. More details can be found in the Section 3.

Unlike standard unsupervised factor analysis, where latent factors are learned solely from the outcome matrix and a single global factor structure is often assumed across all features, FM-GPT performs region-specific factorization for each genomic region targeted for fine mapping. Specifically, equations (1)–(3) are applied separately to each region (t), and the outcome matrix Y is jointly modeled with the predicted gene expression matrix X for genes within that region. The latent factors are constructed as linear combinations of region-specific genetically regulated expression (GReX), as defined in equation (2). Thus, the factor space is constrained to lie within the span of candidate GReX variables in the locus. This is a strong assumption but also an intentional modeling choice. FM-GPT is not designed to discover latent phenotypic structure in a fully unsupervised manner; rather, it aims to identify latent axes of phenotypic variation that are driven by genetically regulated transcriptional mechanisms. By anchoring the factors to local GReX, FM-GPT focuses dimension reduction on gene-regulated variation within the region, improving biological interpretability and facilitating causal gene prioritization. Because different genomic regions contain different genes and may influence different subsets of traits through distinct regulatory mechanisms, FM-GPT allows both latent factors and factor loadings to vary across regions. This region-specific formulation avoids imposing an artificial common factor alignment across loci and better reflects the local, context-dependent regulatory architecture that fine mapping seeks to resolve. FM-GPT also differs from traditional supervised factor models in that it uses region-specific GReX variables and embeds the resulting latent representation within a Bayesian fine-mapping framework.

Equation (1) specifies a per-gene regression model describing the relationship between the expression of the jth gene and its cis-SNP regulators in the reference panel dataset (i.e., the training stage in TWAS method [3]). $\varepsilon_{j}$ denotes the error that follows $N\left(\text{0},\sigma_{j}^{\text{2}}I_{n_{\text{1}}}\right)$, where $\sigma_{j}^{\text{2}}$ is a gene specific variance. As the number of cis-SNPs $g_{j}$ is typically large, we adopt a Bayesian sparse linear model with a spike-and-slab prior on $\gamma_{j}$ to select nonzero effects (see section 2 for prior specification). $\gamma_{j}$ can also be estimated from the reference eQTL data. When multiple tissues are available in the training data, equation (1) can be extended by incorporating a multivariate spike-and-slab prior to jointly model SNP effects across tissues, thereby improving statistical power [9]. The training stage can be computationally intensive when many SNPs map to each gene, and access to raw reference panel data may be limited. As an alternative, users can directly leverage pre-trained weight matrices from existing models (e.g., PrediXcan [5], MultiXcan [8] or UTMOST [9]) to impute GReX in the GWAS dataset.

Equation (2) specifies a per-factor regression model describing the relationship between GReX for each gene and the latent phenotype factors in the GWAS dataset. The fine-mapping problem can be framed as a variable selection problem that aims to identify a parsimonious set of predictors (e.g., SNPs or genes) from a large number of correlated variables. In our setting, the goal is to identify putative causal genes that influence multiple phenotypes among all genes located within a genomic region of interest. $\epsilon_{l}$ denotes the error term assumed to be independently and identically distributed as $N\left(\text{0},\omega_{l}^{\text{2}}I_{n_{\text{2}}}\right)$, where $\omega_{l}^{\text{2}}$ is the variance of the lth factor. $\beta_{jl}$ is the parameter of interest in fine mapping, as it characterizes gene effects on phenotypes and enables the identification of true causal genes from a large set of candidates, including those with null effects. We consider an omnibus test of gene effects over all factors, defined as $H_{\text{0}}:{⋂}_{l}\left\{\beta_{jl}=\text{0}\right\}$ vs. $H_{a}:{⋃}_{l}\left\{\beta_{jl}\neq \text{0}\right\}\;$for 1≤j≤p. As our method is formulated within a full Bayesian hierarchical framework, we use posterior inclusion probabilities and the corresponding Bayesian false discovery rate to prioritize putative causal genes [56] (see Section 4).

Equation (4) defines the augmented response used to accommodate both continuous and discrete traits in the data augmentation scheme (see Section 3) and equation (3) specifies a factor model that describes the relationship between a large number of responses and a smaller number of latent phenotype factors. We impose shrinkage-inducing priors on Λ to select factor loadings (see Section 2). As in standard latent factor models, we assume that the error matrix $E_{i}\sim N_{q}(\text{0},\Sigma )$, where Σ is assumed to be a diagonal matrix $diag\left(\tau_{\text{1}}^{\text{2}},\ldots ,\tau_{q}^{\text{2}}\right)$ with $\tau_{k}^{\text{2}}$ representing the phenotype-specific variance for the k-th phenotype. To relax the strong independent residual covariance assumption in Σ, we also considered a compound-symmetric (CS) and an AR [1] covariance structure and performed sensitivity analysis in simulations to evaluate its impact. The parameters $\xi_{ik}$ and $\psi_{ik}$ arise from the data augmentation procedure described in Section 3 data augmentation strategy.

Compared with multi-stage modeling approaches, our joint model offers several advantages. First, it enables joint modeling of multiple genes and multiple phenotypes, along with multi-level selection at the phenotype, factor, and gene levels, which facilitates the identification of complex and potentially heterogeneous regulatory patterns between genes and phenotypes. Secondly, unlike commonly used unsupervised factor models—where latent factors are derived independently of predictors—our approach employs a supervised factor model in which the mapping from phenotypes to latent factors is partially driven by putative causal genes. In addition, shrinkage in the factor loadings promotes the selection of relevant phenotypes and improves the interpretability of the latent factors. Third, our joint model can accommodate mixed types of phenotypes, addressing a critical challenge in genetic studies involving multiple traits [27].

The primary parameters of interest in the model are Λ and B. The matrix B  is used to identify which genes are inferred to be putatively causal and to determine the factors they influence. The loading matrix Λ identifies the subset of the phenotypes of that load onto each latent factor.

### 2. Prior setting

For the full model, we consider three shrinkage-inducing priors to encourage selection of cis-SNP effects on gene expression ($\gamma_{j}=(\gamma_{\text{1}},\ldots ,\gamma_{g_{j}})$) in the reference dataset, selection of gene effects on latent factors ($\beta_{jl}$), and selection of factor loadings of observed phenotypes on each latent factor ($\lambda_{l}$).

For each element in $\gamma_{j}$, we propose a standard spike-and-slab prior for the effect of d-th SNP on gene expression, $\gamma_{d}$*,* for $d=\text{1},\text{2},\ldots ,g_{j}$, as in many Bayesian TWAS models [3,13]:


$$\begin{array}{c} \gamma_{d}\sim I_{d}^{\left(\gamma \right)}\delta_{\text{0}}(.)+\left(\text{1}-I_{d}^{\left(\gamma \right)}\right)N\left(\text{0},\upsilon_{j}^{\text{2}}\sigma_{j}^{\text{2}}\right);\;I_{d}^{\left(\gamma \right)}\sim Ber\left(\pi^{\left(\gamma \right)}\right). \end{array}$$

$\delta_{\text{0}}(.)$is the point mass at zero. The spike-and-slab prior enables exact variable selection by shrinking small effect sizes to zero through a mixture of a point mass at zero (spike) and a continuous distribution over the real line (slab). For the hyperparameters, we assume standard uniform and Jeffrey’s priors: $\pi^{\left(\gamma \right)}\sim Unif(\text{0},\text{1}),\;\upsilon_{j}^{\text{2}}\propto \text{1}/\upsilon_{j}^{\text{2}}$.

For $\beta_{jl}$, we specify the indicator variable priors (equivalent to spike-and-slab priors [57,58]) to select putative causal genes for each phenotype factor:


$$\begin{array}{c} \beta_{jl}=I_{j}^{\left(\beta \right)}U_{jl}^{\left(\beta \right)}b_{jl};\;b_{jl}|s^{\text{2}},\omega_{l}^{\text{2}}\sim N\left(\text{0},s^{\text{2}}\omega_{l}^{\text{2}}\right);\;I_{j}^{\left(\beta \right)}\sim Ber\left(\pi_{\text{1}}^{\left(\beta \right)}\right);\;U_{jl}^{\left(\beta \right)}\sim Ber\left(\pi_{\text{2}}^{\left(\beta \right)}\right). \end{array}$$

$I_{j}^{\left(\beta \right)}$and $U_{jl}^{\left(\beta \right)}$ are two indicator variables serving distinct roles in feature selection: $I_{j}^{\left(\beta \right)}$ controls gene-level selection and $U_{jl}^{\left(\beta \right)}$ determines whether a gene is active for a specific factor. $b_{jl}$ represents the underlying effect of the j-th gene on the lth factor. For the hyperparameters, we assume standard uniform and Jeffrey’s priors: $\pi_{\text{1}}^{\left(\beta \right)}\sim Unif(\text{0},\text{1})$, $\pi_{\text{2}}^{\left(\beta \right)}\sim Unif(\text{0},\text{1})$, $s^{\text{2}}\sim \text{1}/s^{\text{2}}$, $\omega_{l}^{\text{2}}\sim \text{1}/\omega_{l}^{\text{2}}$.

For $\lambda_{l}$, we apply a gamma process shrinkage prior [59] to shrinkage the factor loadings, while allowing the number of factors to go to infinity. For k=1,2,…,q and l=1,2,…,m, we assume the following prior on each loading coefficient:


$$\begin{array}{c} \lambda_{kl}|\phi_{kl},\eta_{l}\sim N(\text{0},\phi_{kl}^{-\text{1}}\eta_{l}^{-\text{1}});\;\phi_{kl}\sim Ga\left(\frac{\text{3}}{\text{2}},\frac{\text{3}}{\text{2}}\right);\;\eta_{l}=\prod_{r=\text{1}}^{l}\vartheta_{r}, \end{array}$$

where Ga refers to gamma distribution, $\vartheta_{\text{1}}\sim Ga(a_{\text{1}},\text{1})$ and $\vartheta_{h}\sim Ga(a_{\text{2}},\text{1})$ for h≥2. In our prior setting, we assume $a_{\text{1}}>\text{1}$ and $a_{\text{2}}$>1, which induces stochastic shrinkage in $\eta_{l}$ and encourages progressively stronger shrinkage for higher-indexed factors. The parameters $\phi_{kl}$ and $\eta_{l}$ together define a global-local shrinkage structure on the loadings, preventing any individual loading vector or element from becoming excessively large. Note that gamma process shrinkage prior induces continuous shrinkage rather than exact sparsity, therefore the factor loadings $\lambda_{kl}$ are shrunk toward zero but are not generally estimated as exact zeros. To improve interpretability, we applied a post-hoc unit-scaling procedure at each Gibbs sampling iteration by normalizing the loading vector for each factor (see S4 Methods). After scaling, negligible loadings, e.g., < 0.1 were suppressed to zeros, similar to common practice in EFA. Finally, the effective number of factors is learnt from the data through an adaptive step within the Gibbs sampler. In addition, we also provided a practical guideline for determining the number of factors in our method (see S5 Methods).

### 3. Data augmentation strategy for mixed data types

To accommodate phenotypes of mixed types (e.g., continuous, binary, multinomial and count), we introduce a data augmentation strategy [60,61]. For $i=\text{1},\text{2},\ldots ,n_{\text{2}}$; k=1,2,…,q, we define the augmented response ${\tilde{y}}_{ik}$ as:


$$\begin{array}{c} {\tilde{y}}_{ik}=\;\left\{ \begin{array}{c} @ly_{ik},\;if\;y_{ik}\;is\;continuous\\ \frac{y_{ik}-\text{0}.\text{5}\xi_{ik}}{\psi_{ik}},\;if\;y_{ik}\;is\;discrete \end{array}\right.\;\;\;\;, \end{array}$$

where $\xi_{ik}$ may vary depending on the data type of $y_{ik}$. For example, for a binary outcome, $\xi_{ik}=\text{1}$; for a multinomial outcome, $\xi_{ik}=M_{k}$, where $M_{k}$ is the total number of outcomes; for count outcome using negative binomial distribution, $\xi_{ik}=y_{ik}+r_{k}$, where $r_{k}$ is the target number of successes in negative binomial model. The key idea of the Polya-Gamma data augmentation strategy [60,61] is to introduce an auxiliary variable $\psi_{ik}$~$PG\left(\xi_{ik},\text{0}\right)$, which augments the likelihood for discrete outcomes so that conditional on $\psi_{ik}$, the likelihood contribution for the natural parameter becomes proportional to a Gaussian kernel. This construction yields the continuous pseudo-response used on the left-hand side of equation (3) and links the original discrete outcome to the continuous latent factor model. For discrete outcomes, including binary, multinomial, negative binomial-based count outcomes, the likelihood can be written in the logistic form: $p_{k}\left(y_{ik}|\theta_{ik}\right)=C\frac{{\left\{\text{exp}\left(\theta_{ik}\right)\right\}}^{y_{ik}}}{{\left\{\text{1}+\text{exp}\left(\theta_{ik}\right)\right\}}^{\xi_{ik}}}$, where C is a normalizing constant and $\theta_{ik}=\Lambda_{\text{k}}^{\prime }F_{i}$ is the natural parameter in our model. It can be shown that for continuous outcomes: $p\left(\theta_{ik}|y_{ik}\right)\propto p\left(\theta_{ik}\right)\text{exp}\left\{-\frac{\text{1}}{\text{2}}{\left(\theta_{ik}-y_{ik}\right)}^{\text{2}}\right\}$, and for discrete outcomes: $\;p\left(\theta_{ik}|{y_{ik},\;\psi }_{ik},\xi_{ik}\right)\propto p\left(\theta_{ik}\right)\text{exp}\left\{-\frac{\psi_{ik}}{\text{2}}{\left(\theta_{ik}-\frac{y_{ik}-\text{0}.\text{5}\xi_{ik}}{\psi_{ik}}\right)}^{\text{2}}\right\}$. We can unify the continuous and discrete outcomes with the following conditional density of $\theta_{ik}:p\left(\theta_{ik}|{y_{ik},\;\psi }_{ik},\xi_{ik}\right)\propto p\left(\theta_{ik}\right)\text{exp}\left\{-\frac{\psi_{ik}}{\text{2}}{\left({\theta_{ik}-\tilde{y}}_{ik}\right)}^{\text{2}}\right\}$, which implies the likelihood is the kernel of the Gaussian density. Full technical details of the derivation can be found in [60,61]. This data augmentation provides a unified likelihood representation for continuous and discrete phenotypes, enabling the use of conjugate conditional posteriors within a Gibbs sampling framework.

All the parameters in the full model have closed-form posterior distributions, so we use Gibbs sampling algorithm to update all the parameters (see S4 Gibbs sampler).

### 4. Selection of the putative causal genes using Bayesian false discovery rate

We will follow the convention in Bayesian fine mapping literature [27] to summarize the posterior distribution of the effect sizes $\beta_{jl}$ using marginal posterior inclusion probability (PIP) of each gene. Suppose we test a total of $p=\sum_{t=\text{1}}^{T}p_{t}$ genes across T genomic regions. We then perform an omnibus test of gene effects across all factors, $H_{\text{0}}:{⋂}_{l}\left\{\beta_{jl}=\text{0}\right\}$ vs. $H_{a}:{⋃}_{l}\left\{\beta_{jl}\neq \text{0}\right\}$, we define the gene-level PIP as $PIP_{j}:=P_{j}\left(H_{a}|data\right)=P({⋃}_{l}\left\{\beta_{jl}\neq \text{0}\right\}|data)$, 1≤j≤p. We then define the overall Bayesian false discovery rate (BFDR) [58] as: $BFDR(z)=\frac{\sum_{j=\text{1}}^{p}(\text{1}-PIP_{j})d_{j}(z)}{\sum_{j=\text{1}}^{p}d_{j}(z)}$, where $d_{j}(z)=I\{\text{1}-PIP_{j}<z\}$. In the two real-data application examples, we use this definition of the BFDR to select putative causal genes.

FM-GPT is implemented as an R package with computationally efficient C++ codes integrated via Rcpp. The package takes individual-level genotype (within a region of interest) and phenotype data as input and output prioritized putative causal genes along with their PIP, BFDR and the phenotypes factors they target at. The package is freely available at www.github.com/tacanida/fm-gpt.

### 5. Comparative methods

We compared our proposed method to several existing fine-mapping approaches. For TWAS fine-mapping methods, we primarily compared against GIFT [14], a frequentist method that focuses on conditional gene effects and is designed for single-trait analysis. GIFT can be implemented using either a one-stage or two-stage framework, with the two-stage GIFT being equivalent to MVIWAS [22]. We also attempted to implement FOCUS [15] and FOGS [16], but both methods were computationally infeasible and did not complete within a reasonable time. mvSuSIE [24] is an approximate Bayesian method that decomposes SNP effects into “single effects” to identify putatively causal SNPs, and it is the only fine-mapping method designed to simultaneously target multiple traits. PAINTOR [17] and CAVIAR [23] are frequentist methods primarily developed for GWAS fine-mapping that directly model SNP linkage disequilibrium (LD) to identify putatively causal variants. For single-trait methods (e.g., GIFT), we applied factor analysis first and then implemented the method using either single or multiple factors in simulations, or applied them separately to each trait in real data example 2. Detailed implementation procedures and parameter settings are provided in the S2 Method.

For Bayesian fine-mapping methods, including our own, we controlled for false discoveries by applying Bayesian false discovery rate (BFDR) procedures to select genes. For frequentist fine mapping methods that produce p-values, we used BH-adjusted [62] or BY-adjusted [63] p-values to control the FDR under weak or arbitrary dependence structures. Though Bayesian and frequentist FDR measures are not identical, BH- or BY-based FDR control procedures are among the most widely used multiple-testing criteria in high-dimensional genomic studies and are conceptually related to the BFDR criterion used by FM-GPT.

### 6. Description of database and cohort used in the real data application

#### The Genotype-Tissue Expression (GTEx) project.

The Genotype-Tissue Expression (GTEx) project [1] is a comprehensive resource designed to study the relationship between genetic variation and gene expression, by collecting postmortem tissue samples from over n_1_ = 800 deceased donors, covering approximately 50 distinct human tissue types. This extensive collection enables the characterization of tissue-specific regulatory effects of genetic variants, particularly expression quantitative trait loci (eQTLs), which are critical for understanding the genetic architecture of complex traits and diseases and have been widely used for TWAS [3]. For our analyses, we utilized the predicted gene expression weights generated by the GTEx Consortium (v8 release), available through the PredictDB repository (https://predictdb.org/post/2021/07/21/gtex-v8-models-on-eqtl-and-sqtl/). These pre-trained models cover multiple tissues and provide tissue-specific weights essential for TWAS.

#### UK Biobank (UKB).

The UK Biobank (UKB) is a large-scale prospective cohort study that recruited over 500,000 participants aged 37–73 years between 2006 and 2010 across 22 assessment centers throughout the UK ([64]). At baseline, participants provided extensive lifestyle, medical, and demographic information, as well as biological samples for genotyping, creating a rich resource for studying the genetic and environmental determinants of complex traits. For our study, UKB provides critical resources for two complementary applications. In the first example, structural brain MRI data collected for approximately ~ 30k participants starting in 2014 allows us to quantify cortical thickness measures across the whole brain. These high-resolution imaging phenotypes, combined with genetic data, enable the investigation of the genetic architecture of brain structure. In the second example, UKB’s extensive EHR data capture a wide range of disease diagnoses, treatments, and laboratory measurements across multiple organ systems. By linking these EHR-derived phenotypes with genotypes, we can perform phenome-wide analyses to identify putatively causal genes influencing multiple related but heterogeneous disease conditions.

## Supporting information

S1 Table a. Factor loading structure recovery comparison of FM-GPT with EFA and sPCA based on simulation scenario 1–2 (one true latent factor) with different outcome types and heritability levels. b. Number of factor recovery performance of FM-GPT in simulation scenario 1–2 (5% heritability) with varying number of true factors.
(XLSX)

S2 Table a. Simulation results based on scenario 1 (three true latent factors, heritability = 5%, mixed) with different proportions of binary traits. b. Simulation scenario 1 results (phenotype heritability 5% and true number of factors equal to 3, continuous) for different covariance matrix structures. c. Simulation scenario 1 results (true number of factors equal to 3) of FM-GPT when the number of factors are mis-specified [1,5,10]. d. Simulation scenario 1 results (true number of factors equal to 3, phenotypic heritability of 5%) of FM-GPT with varying proportion of binary cases.
(XLSX)

S3 Table UKB field ID and the brain region names for the CT measures in real data application 1. (XLSX)

S4 Table Univariate TWAS results for each regional CT measure in real data application 1. (XLSX)

S5 Table TWAS fine mapping results for regional CT measures in real data application 1. (XLSX)

S6 Table The estimated factor loadings for the regional CT measures in real data application 1. (XLSX)

S7 Table Pathway analysis results on the identified genes for the regional CT measures in real data application 1. (XLSX)

S8 Table The EHR phenotypes, categories belonging to and the data types in real data application 2. (XLSX)

S9 Table Univariate TWAS results for each EHR phenotype in real data application 2. (XLSX)

S10 Table TWAS fine mapping results for EHR phenotypes in real data application 2. (XLSX)

S11 Table The estimated factor loadings for EHR phenotypes in real data application 2. (XLSX)

S12 Table Pathway analysis results on the identified genes for EHR phenotypes in real data application 2. (XLSX)

S1 Fig Simulation I homogeneous results using reference LD + HAPGEN2 to generate genotype. (XLSX)

S2 Fig Simulation 2 heterogeneous results using reference LD + HAPGEN2 to generate genotype. (XLSX)

S3 Fig Traceplot of the number of factors in real data example 1. (XLSX)

S4 Fig Comparison of factor loadings from five random initialization runs or five random 90% subsampling runs with original results in real data example 2. (XLSX)

S5 Fig Phenotypic correlation matrix and scree plot of eigenvalues for real data example 1 (left) and 2 (right). (XLSX)

S1 Methods Generating genotype, gene expression and phenotype data in simulation. (PDF)

S2 Methods Implementation of comparative methods. (PDF)

S3 Methods Processing, classification and filtering of phenotypes from EHR data in UKB (real data example 2). (PDF)

S4 Methods Post-hoc unit-scaling and thresholding for factor loadings. (PDF)

S5 Methods Guidance for determining the number of factors. (PDF)

S6 Methods Gibbs sampler. (PDF)
