# Supplementary material for: FM-GPT: Bayesian fine mapping for phenome-wide transcriptome-wide association studies
Source: PLoS Genet. 2026 Sep 8;22(9):e1012126. doi: 10.1371/journal.pgen.1012126 (PMC13581217; doi:10.1371/journal.pgen.1012126)
Supplement: Supplementary file 1 [file pgen.1012126.s018.pdf]

S1. Generating genotype, gene expression and phenotype data in simulation

We have n_1=250 for the reference dataset and n_2 = 5000 for the GWAS dataset. We assume 10 independent regions, each containing 10 genes. Each gene includes 10 cis-SNPs, all of these cis-SNPs contribute to the GReX of the corresponding gene and explains 10% of the variation in gene expression, and the SNP sets were specified to be non-overlapping across genes, aligning well with the TWAS literature (1-3).

We considered two ways to generate LD structures and genotype data:

(1) Synthetic LD structure + genotype data generated from latent normal distribution: For each region, we assumed an LD matrix with correlation coefficient of 0.66 for cis-SNPs within the same gene, and an LD matrix with correlation coefficient of 0.33 for cis-SNPs in different genes. We then followed from(4, 5) to simulate the number of minor alleles for all SNPs (i.e. generate the genotype data) with fixed minor allele frequency (MAF) of 0.24 using latent Gaussian model with covariance matrix defined by the LD structure.

(2) Reference LD structure from 1000 genome + genotype data generated by HAPGEN2(6): For each region, we assumed an LD matrix directly obtained from the reference 1000 Genomes Project(7) to better reflect realistic population genetic structure. We first selected a region from Chromosome 15 and required to contain at least 10 protein-coding genes based on GENCODE v19 (3). We then extracted haplotypes from the 1000 Genomes Phase 3 IMPUTE2-formatted reference panel for chromosome 15. SNPs were retained if they fell within the region and had MAF $\geq$0.05 in the European subpopulation. Then, genotypes were simulated using HAPGEN2 (4) with the 1000 Genomes Phase 3 European haplotypes and the b37 chromosome 15 genetic map. Within the region, 10 protein-coding genes were selected by evenly sampling across TSS positions. For each gene, 10 causal cis-SNPs were randomly drawn from all MAF$\geq$0.05 SNPs within 500kbp of the TSS, yielding 10 gene-level simulation units in total. The noise variance was set so that the 10 causal SNPs together explained exactly 10% of the total expression variance (PVE = 0.10), matching the original design.

To generate the gene expression data for the reference dataset, we began by drawing the coefficients (i.e. SNP effect on gene) from a standard normal distribution (N(0,1)) for each SNP. The gene expression data was drawn from a normal distribution with mean being the SNP minor allele count matrix times the SNP coefficient vector and variance of 0.9. The above process was repeated for each gene.

Lastly, the phenotype data in GWAS was generated from the specified models by varying the heritability levels (1%, 3%, 5%) and the number of phenotypical factors (1, 3, 5) for each of the 50 traits. Each simulation was repeated for 100 times.

Reference:

1. Gamazon ER, Wheeler HE, Shah KP, Mozaffari SV, Aquino-Michaels K, Carroll RJ, et al. A gene-based association method for mapping traits using reference transcriptome data. Nat Genet. 2015;47(9):1091-8.
2. Gusev A, Ko A, Shi H, Bhatia G, Chung W, Penninx BW, et al. Integrative approaches for large-scale transcriptome-wide association studies. Nat Genet. 2016;48(3):245-52.
3. Barbeira AN, Dickinson SP, Bonazzola R, Zheng J, Wheeler HE, Torres JM, et al. Exploring the phenotypic consequences of tissue specific gene expression variation inferred from GWAS summary statistics. Nat Commun. 2018;9(1):1825.
4. Wang T, Zhu X, Elston RC. Improving power in contrasting linkage-disequilibrium patterns between cases and controls. The American Journal of Human Genetics. 2007;80(5):911-20.
5. Han F, Pan W. A composite likelihood approach to latent multivariate Gaussian modeling of SNP data with application to genetic association testing. Biometrics. 2012;68(1):307-15.
6. Su Z, Marchini J, Donnelly P. HAPGEN2: simulation of multiple disease SNPs. Bioinformatics. 2011;27(16):2304-5.
7. Auton A, Brooks LD, Durbin RM, Garrison EP, Kang HM, Korbel JO, et al. A global reference for human genetic variation. Nature. 2015;526(7571):68-74.
