# Supplementary material for: FM-GPT: Bayesian fine mapping for phenome-wide transcriptome-wide association studies
Source: PLoS Genet. 2026 Sep 8;22(9):e1012126. doi: 10.1371/journal.pgen.1012126 (PMC13581217; doi:10.1371/journal.pgen.1012126)
Supplement: Supplementary file 2 [file pgen.1012126.s019.pdf]

S2. Implementation of comparative methods

GIFT Factor (one-stage)(1):

For each genomic region to fine map, we constructed the required inputs for GIFT as follows. We first estimated tissue-specific genetically regulated expression (GReX) via PrediXcan. GWAS summary statistics for the phenotypes of interest were restricted to variants within the region and converted to z-scores ($Zscore1$). LD matrices were computed using study genotypes (LDmatrix1) via PLINK and from the 1000 Genomes European reference panel (LDmatrix2). eQTL z-scores ($Zscore2$) were derived from prediction weights scaled by their variances. We also estimated the gene-gene correlation matrix (R) using predicted gene expression. Lastly, we determined the number of genetic variants mapped to each gene denoted as pindex. Because a single variant may regulate multiple genes, we expanded the Zscore1 and Zcore2 by duplicating variants so that each variant–gene pair was represented as a separate entry. We also ensured that the variant ordering was consistent between the Zscore1 and Zscore2 matrices. This step ensured that variants associated with multiple genes were handled appropriately without double counting when calculating the number of variants contributing to each gene. These components, Zscore1, Zscore2, LDmatrix1, LDmatrix2, pindex, and R, were used as inputs for the one-stage GIFT framework to perform conditional TWAS fine-mapping within each genomic region using the GIFT_summary function. Analyses were conducted using the default parameter settings, including a maximum of 100 iterations, a convergence tolerance of 1×10^{-3}, no pleiotropy adjustment, single-core computation, and a conditional gene window size of five.

GIFT (two-stage)/MVIWAS Factor(2):

Instead of the joint likelihood framework implemented in the one-stage GIFT, we also implemented the two-stage GIFT approach (equivalent to MVIWAS) separately for each tissue and genomic region, which performs sequential analyses consisting of gene expression prediction followed by regression of GWAS effects on the predicted gene expression effects. Under this framework, the required inputs include the tissue-specific weight matrix derived from GTEx, the GWAS effect sizes and their corresponding standard errors, the LD matrix of the genetic variants estimated from the study subjects, and the GWAS sample size. Analyses were also conducted using their default parameter settings.

GIFT Meta (one-stage)(1):

In the simulation settings, we additionally compare the performance of GIFT (one-stage) when it is applied to each simulated phenotype individually. To do so, we first estimate the weight matrix as for GIFT Factor. Then, for each phenotype, we compute a marginal linear model SNP-by-SNP for all SNPs in the genomic region and extract its regression beta value as well as its standard error and convert the regression coefficients to their z-values. Then, for each phenotype, we use its set of z-values associated with each SNP and compute the p-values for the associations

between the set of genes in the region against the individual phenotype. After this has been done for all simulated phenotypes, the p-values for each gene as aggregated using Fisher's method and are then used for downstream analysis of AUC, FDR and power.

GIFT Meta (two-stage)/MVIWAS Meta(1):

We also implement the MVIWAS method per-phenotype with p-value aggregation. To do so, we first estimate the SNP-gene weight matrix. Then, for each simulated phenotype, we apply a marginal linear regression SNP-by-SNP for each SNP in the simulated region and calculate its regression coefficient beta as well as its associated standard error. We then use these as inputs to the MVIWAS function. For each phenotype, we get a p-value for test of association between each gene and that phenotype. Once this is done for all phenotypes, we combine the set of p-values for each gene using Fisher's method, and use these aggregated p-values for downstream analysis of AUC, FDR and power.

mvSuSIE(3):

Since mvSuSIE is a multivariate method for continuous outcome variables, we ran it on the covariate adjusted using the following steps. First, we performed a regular linear model, regressing each of the brain cortical thickness measurements onto the set of covariates. We then adjusted the outcome matrix by keeping the residual from subtracting the predicted values from the covariate linear model. We then ran mvSuSIE on the entire set of covariate adjusted brain region cortical thickness measurements as a multivariate model. We centered each the matrix of predicted gene expressions and covariate adjusted cortical thickness measurements before running their model. For the analysis of variable selection and calculation of the BFDR, we used their overall PIP.

PAINTOR(4):

To compare against PAINTOR in the simulation settings, we begin by specifying the input LD matrix as the LD matrix generated as specified in S1. Then, using the one factor representation of the generated continuous or mixed outcomes, we compute the marginal Z-score for each SNP in the simulated GWAS dataset. Since PAINTOR accepts additional annotation information, we set the annotation to zero for each SNP. Finally, we run PAINTOR on each simulated region by specifying the enumerate to 2. This aligns well with our causal gene and heritability setting. When determining whether a gene was selected by PAINTOR, we examine the posterior inclusion probabilities for the SNPs, and choose the highest of these to represent the PIP of the gene.

CAVIAR(5):

Similarly to PAINTOR, we begin by specifying the input LD matrix as that generated as specified in S1. We then utilize the one factor representation of the generated continuous or mixed data outcomes and compute the marginal Z-score for each SNP in the generated GWAS dataset. Since CAVIAR also accepts additional annotation information, we set it to zero for each SNP. Then, we run CAVIAR on each region and specify the number of causal variants to 2. In determining whether or not a gene is chosen, we follow a similar decision as in PAINTOR and examine the posterior inclusion probability for each cis-SNP for each gene. We choose the highest PIP of the cis-SNPs for a gene to be the PIP for that gene.

S3. Processing, Classification and Filtering of Phenotypes from EHR data in UKB (real data example 2)

We processed 19190 ICD-10 codes from EHR data and converted into 1403 phenotypes primarily following the protocol in Pheweb(6): For each of the 1403 phenotypes, we manually searched the set of 19190 ICD-10 codes to select the ones that make up that phenotype based on their names. For example, if the phenotype is rheumatoid arthritis, we selected all instances of rheumatoid arthritis throughout the entire body for the subject. For phenotypes with multiple ICD-10 codes, the total amount of incidences are combined to create either a count or a binary phenotype. Phenotypes with only one ICD-10 code are considered binary. These phenotypes are then classified into 16 broad categories: musculoskeletal, digestive, circulatory, dermatologic, endocrine/metabolic, neoplasms, mental disorders, respiratory, sense organs, injuries & poisonings, genitourinary, hematopoietic, symptoms, infectious diseases, neurological and congenital anomalies. For binary phenotypes, we further performed filtering based on case counts and kept only phenotypes with at least 1% of cases. For count data based phenotypes, we kept the phenotypes when they have at least one count. After filtering, we kept 169 phenotypes across 15 categories.

Reference:

1. Liu L, Yan R, Guo P, Ji J, Gong W, Xue F, et al. Conditional transcriptome-wide association study for fine-mapping candidate causal genes. Nature Genetics. 2024;56(2):348-56.
2. Knutson KA, Deng Y, Pan W. Implicating causal brain imaging endophenotypes in Alzheimer's disease using multivariable IWAS and GWAS summary data. NeuroImage. 2020;223:117347.
3. Zou Y, Carbonetto P, Xie D, Wang G, Stephens M. Fast and flexible joint fine-mapping of multiple traits via the Sum of Single Effects model. bioRxiv. 2023:2023.04.14.536893.
4. Kichaev G, Yang W-Y, Lindstrom S, Hormozdiari F, Eskin E, Price AL, et al. Integrating functional data to prioritize causal variants in statistical fine-mapping studies. PLoS genetics. 2014;10(10):e1004722.
5. Hormozdiari F, van de Bunt M, Segrè AV, Li X, Joo JWJ, Bilow M, et al. Colocalization of GWAS and eQTL Signals Detects Target Genes. Am J Hum Genet. 2016;99(6):1245-60.
6. Zhou W, Nielsen JB, Fritsche LG, Dey R, Gabrielsen ME, Wolford BN, et al. Efficiently controlling for case-control imbalance and sample relatedness in large-scale genetic association studies. Nat Genet. 2018;50(9):1335-41.
