# Supplementary material for: FM-GPT: Bayesian fine mapping for phenome-wide transcriptome-wide association studies
Source: PLoS Genet. 2026 Sep 8;22(9):e1012126. doi: 10.1371/journal.pgen.1012126 (PMC13581217; doi:10.1371/journal.pgen.1012126)
Supplement: Supplementary file 3 [file pgen.1012126.s020.pdf]

S3. Processing, Classification and Filtering of Phenotypes from EHR data in UKB (real data example 2)

We processed 19190 ICD-10 codes from EHR data and converted into 1403 phenotypes primarily following the protocol in Pheweb(13): For each of the 1403 phenotypes, we manually searched the set of 19190 ICD-10 codes to select the ones that make up that phenotype based on their names. For example, if the phenotype is rheumatoid arthritis, we selected all instances of rheumatoid arthritis throughout the entire body for the subject. For phenotypes with multiple ICD-10 codes, the total amount of incidences are combined to create either a count or a binary phenotype. Phenotypes with only one ICD-10 code are considered binary. These phenotypes are then classified into 16 broad categories: musculoskeletal, digestive, circulatory, dermatologic, endocrine/metabolic, neoplasms, mental disorders, respiratory, sense organs, injuries & poisonings, genitourinary, hematopoietic, symptoms, infectious diseases, neurological and congenital anomalies. For binary phenotypes, we further performed filtering based on case counts and kept only phenotypes with at least 1% of cases. For count data based phenotypes, we kept the phenotypes when they have at least one count. After filtering, we kept 169 phenotypes across 15 categories.
