# Supplementary material for: FM-GPT: Bayesian fine mapping for phenome-wide transcriptome-wide association studies
Source: PLoS Genet. 2026 Sep 8;22(9):e1012126. doi: 10.1371/journal.pgen.1012126 (PMC13581217; doi:10.1371/journal.pgen.1012126)
Supplement: Supplementary file 4 [file pgen.1012126.s021.pdf]

S4. Post-hoc unit-scaling and thresholding for factor loadings

In traditional exploratory factor analysis (EFA), when both the observed variables and latent factors are standardized, the loadings can be interpreted as the correlation between outcome and latent factors, i.e. $\lambda_{kl} = \mathrm{corr}(y_k, f_l)$. Under this convention, loadings are restricted to the range [-1,1], which facilitates interpretation and allows small loadings (e.g. <0.1) to be thresholded to exact zeros for clearer visualization of the loading matrix. In our formulation of the Bayesian factor analysis model, this constraint is not imposed, so the estimated loadings may fall outside [-1,1]. Although this unconstrained parameterization is computationally convenient, it makes the magnitude of the loadings less directly comparable across factors and complicates the choice of a threshold for suppressing negligible loadings.

To help improve the interpretability of factor loadings in our model, we propose a post-hoc unit scaling technique. For each iteration of the Gibbs sampler, b, we unit scale each of the loadings $\lambda_{b,kl}$ as:

$$\lambda^*_{b,kl} = \frac{\lambda_{b,kl}}{\sqrt{\sum_k \lambda^2_{b,kl}}}$$

After this normalization, $\lambda^*_{b,kl}$ represents the relative contribution of each outcome to factor. We then summarize the posterior median of the unit-scaled loadings as the final estimates for a more fair comparison with the conventional factor analysis methods, allowing small loadings (e.g. <0.1) to be thresholded to exact zeros for clearer visualization of the loading matrix as in EFA.
