# Supplementary material for: FM-GPT: Bayesian fine mapping for phenome-wide transcriptome-wide association studies
Source: PLoS Genet. 2026 Sep 8;22(9):e1012126. doi: 10.1371/journal.pgen.1012126 (PMC13581217; doi:10.1371/journal.pgen.1012126)
Supplement: Supplementary file 5 [file pgen.1012126.s022.pdf]

**S5. Guidance for Determining the Number of Factors**

FM-GPT includes an adaptation-within-Gibbs step that allows the effective number of factors to be learned from the data. This adaptive procedure is controlled by a tuning parameter, $\varepsilon$, which defines the minimum loading magnitude required for a factor to be retained. Larger value of $\varepsilon$ lead to more aggressive factor pruning and therefore fewer retained factors. By default, a factor is retained only if e.g., 50% of the loadings in absolute value for a factor are at least $\varepsilon$ (e.g. 1e-4). Additionally, FM-GPT provides several diagnostics (included in the tutorial) to help users assess the appropriate number of factors. First, users can examine the trace plot of the number of factors retained across Gibbs sampling iterations and evaluate the convergence. Second, users can also inspect the phenotype correlation matrix to have some idea of how many factors exist in the latent structure and then examine the eigenvalues of the phenotype correlation matrix in a scree plot, and identify an elbow point for a reasonable number of factor, as in other factor or clustering analysis methods. Because FM-GPT is applied separately to each genomic region, the effective number of factors may differ across regions. Thus, the phenotypic correlation matrix and scree plot should be used as exploratory guidance rather than definitive criteria; we recommend combining them with region-specific trace plots and loading-matrix diagnostics to select the number of factors. In addition, FM-GPT allows users to visualize the posterior mean of the loading matrix. This visualization can help identify factors whose loadings are uniformly small relative to those of other factors, thus can be excluded.

We provide example diagnostic plots of determining the number of factors in the two real data examples below (correlation plots and scree plots of the phenotypes for real data examples 1 and 2):

Figure S5. Phenotypic correlation matrix and scree plot of eigenvalues for real data example 1 (left) and 2 (right).

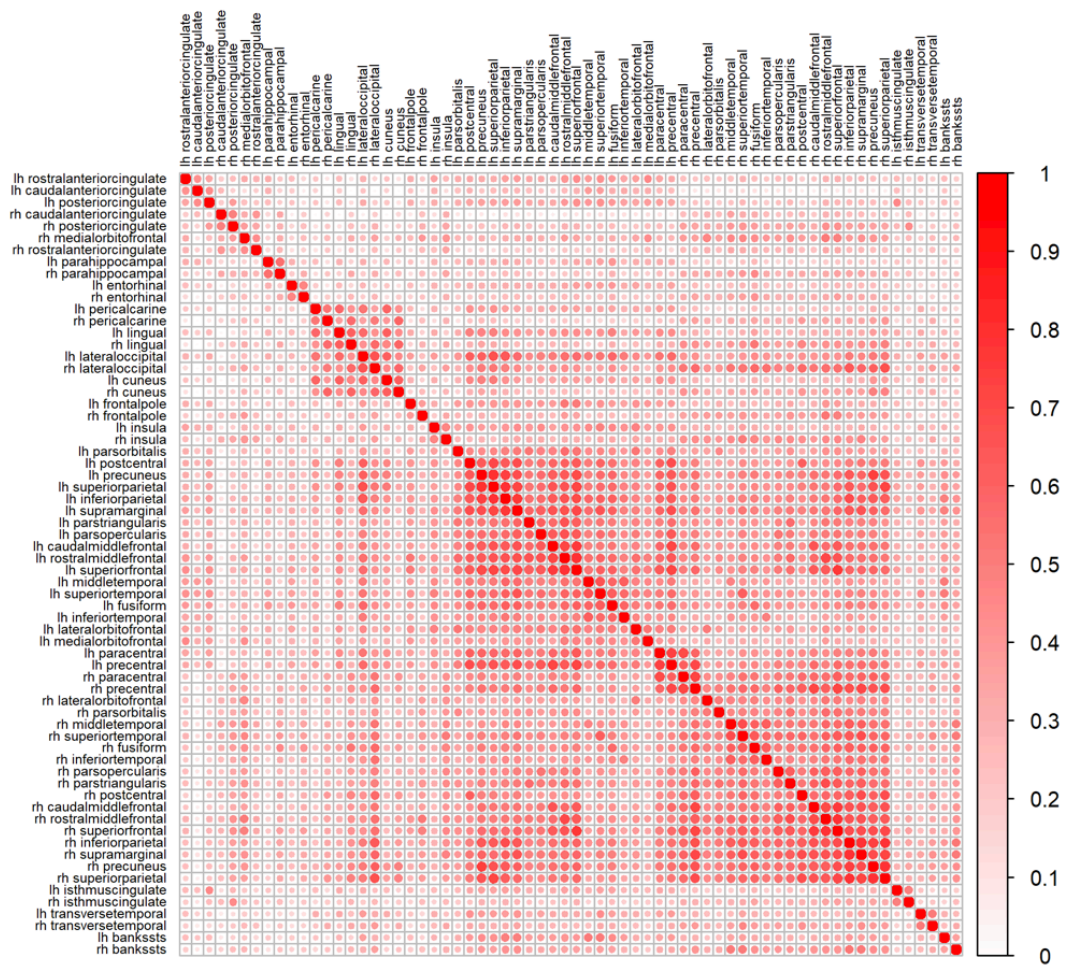

Real Data Example 1

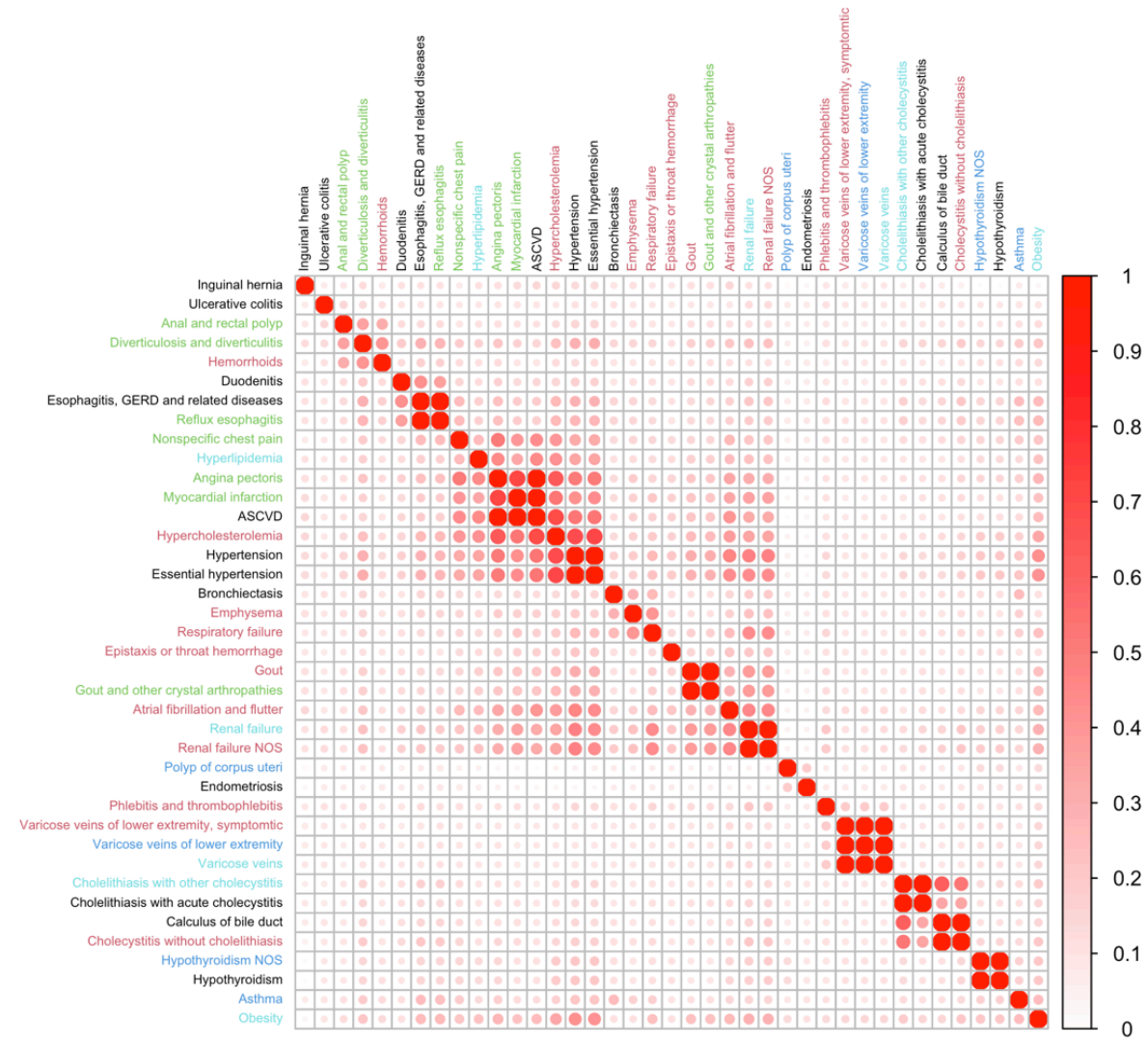

Real Data Example 2

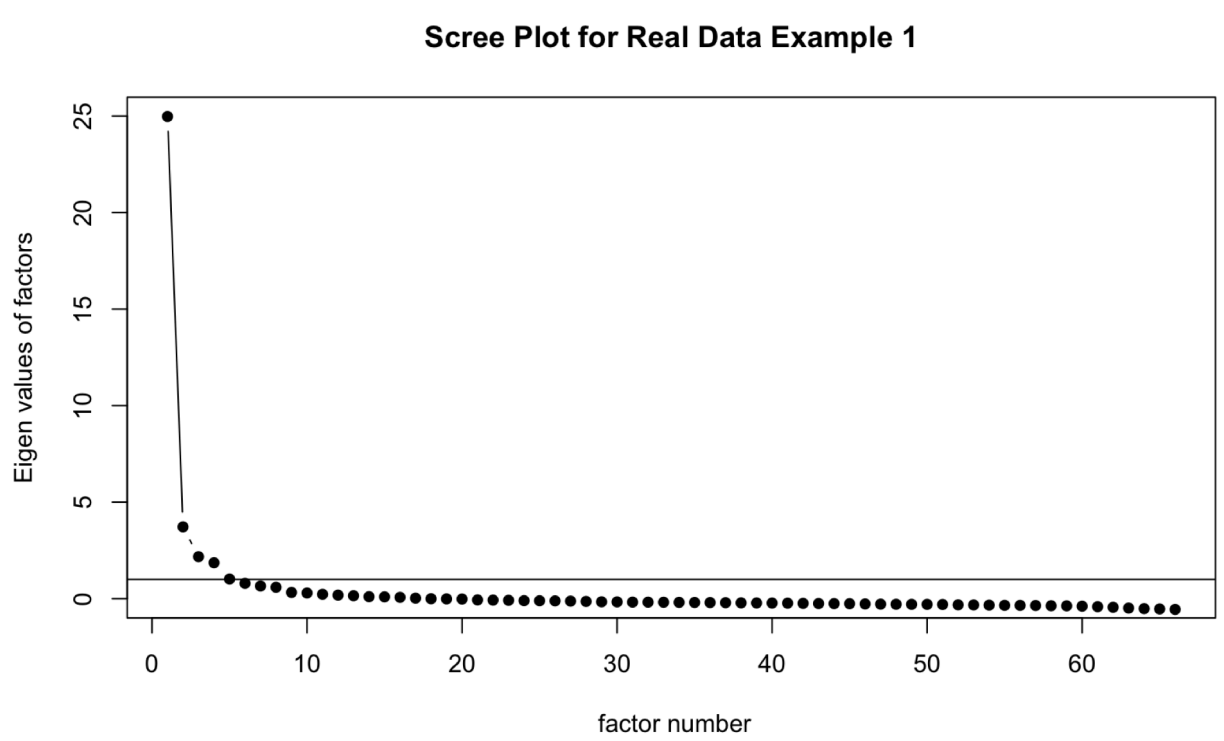


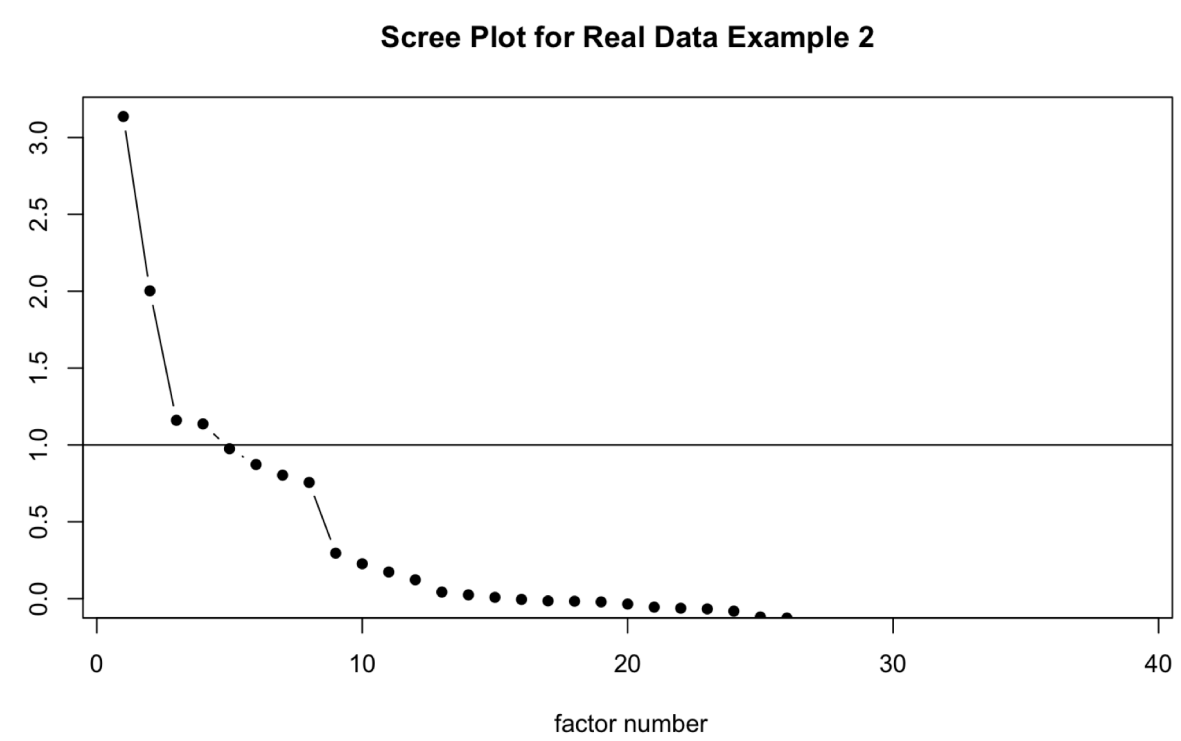


For real data example 1, inspection of the phenotype correlation matrix and the corresponding scree plot suggested a low-dimensional latent structure, with one or two factors being plausible (FigS5 left). We further examined the trace plot of the number of factors retained during the adaptive Gibbs sampler. The trace plot (below) for a selected region showed that FM-GPT primarily fluctuated between one and two factors. This pattern occurred because the adaptation step occasionally added a second factor when the existing factor satisfied the retention criterion, but the additional factor was subsequently removed because its loadings were weak and did not exceed the threshold. We therefore interpreted the second factor as unstable and weakly supported by the data. Because the first factor captured the dominant latent structure and the second factor did not show a consistent or interpretable loading pattern, we selected a one-factor model for real data example 1.

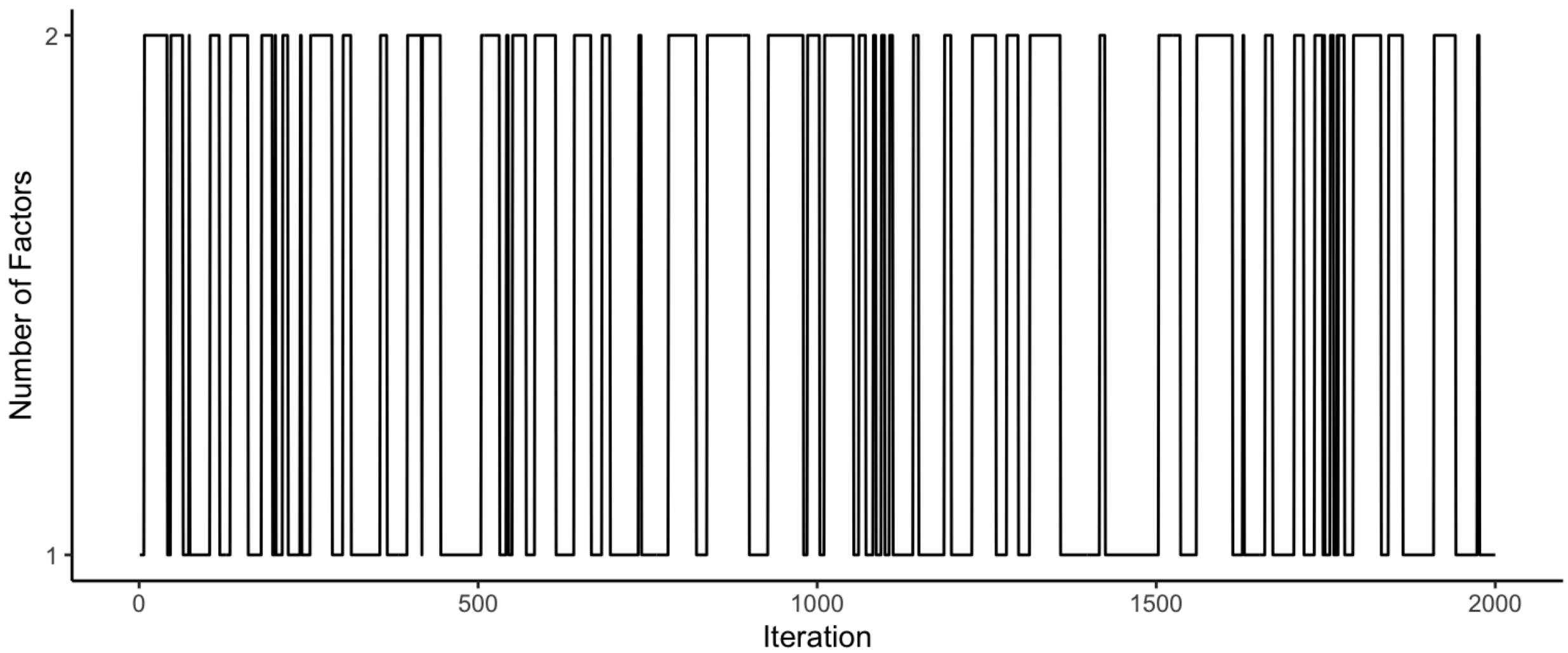


For real data example 2, we selected a three-factor model based on exploratory diagnostics of the phenotype correlation structure (Fig S5 right). Visual inspection of the phenotype correlation matrix suggested the presence of multiple correlated phenotype clusters, consistent with a low-rank latent structure involving more than one factor. The scree plot further supported this choice: the eigenvalue pattern showed a clear elbow at three factors, and all subsequent eigenvalues were less than one. Together, these diagnostics indicated that the first three factors captured the dominant shared variation among the phenotypes, whereas additional factors were unlikely to explain substantial residual correlation structure.
