# Supplementary material for: FM-GPT: Bayesian fine mapping for phenome-wide transcriptome-wide association studies
Source: PLoS Genet. 2026 Sep 8;22(9):e1012126. doi: 10.1371/journal.pgen.1012126 (PMC13581217; doi:10.1371/journal.pgen.1012126)
Supplement: Supplementary file 6 [file pgen.1012126.s023.pdf]

S6. Gibbs Sampler

In our model, the primary parameters of interest are $\mathbf{F}$, the matrix of latent factors, $\mathbf{\Lambda}$, the loading matrix which relates the latent factor matrix to the observed phenotypes and $\mathbf{B}$, the matrix containing the coefficients describing how the gene expression affects the latent factors. For example, $\mathbf{b}_{jl}$ is the effect of gene j on factor l. At each iteration t, we use the following formulas to update the Gibbs sampler. Firstly, we expand Equation (3) to $\mathbf{\Lambda F}_i + \mathbf{u}_i + \mathbf{E}_i$ and let $\mathbf{u}_i \sim N(\mathbf{0}, \mathbf{\Sigma})$ and $\mathbf{E}_i \sim N(\mathbf{0}$, diag$(\psi_{i1}, \ldots, \psi_{iq}))$. This allows the random effect vector $\mathbf{u}_i$ to capture the covariance among the outcome variables, and the error vector $\mathbf{E}_i$ to capture the augmented data vector $\boldsymbol{\psi}_i$. Then we have the following.

Updating steps for Equation (1):

1. Updating γ_j

   We first draw the entire set γ_j from a multivariate normal distribution as: $\gamma_j|\text{rest} \sim N((\frac{1}{\sigma_j^2}(Z_j^R)'Z_j^R + \frac{1}{\sigma_j^2\upsilon_j^2}\mathbf{I})^{-1}(Z_j^R)'x_j\frac{1}{\sigma_j^2}, \frac{1}{\sigma_j^2}(Z_j^R)'Z_j^R + \frac{1}{\sigma_j^2\upsilon_j^2}\mathbf{I})^{-1})$ Then, if $I_{oj}^{(\gamma)} = 0$, we set that γ_{oj} to zero.

2. Updating t_j^2:

   $\upsilon_j^2|\text{rest} \sim \text{IG}(\frac{\sum I_d^{(\gamma)}}{2}, \frac{\gamma_j'\gamma_j}{2\sigma_j^2})$

3. Updating σ_j^2:

   $\sigma_j^2|\text{rest} \sim \text{IG}(\frac{n_1}{2}, \frac{(x_j - Z_j^R\gamma_j)'(x_j - Z_j^R\gamma_j)}{2})$

4. Updating $\pi^{(\gamma)}$

   $\pi_j^{(\gamma)}|\text{rest} \sim \text{Beta}(1 + \sum I_d^{(\gamma)}, 1 + \sum(1 - I_d^{(\gamma)}))$

5. Updating $I_d^{(\gamma)}$

   $I_d^{(\gamma)}|\text{rest} \sim \text{Bern}(\pi_*^{(\gamma)})$

   $$\pi_*^{(\gamma)} = 1 - \frac{1-\pi^{(\gamma)}}{\pi^{(\gamma)}(\sigma_j^2 t_j^2)^{-\frac{1}{2}} \exp\left(\frac{(Z_j^R z_{-d})^2}{2\sigma_j^2((Z_j^R)' Z_j^R + \frac{1}{t_j^2})}\right)\left(\frac{\sigma_j^2}{(Z_j^R)' Z_j^R + \frac{1}{t_j^2}}\right)^{\frac{1}{2}} + (1-\pi^{(\gamma)})}$$

   Where $z_{-d} = x_j - Z_{j,-d}^R \gamma_{j,-d}$ removes the column for the dth cis SNP.

   Updating steps for Equation (2):

6. Updating $\mathbf{b}_j$

   The conditional posterior distribution of $\mathbf{b}_j$ is a multivariate normal distribution. Let M_j b an m element vector with the lth element $M_{jl} = I_j U_{jl}(\mathbf{X}_j' \mathbf{X}_j + \frac{1}{s^2})^{-1} \mathbf{X}_j' \tilde{\mathbf{Y}}_{(j)k}$ where $\mathbf{X}_j$ is the jth column of $\mathbf{X}$, $\tilde{\mathbf{Y}}_{(j)} = \mathbf{Y} - \mathbf{X}_{(j)} \mathbf{B}_{(j)}$, where $\mathbf{X}_{(j)}$ is the $\mathbf{X}$ matrix without the jth column and $\mathbf{B}_{(j)}$ is the $\mathbf{B}$ matrix without the jth row. Let Ξ_j be the covariance matrix with diagonal elements $\Xi_{j,ll} = (\frac{1}{s^2}(\mathbf{X}_j' \mathbf{X}_j + \frac{1}{s^2})^{-1})^{I_j U_{jl}} s^2 \Xi_{ll}$ and off diagonal elements $\Xi_{j,ll'} = (\frac{1}{s^2}(\mathbf{X}_j' \mathbf{X}_j + \frac{1}{s^2})^{-1})^{\frac{1}{2}(I_j U_{jl} + I_j U_{jl'})} s^2 \Xi_{ll'}$ where $l \neq l'$. Then, we have that:

   $\mathbf{b}_j|\text{rest} \sim N(M_j, \Xi_j)$

7. Updating s^2

   $s^2|\text{rest} \sim \text{IG}(\frac{pm}{2}, \frac{1}{2}\text{Tr}(\mathbf{B}\Sigma^{-1}\mathbf{B}')$

8. Updating $\mathbf{\Omega}$

   $\mathbf{\Omega} \sim \text{IW}(\sum_{j=1}^{p} \sum_{l=1}^{m} I_j U_{jl} + m + n_2, \mathbf{I}_m + (\mathbf{F} - \mathbf{XB})'(\mathbf{F} - \mathbf{XB}) + \frac{1}{s^2}\mathbf{B}'\mathbf{B})$

9. Updating π_1^β and π_2^β

   The conditional posterior distributions of the parameters π_1^β and π_2^β are beta distribution:

   $$\pi_1^\beta|\text{rest} \sim \text{Beta}(1 + \sum_{j=1}^{p} I_j^{(\beta)}, 1 + \sum_{j=1}^{p}(1 - I_j^{(\beta)}))$$
   $$\pi_2^\beta|\text{rest} \sim \text{Beta}(1 + \sum_{l=1}^{m} U_{jl}^{(\beta)}, 1 + \sum_{l=1}^{m}(1 - U_{jl}^{(\beta)}))$$

10. Updating $I_j^{(\beta)}$

$I_j^{(\beta)}|\text{rest} \sim \text{Bern}(\pi_{1*}^{(\beta)})$

$\pi_{1*}^{(\beta)} = \frac{\pi_1^{(\beta)}\exp\left\{-\frac{1}{2}\text{Tr}\left[\mathbf{X}_j\mathbf{B}_j\Omega^{-1}\mathbf{B}_j^T\mathbf{X}_j^T - 2\mathbf{Y}_{(j)}\Omega^{-1}\mathbf{B}_j^T\mathbf{X}_g^T\right]\right\}}{\pi_1^{(\beta)}\exp\left\{-\frac{1}{2}\text{Tr}\left[\mathbf{X}_g\mathbf{B}_j\Omega^{-1}\mathbf{B}_j^T\mathbf{X}_j^T - 2\mathbf{Y}_{(j)}\Omega^{-1}\mathbf{B}_j^T\mathbf{X}_j^T\right]\right\} + (1-\pi_1^{(\beta)})}$, $\mathbf{X}_g$ and $\mathbf{B}_j$ refer to the jth column of $\mathbf{X}$ and jth row of $\mathbf{B}$, respectively.

11. Updating $U_{jl}^{(\beta)}$

$U_{jl}^{(\beta)}|\text{rest} \sim \text{Bern}(\pi_{2*}^{(\beta)})$

$\pi_{2*}^{(\beta)} = \frac{\pi_2^{(\beta)}\exp\left\{-\frac{1}{2}\text{Tr}\left[\mathbf{X}_{jl}\beta_{jl}\Omega^{-1}\beta_{jl}^T\mathbf{X}_{jl}^T - 2\mathbf{Y}_{(jl)}\Omega^{-1}\beta_{jl}^T\mathbf{X}_{jl}^T\right]\right\}}{\pi_2^{(\beta)}\exp\left\{-\frac{1}{2}\text{Tr}\left[\mathbf{X}_{jl}\beta_{jl}\Omega^{-1}\beta_{jl}^T\mathbf{X}_{jl}^T - 2\mathbf{Y}_{(jl)}\Omega^{-1}\beta_{jl}^T\mathbf{X}_{jl}^T\right]\right\} + (1-\pi_2^{(\beta)})}$

Updating steps for Equation (3):

12. Updating $\mathbf{f}_i$

Let

$\mathbf{V} = (\mathbf{\Lambda}\mathbf{\Psi}_i^{-1}\mathbf{\Lambda}' + \mathbf{\Omega}^{-1})^{-1}$ and

$\mathbf{M} = \mathbf{V}(\mathbf{\Lambda}\mathbf{\Psi}_i^{-1}(\mathbf{\Xi}_i - \mathbf{u}_i) + \mathbf{\Omega}^{-1}\mathbf{B}'\mathbf{X}_i)$

Then, $\mathbf{f}_i|\text{rest} \sim MVN(\mathbf{M}, \mathbf{V})$ for $1 \le i \le n_2$

13. Updating $\boldsymbol{\lambda}_k$

Let $\mathbf{D}_k^{-1} = \text{diag}(\phi_{k1}\tau_1, \ldots, \phi_{km}\tau_m)$

Then, $\boldsymbol{\lambda}_k|\text{rest} \sim N((\mathbf{D}_k^{-1} + \mathbf{F}'\mathbf{\Psi}_k\mathbf{F})^{-1}(\mathbf{F}'\mathbf{\Psi}_k(\mathbf{\Xi}_k - \mathbf{u}_k)), (\mathbf{D}_k^{-1} + \mathbf{F}'\mathbf{\Psi}_k\mathbf{F})^{-1})$

14. Updating $\mathbf{u}_i$

$\mathbf{u}_i|\text{rest} \sim MVN((\mathbf{\Psi}_i^{-1}\mathbf{\Sigma}^{-1})^{-1}(\mathbf{\Psi}_i^{-1}(\mathbf{\Xi}_i - \mathbf{\Lambda}'\mathbf{F}_i)), (\mathbf{\Psi}^{-1}\mathbf{\Sigma}^{-1})^{-1})$

15. Updating ϕ_{kl}

$\phi_{kl}|\text{rest} \sim \text{Ga}(\frac{4}{2}, \frac{3+\tau_k\lambda_{kl}}{2})$

16. Updating ϑ_1

$\vartheta_1|\text{rest} \sim \text{Ga}\left(a_1 + \frac{qm}{2}, 1 + \frac{\sum_{l=1}^m \tau_l^{(1)} \sum_{k=1}^q \phi_{kl}\lambda_{kl}^2}{2}\right)$

17. Updating ϑ_h

    $\vartheta_h|\text{rest} \sim \text{Ga}\left(a_2 + \frac{q}{2}(m-h+1), 1 + \frac{\sum_{l=1}^{m} \tau_l^{(h)} \sum_{k=1}^{q} \phi_{kl}\lambda_{kl}^2}{2}\right)$

18. Updating Σ_{kk}^{-1}

    $\Sigma_{kk}^{-1}|\text{rest} \sim \text{Ga}(a_\sigma + \frac{n_2}{2}, b_\sigma + \frac{1}{2}\sum_{i=1}^{n_2} u_{ik}^2)$

19. Updating a_1 and a_2

    Update using a Metropolis-Hastings within the Gibbs sampler

20. Updating ψ_{ik}

    $\psi_{ik} \sim \text{Polya-Gamma}(\xi_{ik}, \mathbf{F\Lambda} + \mathbf{u})$

    Where $\xi_{ik} = 1$ if y_{ik} is binary and $y_{ik} + r_k$ if y_{ik} is count.

21. Updating r_k

    First, assuming the kth column of $\mathbf{Y}$ is count, then for each observed count y_{ik}, we introduce a sample of latent counts l_i, such that, conditional on y_{ik} and r_k, l_i follows a Chinese restaurant table distribution:

    $$l_i = \sum_{j=1}^{y_{ik}} u_j$$
    $$u_j \sim \text{Bern}(\frac{r_k}{r+j-1})$$

    In step 1, we draw each l_i from this distribution, then in step 2, since the negative binomial distribution can be derived from a random convoluation of logarithmic random variables, we have that, conditional on r_k and $\chi_{ik} = \frac{\exp \mathbf{F}_i\mathbf{\Lambda}}{1+\exp \mathbf{F}_i\mathbf{\Lambda}}$,

    $$l_i \sim \text{Pois}(-r_k \log 1 - \chi_i)$$

    Then, assuming a gamma prior on r_k, the full conditional for r_k in step 2 is:

    $$r_k|\text{rest} \sim \text{Gamma}(c_1 + \sum_i l_i, c_2 - \log(1-\chi_x))$$

22. Updating the number of factors:

    To update the number of factors, at each iteration t, we propose a probability of updating:

$$p(t) = \exp(-1 - 0.0005t)$$

    and draw $u(t) \sim \text{Unif}(0, 1)$. If $u(t) \leq p(t)$, then we update the number of factors by checking if any loading vector has any element within a pre-specified small neighborhood of zero. We remove any such factors with loadings satisfying this requirement. If the total number of columns satisfying this requirement is zero, we add an additional factor.
