# Supplementary material for: FM-GPT: Bayesian fine mapping for phenome-wide transcriptome-wide association studies
Source: PLoS Genet. 2026 Sep 8;22(9):e1012126. doi: 10.1371/journal.pgen.1012126 (PMC13581217; doi:10.1371/journal.pgen.1012126)
Supplement: Supplementary file 7 [file pgen.1012126.s025.docx]

**Response Letter**

We want to thank all reviewers for taking time to review our manuscript and provide valuable comments and suggestions to improve the quality of our manuscript. We have taken the advice from reviewers to carefully revise the manuscript (revised parts red highlighted). Below please also find a point-by-point response to reviewers' comments:

**Reviewer #1**: The authors proposed a new TWAS fine mapping method for multiple traits of possibly mixed types with a fully Bayesian approach. Numerical studies with both simulated and UKB data show both the feasibility and some advantages of the proposed method over several existing methods.

*Major comments.*

1. A main promise of the proposed method is its capability to handle multiple types of traits, for which the major technical approach is a normal distribution-based latent factor analysis of the multiple traits to be analyzed. The following questions need to be clarified.

1) For a discrete trait, how/why is the proposed normal-based factor analysis reasonable or justified? To be concrete, let’s consider a binary trait (among multiple traits): then the corresponding LHS of eq (3) is a binary random variable (RV) (after subtracting a constant, then divided by another random variable), but the RHD is a normal random variable; how can such a linear transformation is possible (to transform a binary RV to a normal RV)?

Response:

Thank you for your comment. We have revised the text in Methods, Section 1 and 3 to clarify the interpretation of equations (3) and (4). In the revised manuscript, equation (4) defines the augmented response used in the data augmentation scheme, whereas equation (3) links this augmented response to the continuous latent factors through the factor model. Importantly, equation (4) should not be interpreted as a deterministic transformation of an observed discrete random variable into a Gaussian random variable. Instead, for discrete outcomes, we introduce a Pólya–Gamma auxiliary variable ($\psi$). Conditional on $\psi$, the likelihood contribution for the natural parameter $\theta$ (i.e. $\Lambda F$ in equation (3)) is proportional to a Gaussian kernel. Although this pseudo-response is not itself assumed to be marginally Gaussian, conditional on the Pólya–Gamma auxiliary variable, the likelihood contribution for the natural parameter $\theta$ has a Gaussian kernel. This conditional Gaussian representation allows continuous and discrete outcomes to be handled within a unified latent-factor framework while preserving the original discrete likelihood after integrating over the auxiliary variable. Thus, equation (3) should be understood as a conditionally Gaussian representation of the augmented likelihood, where the left-hand side denotes either the observed continuous response or the augmented continuous pseudo-response induced by the Pólya–Gamma data augmentation scheme. Additional technical details are provided in references (*1, 2*).

2) Since the second real data application involves many and mostly binary traits while the given simulation set-ups did not seem to cover such a case, having some simulation results under a set-up mimicking such a case would help.

Response:

Thank you for your comment. We have now included such a simulation and are including the results below. For the simulation, we followed closely to the real data application #2 and generated data which was primarily binary outcomes with some count outcomes. We generated the data following the pattern described in simulation Scenario 1, using three underlying latent factors and setting phenotype heritability to be 5%.

Table R1. Simulation results based on scenario 1 (three true latent factors, heritability=5%, mixed) with different proportions of binary traits

| **%Binary** | **AUC** | **FDR (Nominal = 0.1)** | **Power** |
| --- | --- | --- | --- |
| **90%** | 0.98 (0.01) | 0.02 (0.01) | 0.92 (0.02) |
| **40%** | 0.99 (0.00) | 0.03 (0.01) | 0.94 (0.02) |
| **0%** | 1.00 (0.00) | 0.01 (0.01) | 0.98 (0.01) |

As shown in Table R1, FM-GPT generally maintains high AUC and power across different proportions of binary traits, with FDR remaining well controlled. The results have also been included in the Supplement.

3) Given that Eq (3) is the main technical innovation, its discussion is too sketchy. For example, it is not clear what the purpose of the proposed transformation is and how it is done for a discrete RV. It did not help much even after I studied Section 3 and the cited reference [57].

Response:

Thank you for your comment. We have now updated the text (Method Section 1 and 3) to include a more explanatory derivation to show why we introduce the transformation, and how doing so allows us to jointly model the continuous and discrete random variables. Briefly, equation (4) defines the augmented response used in the data augmentation scheme (see Section 3), whereas equation (3) links this augmented response to the continuous latent factors through the factor model. Note that equation (4) should not be interpreted as a deterministic transformation of an observed discrete random variable into a Gaussian random variable. Instead, for discrete outcomes, we introduce a Polya-Gamma auxiliary variable ($\psi$). Conditional on $\psi$, the likelihood contribution for the natural parameter $\theta$, in this case $\Lambda F$ in equation (3), is proportional to a Gaussian kernel. This conditional Gaussian representation allows continuous and discrete outcomes to be handled within a unified latent-factor framework while preserving the original discrete likelihood after integrating over the auxiliary variable. The key idea of the Polya-Gamma data augmentation strategy (*1, 2*) is to introduce an auxiliary variable $\psi_{ik}$~$PG\left( \xi_{ik},0 \right)$, which augments the likelihood for discrete outcomes so that conditional on $\psi_{ik}$, the likelihood contribution for the natural parameter becomes proportional to a Gaussian kernel. $\xi_{ik}$varies depending on the data type of $y_{ik}$. For example, for a binary outcome, $\xi_{ik}=1$; for a multinomial outcome, $\xi_{ik}=M_{k}$, where $M_{k}$ is the total number of outcomes; for count outcome using negative binomial distribution, $\xi_{ik}=y_{ik}+r_{k}$, where $r_{k}$ is the target number of successes in negative binomial model. For discrete outcomes including binary, multinomial and negative binomial based outcomes, the likelihood can be written in the logistic form: $p_{k}\left( y_{ik} | \theta_{ik} \right)=C\frac{\left\{ \exp\left( \theta_{ik} \right) \right\}^{y_{ik}}}{\left\{ 1+\exp\left( \theta_{ik} \right) \right\}^{\xi_{ik}}}$, where $C$ is a normalizing constant and $\theta_{ik}=\Lambda_{k}'F_{i}$ is the natural parameter in our model. It can be shown that for continuous outcomes: $p\left( \theta_{ik} | y_{ik} \right)\propto p\left( \theta_{ik} \right)\exp\left\{ -\frac{1}{2}\left( \theta_{ik}-y_{ik} \right)^{2} \right\}$, and for discrete outcomes: $p\left( \theta_{ik} | {y_{ik}, \psi}_{ik},\xi_{ik} \right)\propto p\left( \theta_{ik} \right)\exp\left\{ -\frac{\psi_{ik}}{2}\left( \theta_{ik}-\frac{y_{ik}-0.5\xi_{ik}}{\psi_{ik}} \right)^{2} \right\}$. We can unify the continuous and discrete outcomes with the following conditional density of $\theta_{ik}:p\left( \theta_{ik} | {y_{ik}, \psi}_{ik},\xi_{ik} \right)\propto p\left( \theta_{ik} \right)\exp\left\{ -\frac{\psi_{ik}}{2}\left( {\theta_{ik}-\tilde{y}}_{ik} \right)^{2} \right\}$, which implies the likelihood is the kernel of the Gaussian density. This data augmentation provides a unified likelihood representation for continuous and discrete phenotypes, enabling the use of conjugate conditional posteriors within a Gibbs sampling framework.

4) Based on the discussion of Eq (3), it seems to me that the proposed method can only handle two types of non-normal traits: binary (Bernoulli) and negative binomial-based count data. It would be better to state it out clearly what types of continuous and discrete traits are covered because “mixed types of traits” can be more general.

Response:

Thank you for your comment. The proposed FM-GPT method can handle continuous Gaussian traits, as well as discrete traits whose likelihood can be written in the Polya-Gamma compatible logistic form: $p_{k}\left( y_{ik} | \theta_{ik} \right)=C\frac{\left\{ \exp\left( \theta_{ik} \right) \right\}^{y_{ik}}}{\left\{ 1+\exp\left( \theta_{ik} \right) \right\}^{\xi_{ik}}}$, i.e. conditional on $\psi$, the likelihood contribution of these discrete traits for the natural parameter $\theta$, is proportional to a Gaussian kernel. Such discrete outcomes could include binary, multinomial and negative binomial based outcomes. For example, for a binary outcome, $\xi_{ik}=1$; for a multinomial outcome, $\xi_{ik}=M_{k}$, where $M_{k}$ is the total number of outcomes; for count outcome using negative binomial distribution, $\xi_{ik}=y_{ik}+r_{k}$, where $r_{k}$ is the target number of successes in negative binomial model. However, the FM-GPT is not restricted to only these. We have updated the text in Methods, Section 3 to more specifically state which types of discrete outcomes may be modeled by FM-GPT in the Polya-Gamma framework. In some cases, simple dummy variable coding can also be used for multinomial outcomes.

5) It is stated: “Unlike standard unsupervised factor analysis which assumes a single global set of latent factors, our model allows the latent factors to be specific to each genomic region under study.” Where and how?

Response:

Thank you for your comment. In standard unsupervised factor analysis, latent factors are derived solely from the outcome matrix and assumes a single global factor structure shared across all genomic regions. In contrast, FM-GPT analyzes each genomic region separately (in Method section 1, equations 1-3 is applied to each t-th (1≤t≤T) genomic region of interest we wish to fine map; also see updated Fig 1) and jointly models the outcome Y with the predicted expression of genes X within that region. Consequently, the latent factors are estimated conditional on the region-specific gene expression signals rather than being learned solely from the outcomes. Because different genomic regions contain different sets of genes and exhibit distinct patterns of association with the outcomes, the resulting latent factors and factor loadings are allowed to vary across regions. This region-specific factorization is biologically appropriate for fine mapping because regulatory architecture is often local and context dependent: genes within the same locus may influence a subset of related traits through shared cis-regulatory mechanisms, whereas genes in different loci may act through distinct biological pathways thus influence a different subset of traits. Allowing the latent structure to vary by region therefore avoids imposing common alignment of factors across loci and better reflects the locus-specific mechanisms that fine mapping aims to distinguish. We have now edited the Method section 1 to make this more clear.

6) Since applying (sparse) PCA seems to be straightforward for GWAS/TWAS of multiple traits, how would the proposed method differ from PCA/sPCA?

I think that one downside of the proposed method, as one based on (s)PCA, is the loss of specificity: even with sparse leadings, one may not know which associations are for one specific trait. Some discussion may be needed.

Response:

Thank you for your comment. We have now included simulations (Table S1a) comparing the performance of sPCA to the performance of FM-GPT and regular exploratory factor analysis. Notably, we compare the Sensitivity, Specificity and Youden Index of all three methods. When implementing sPCA, we use five-fold cross-validation to determine the value of the sparsity tuning parameter. EFA does not have exact zero loadings, so negligible loadings, e.g. <0.1 by convention, are suppressed to zeros for fair comparison. FM-GPT uses an infinite factor model with a gamma process shrinkage prior, which induces continuous shrinkage rather than exact sparsity (we have now corrected the wording to make this more clear). Thus, its loadings are shrunk toward zero but are not generally estimated as exact zeros. For a clearer visualization and for fair comparison, we first applied a post-hoc unit-scaling procedure to normalize the loading vector for each factor and then suppressed negligible loadings, e.g.<0.1 to zeros as in EFA (see Supplement Methods S4: Post-hoc unit-scaling and thresholding for factor loadings). We have updated the manuscript Methods section 2 and Supplementary Methods S4 to reflect these changes.

Table R2. Factor loading structure recovery comparison of FM-GPT with EFA and sPCA based on simulation scenario 1-2 (one true latent factor) with different outcome types and heritability levels

|  |  |  | Youden Index | | | Sensitivity | | | Specificity | | |
| --- | --- | --- | --- | --- | --- | --- | --- | --- | --- | --- | --- |
| Scenario | Outcome data type | Heritability % | FM-GPT | EFA | sPCA | FM-GPT | EFA | sPCA | FM-GPT | EFA | sPCA |
| 1. Homogeneous | Continuous | 1 | 0.73 (0.01) | 0.66 (0.02) | 0.59 (0.02) | 0.78 (0.01) | 0.70 (0.01) | 0.61 (0.01) | 0.96 (0.00) | 0.96 (0.00) | 0.99 (0.00) |
|  |  | 3 | 0.76 (0.01) | 0.67 (0.02) | 0.60 (0.01) | 0.79 (0.01) | 0.72 (0.01) | 0.62 (0.01) | 0.97 (0.00) | 0.96 (0.00) | 0.99 (0.00) |
|  |  | 5 | 0.79 (0.01) | 0.70 (0.01) | 0.63 (0.01) | 0.82 (0.01) | 0.74 (0.01) | 0.64 (0.01) | 0.97 (0.00) | 0.96 (0.00) | 0.99 (0.00) |
|  | Mixed | 1 | 0.47 (0.02) | 0.37 (0.02) | 0.31 (0.01) | 0.54 (0.01) | 0.44 (0.01) | 0.32 (0.01) | 0.94 (0.00) | 0.93 (0.00) | 0.99 (0.00) |
|  |  | 3 | 0.57 (0.02) | 0.39 (0.02) | 0.31 (0.01) | 0.62 (0.01) | 0.46 (0.01) | 0.32 (0.01) | 0.95 (0.00) | 0.93 (0.00) | 0.99 (0.00) |
|  |  | 5 | 0.60 (0.02) | 0.40 (0.02) | 0.32 (0.01) | 0.65 (0.01) | 0.47 (0.01) | 0.34 (0.01) | 0.95 (0.00) | 0.93 (0.00) | 0.98 (0.00) |
| 2. Heterogeneous | Continuous | 1 | 0.79 (0.01) | 0.71 (0.02) | 0.66 (0.01) | 0.83 (0.01) | 0.75 (0.01) | 0.66 (0.01) | 0.96 (0.00) | 0.96 (0.00) | 0.99 (0.00) |
|  |  | 3 | 0.79 (0.01) | 0.71 (0.01) | 0.67 (0.01) | 0.82 (0.01) | 0.74 (0.01) | 0.68 (0.01) | 0.97 (0.00) | 0.97 (0.00) | 0.99 (0.00) |
|  |  | 5 | 0.78 (0.01) | 0.75 (0.01) | 0.67 (0.01) | 0.82 (0.01) | 0.79 (0.01) | 0.68 (0.01) | 0.96 (0.00) | 0.97 (0.00) | 0.99 (0.00) |
|  | Mixed | 1 | 0.51 (0.01) | 0.33 (0.02) | 0.25 (0.01) | 0.57 (0.01) | 0.41 (0.01) | 0.27 (0.01) | 0.94 (0.00) | 0.92 (0.00) | 0.98 (0.00) |
|  |  | 3 | 0.54 (0.02) | 0.37 (0.02) | 0.34 (0.01) | 0.60 (0.01) | 0.44 (0.01) | 0.35 (0.01) | 0.94 (0.00) | 0.92 (0.00) | 0.99 (0.00) |
|  |  | 5 | 0.58 (0.02) | 0.43 (0.02) | 0.34 (0.01) | 0.63 (0.01) | 0.50 (0.01) | 0.36 (0.01) | 0.95 (0.00) | 0.93 (0.00) | 0.99 (0.00) |

In evaluating the recovery of factor loading structures, sPCA has the benefit of enforcing sparsity in the loading matrix, leading to overall highest specificity (Table R2). However, because of its high specificity and sparse structure, sPCA tends to be less sensitive and have too many false negatives, i.e. missing the true nonzero loadings. EFA on the other hand, has better sensitivity but lower specificity. Finally, FM-GPT has an improved specificity over EFA while in the same time maintaining the highest sensitivity among three methods. From the Youden Index (Sensitivity + Specificity – 1), we can see that in each case, FM-GPT outperforms both EFA and sPCA in discovering the latent loading structure.

In addition to the current version with GIFT and mvIWAS factor extracted from EFA, we also compared FM-GPT to GIFT and mvIWAS when using principal components extracted from sPCA (see Table R3).

Table R3. Comparison of FM-GPT with GIFT and mvIWAS + factor extracted by EFA or sPCA based on simulation scenario 1 with three true latent factors and heritability=5%, continuous only

| **Method** | **Factor Extraction** | **AUC** | **FDR** | **Power** |
| --- | --- | --- | --- | --- |
| **GIFT Factor** | EFA | 0.94 (0.02) | 0.24 (0.03) | 0.88 (0.03) |
|  | sPCA | 0.93 (0.02) | 0.23 (0.03) | 0.89 (0.03) |
| **mvIWAS Factor** | EFA | 0.98 (0.01) | 0.15 (0.03) | 0.92 (0.03) |
|  | sPCA | 0.98 (0.01) | 0.15 (0.03) | 0.92  (0.03) |
| **FM-GPT** | Bayesian Factor | 1.00 (0.00) | 0.01 (0.01) | 1.00 (0.01) |

Overall, FM-GPT outperformed GIFT or mvIWAS with factors extracted by EFA or sPCA in variable selection, statistically more powered without inflating the FDR.

2. Some proposed methods output p-values (while the proposed is based on FDR); for comparison, the former were transformed to FDR, which is not easy (and is approximate with errors) with correlated p-values. Thus, it may not be fair to compare the methods in that way; one may ask why not compare the methods based on p-values or family-wise type I error control? While I feel that the authors’ comparison might be useful, such limitation has to be acknowledged.

BTW, the authors proposed combining the results of single-trait analyses using Fisher’s method, which, strictly speaking, may not be valid because those traits/p-values are not independent.

Response:

We thank the reviewer for the comment. We agree that direct comparison between Bayesian and frequentist methods is inherently challenging because they quantify uncertainty using different inferential frameworks. FM-GPT is a Bayesian method that outputs posterior inclusion probabilities (PIP) and Bayesian false discovery rates (BFDR), whereas several competing methods produce p-values. Consequently, FM-GPT does not naturally provide p-values, and converting posterior probabilities into p-values would not be statistically meaningful. For the frequentist methods, we used BH-adjusted p-values to control the false discovery rate because FDR control is the most widely adopted criterion in high-dimensional genomic studies and is conceptually closest to the BFDR criterion used by FM-GPT [concept of BFDR first proposed by Newton 2004(*3*) in genomic context]. We acknowledge that BH adjustment is approximate under correlated tests and that Bayesian and frequentist FDR measures are not identical. Our primary evaluation in simulations was based on AUC, which is rank-based and do not depend on the specific significance measure or cutoff used by each method (Fig 2a-b, 3a-b). For a more comprehensive comparison, we additionally applied the Benjamini–Yekutieli (BY) procedure(*4*) which provides FDR control under arbitrary dependence structures and is generally more robust to correlated p-values than the BH procedure. Furthermore, to avoid potential biases arising from the conversion of p-values to FDR-adjusted significance measures, we performed supplementary comparisons based on raw p-values and family-wise error rate (FWER) control. Table R4 show the comparison of power and true FDR control between our method vs. other frequentist methods in simulation scenario 1 (heritability of 3% and three true underlying factors, continuous only). Across all evaluation criteria, FM-GPT consistently identified a greater number of true causal genes while maintaining appropriate error control regardless than all the frequentist methods regardless of the different measures/cutoffs used. Among the frequentist approaches, BH- and BY-adjusted FDR cutoffs tended to perform best and most closely matched the BFDR-based results. These findings demonstrate that the superior performance of FM-GPT is not driven by the particular significance measure or thresholding strategy used, but rather reflects its improved ability to prioritize truly causal genes.

We acknowledge that fisher’s method requires independent p-values, however, Fisher’s method is used in our framework solely as an initial screening procedure to prioritize genomic regions for subsequent fine-mapping analysis, rather than as the final inferential step for gene discovery. Consequently, the purpose of this stage is to rank and identify potentially relevant regions rather than to provide formal significance testing or control false positive rates. The downstream Bayesian fine-mapping procedure is then applied to the selected regions and serves as the primary mechanism for identifying causal genes and controlling false discoveries. We chose Fisher’s method because of its simplicity, computational efficiency, and strong power for aggregating moderate signals across multiple traits. Nevertheless, alternative p-value combination methods that are robust to correlated tests, such as the Cauchy combination test or the aggregated Cauchy association test (ACAT)(*5*), could also be employed in this screening stage. We have added a discussion of this limitation and these potential alternatives in the revised manuscript (Results: overview of FM-GPT method, Methods section 4).

Table R4. Comparison of different p-value adjustment methods for frequentist methods with FM-GPT using BFDR on true FDR control and power in the simulation scenario 1 (heritability of 3% and three true underlying factors, continuous only; nominal FDR=0.1).

True FDR:

| **Adjustment Type** | **GIFT Factor** | **GIFT Meta** | **mvIWAS Factor** | **mvIWAS Meta** | **FM-GPT** |
| --- | --- | --- | --- | --- | --- |
| **Raw P-Values** | 0.32 (0.01) | 0.26 (0.01) | 0.41 (0.01) | 0.37 (0.01) | X |
| **Bonferroni FWER Adjustment** | 0.07 (0.01) | 0.05 (0.01) | 0.09 (0.01) | 0.11 (0.01) | X |
| **BH FDR Adjustment** | 0.11 (0.01) | 0.08 (0.01) | 0.16 (0.01) | 0.16 (0.01) | X |
| **BY FDR Adjustment** | 0.06 (0.01) | 0.04 (0.00) | 0.07 (0.01) | 0.09 (0.01) | X |
| **Bayesian PIP** | X | X | X | X | 0.02 (0.01) |
| **Bayesian FDR** | X | X | X | X | 0.01 (0.01) |

Power:

| **Adjustment Type** | **GIFT Factor** | **GIFT Meta** | **mvIWAS Factor** | **mvIWAS Meta** | **FM-GPT** |
| --- | --- | --- | --- | --- | --- |
| **Raw P-Values** | 0.83 (0.01) | 0.88 (0.01) | 0.86 (0.01) | 0.90 (0.01) | X |
| **Bonferroni FWER Adjustment** | 0.74 (0.01) | 0.83 (0.01) | 0.79 (0.01) | 0.84 (0.01) | X |
| **BH FDR Adjustment** | 0.76 (0.01) | 0.84 (0.01) | 0.81 (0.01) | 0.85 (0.01) | X |
| **BY FDR Adjustment** | 0.71 (0.01) | 0.81 (0.01) | 0.77 (0.01) | 0.83 (0.01) | X |
| **Bayesian PIP** | X | X | X | X | 0.89 (0.01) |
| **Bayesian FDR** | X | X | X | X | 0.89 (0.01) |

3. The quality of some figures is not high: for example, I cannot read the numbers in Figs 2&3, especially panels (C) and (D). Please give concrete results that the proposed method can control FDR effectively.

Response:

Thank you for your comment. We have revised panels (C) and (D) in Figures 2 and 3 to improve their resolution and readability. In addition, following the reviewers’ suggestion, we now report true FDR at the nominal level of 0.1 rather than raw counts of true and false discoveries, providing a more direct assessment of FDR control (see updated Figure (E)-(F) below and complete updated figure2-3 in the revised manuscript). We also added two showing the power to detect causal genes across varying numbers of underlying factors and phenotype heritability levels (see updated Figure (C)-(D) below and complete updated Figure 2, 3 in the revised manuscript). As shown in these updated results, FM-GPT maintains well-controlled FDR while achieving high power across both homogeneous and heterogeneous scenarios, under varying heritability levels, numbers of latent factors, and both continuous and mixed outcome types. Following the reviewers’ comments, we additionally included GIFT-Meta and mvIWAS-Meta in the comparison. Although these methods achieved higher power in some scenarios, this gain was accompanied by a substantially increased number of false positives thus loss of FDR control. The updated Figures 2 and 3 have been included in the revised manuscript.

Revised Fig 2C-D

Revised Fig 2E-F

 Revised Fig 3C-D

Revised Fig 3E-F

**Reviewer #2**: In this manuscript, the authors introduced a Bayesian fine-mapping framework that jointly models multiple correlated phenotypes and prioritizes genes through a GreX-informed factor analysis. The framework is flexible and addresses an important problem in large-scale phenome-wide TWAS analysis. However, several aspects of the methodology, interpretation, and evaluation require further clarification. I recommend major revision. Detailed comments are provided below.

1. The manuscript frequently refers to genes identified by fine-mapping methods as "causal". TWAS-based approaches detect associations between genetically predicted expression and traits, which may not be causal relationships. I recommend consistently using more accurate language, such as "putatively causal" or "prioritized genes".

Response:

Thank you for your comment. We have paid careful attention to this important distinction and adjusted the text throughout the entire manuscript to “putatively causal” or “prioritized genes” where appropriate.

2. The manuscript highlights "gene-guided dimension reduction" as a key methodological innovation. This is mainly described at a conceptual level but is not sufficiently clarified in terms of the model formulation. From Equation (2), it appears that latent factors are modeled as linear combinations of GReX, which constrains the factor space to lie within the span of predicted gene expression. This is a strong modeling assumption with important implications. The authors should clarify how gene expression "guides" the factor analysis, and how this approach relates to existing frameworks such as supervised factor models.

Response:

We thank the reviewer for this insightful comment. We agree that “gene-guided dimension reduction” was previously described mainly at a conceptual level, and we have revised the manuscript Methods section 1 to clarify the model formulation, underlying assumptions, and relationship to supervised factor models.

In FM-GPT, the latent factors are modeled as linear combinations of genetically regulated expression (GReX) within a genomic region, as shown in Equation (2). Thus, the factor space is constrained to lie within the span of the region-specific GReX variables. This is a strong assumption but also an intentional modeling choice. The goal of FM-GPT is not to identify latent phenotypic structure in an unsupervised manner, as in conventional factor analysis, but rather to capture latent axes of phenotypic variation that are driven by genetically regulated transcriptional mechanisms. By constructing factors from GReX, FM-GPT focuses dimension reduction on variation attributable to candidate genes in the locus, thereby improving biological interpretability and facilitating causal gene prioritization.

We agree that this formulation is closely related to supervised or predictor-guided factor models (e.g. Bernardo et al. 2003(*6*); Carvalho et al., 2008(*7*); Bhattacharya and Dunson, 2011(*8*)), in which external covariates are used to inform latent factor construction. However, FM-GPT differs from traditional supervised factor models in that it uses region-specific GReX variables and embeds the resulting latent representation within a Bayesian fine-mapping framework. The latent factors therefore serve a dual purpose: they capture shared genetically regulated effects across phenotypes and provide a lower-dimensional representation through which causal genes can be probabilistically prioritized within each genomic region.

From a biological perspective, complex traits are rarely driven by single genes acting in isolation. Rather, genetic effects often propagate through regulatory networks, molecular pathways, and tissue-specific transcriptional programs that jointly influence downstream phenotypes(*9, 10*). Extensive pleiotropy across human traits further suggests that correlated phenotypes may reflect perturbations of shared biological mechanisms (*11*). FM-GPT operationalizes this idea by summarizing region-specific GReX signals into latent transcriptionally informed factors, thereby enabling gene-guided dimension reduction focused on biologically meaningful variation relevant to fine mapping.

We have revised the manuscript Results and Methods sections to clarify these points and discuss the relationship between FM-GPT and supervised factor modeling approaches in the revised manuscript.

3. The model assumes region-specific latent factors, such that each genomic region has its own latent factor structure. This provides flexibility in modeling heterogeneity, but it makes the factors identified in different regions not directly comparable. It is not clear how the inferred factors are interpreted biologically and how "shared mechanisms" across regions are defined. Please clarify.

Response:

We thank the reviewer for this insightful comment. We agree that the region-specific latent factors estimated by FM-GPT are not directly comparable across genomic regions in the same way that factors from a global factor model would be.

Our framework intentionally estimates latent factors separately within each genomic region because the primary goal is to characterize the local genetic architecture of that region and identify genes whose genetically regulated expression contributes to shared variation across multiple phenotypes. Consequently, the latent factors are region-specific constructs whose interpretation depends on the genes and phenotypes associated with that particular region. We do not assume that Factor 1 from one region corresponds to Factor 1 from another region, nor do we attempt to align latent factors across regions.

The biological interpretation of a latent factor is therefore based on the pattern of phenotype loadings and the genes contributing to that factor within a region. A factor can be viewed as representing a latent transcriptional mechanism through which one or more genes influence a subset of correlated phenotypes. Different genomic regions may yield distinct latent factors that nevertheless affect similar groups of phenotypes or implicate related biological pathways.

Although the models were fit independently in different genomic regions, in our real data example 2, we observed that several dominant genes from distinct regions (e.g. *DDB1, BLM, HECTD1*) generated highly similar latent factor loading patterns, corresponding to largely overlapping subsets of phenotypes. While this does not imply that the latent factors themselves are identical or directly comparable across regions, it suggests that genes from different genomic loci may influence similar phenotype clusters and potentially converge on related biological processes. This is the sense in which FM-GPT can reveal evidence of shared mechanisms across regions despite estimating region-specific latent factors. To avoid confusion, we have removed the word “shared biological mechanism” in the revised manuscript as the method was not designed for this purpose.

We have revised the manuscript (Results and Methods section 1) to clarify this more clearly.

4. In Methods subsection 4 (page 12-13), the factor-specific BFDR is defined by aggregating PIPs across all genes from all genomic regions for a given factor index l. However, factor l in different regions may correspond to different latent structures. Please clarify how factor indices are aligned across regions, or alternatively restrict BFDR calculations to be region-specific, that is, indexed by both l and t. If only the omnibus gene-level BFDR is used in practice, this should be explicitly stated.

Response:

We thank the reviewer for this important observation. We agree that because latent factors are estimated separately within each genomic region, factor indices are not guaranteed to correspond to the same underlying latent structure across regions. Consequently, aggregating PIPs across regions based solely on a common factor index may not be well justified and could lead to ambiguous interpretation.

To address this concern, we have now removed the factor-specific BFDR from the manuscript. In the revised version, we report only the omnibus gene-level BFDR, which aggregates evidence across all latent factors within a region and serves as the primary criterion for gene prioritization and statistical inference. Since the omnibus BFDR does not rely on alignment or comparability of latent factor indices across regions, it avoids the issue raised by the reviewer and is more consistent with the intended interpretation of our region-specific latent factor framework. We have revised the Methods Section 4 accordingly and clarified that all reported discoveries are based on the omnibus gene-level BFDR.

5. Latent factor models are inherently non-identifiable due to rotation and sign invariance. While sparsity constraints in priors partially mitigate this issue, they do not fully resolve it. The orientation and sign of inferred factors are not uniquely defined, and the biological interpretation may depend on arbitrary choices. I suggest the authors to assess the stability of inferred factors. In particular, they can repeat model fitting with different random seeds and initializations, examine whether selected genes and factor loading patterns are stable, and report concordance of inferred factors across runs. For real data applications, they can also perform bootstrap to assess whether the reported phenotype axes, such as the immune-metabolic axis, are reproducible.

Response:

Thank you for your comment. We have now performed additional sensitivity analysis to evaluate the stability of the inferred factors and gene ranking. In particular, we have now re-run both the first and second real data examples under different circumstances:

**Real data example 1**: we reran FM-GPT on the genomic regions containing the 18 genes originally selected in the manuscript under two sensitivity settings: (i) five runs with different initialization points using the full dataset, and (ii) five runs using random 90% subsamples of the original dataset, each with different initialization points. We evaluated the robustness of both gene ranking and factor loading patterns.

We summarized the 18 originally selected genes by comparing their original rankings reported in the manuscript with their rankings based on the average PIP across the five random initialization runs and the average PIP across the five random 90% subsampling runs. We also compared the corresponding factor loading structures across these analyses. These results are presented in Table R5 and Figure R1.

Table R5. Comparison of ranking of originally selected 18 genes from five random initialization runs or five random 90% subsampling runs with original ranking in real data example 1

| **Gene** | **Original PIP** | **Original Ranking** | **Average ranking of five random initializations** | **Average ranking of five subsamples** |
| --- | --- | --- | --- | --- |
| **PRDM5** | 1 | 1 | 3 | 3 |
| **WNT7A** | 1 | 2 | 1 | 1 |
| **BCAS3** | 1 | 3 | 2 | 2 |
| **LRRC37A** | 0.996 | 4 | 4 | 5 |
| **ARHGEF10L** | 0.9765 | 5 | 5 | 9 |
| **NOS2P3** | 0.916 | 6 | 6 | 4 |
| **TNFAIP2** | 0.9065 | 7 | 7 | 7 |
| **SYPL2** | 0.8735 | 8 | 12 | 8 |
| **VASH1** | 0.8585 | 9 | 11 | 17 |
| **ARL17B** | 0.8285 | 10 | 10 | 11 |
| **MSH3** | 0.8135 | 11 | 16 | 14 |
| **UBB** | 0.763 | 12 | 8 | 13 |
| **MDM4** | 0.7625 | 13 | 14 | 12 |
| **ZNF365** | 0.757 | 14 | 13 | 16 |
| **JCAD** | 0.7415 | 15 | 18 | 18 |
| **FBLN2** | 0.7195 | 16 | 9 | 10 |
| **CDNF** | 0.716 | 17 | 15 | 6 |
| **ADGRV1** | 0.714 | 18 | 17 | 15 |

The PIP-based gene rankings and factor loading patterns were broadly consistent between the original analysis and the sensitivity analyses based on random initializations and 90% subsampling (Table R5, Fig R1). For the 18 originally selected genes, the average Kendall’s (W) measure of rank agreement across the original ranking and the five rankings obtained from random-initialization reruns was **0.88 (95% CI: 0.79–0.94)**. This was calculated by first computing Kendall’s (W) for each of the $\binom{6}{2}$=15 pairwise comparisons among the original ranking and the five rerun rankings, and then averaging these values. Confidence intervals were obtained using bootstrap resampling. Applying the same procedure to the 90% subsampling analyses yielded an average Kendall’s (W) of **0.76 (95% CI: 0.62–0.88)**, indicating substantial agreement between the original gene rankings and those obtained under random subsampling.

We further evaluated the reproducibility of the factor loading structures and observed a high degree of agreement between the original loading matrix and the loading matrices obtained from both random-initialization and 90% subsampling reruns. For the random-initialization analyses, the **average Jaccard index** across the $\binom{6}{2}$=15 pairwise comparisons was **0.81 (95% CI: 0.79–0.82)**, with an average sensitivity of **0.88 (95% CI: 0.87–0.89)** and an average specificity of **0.84 (95% CI: 0.82–0.86)**. For the 90% subsampling analyses, the average Jaccard index was **0.86 (95% CI: 0.85–0.88)**, with an average sensitivity of **0.92 (95% CI: 0.91–0.93)** and an average specificity of **0.88 (95% CI: 0.86–0.89)**. Together, these results suggest that both gene prioritization and factor loading detection are robust to initialization choices and moderate perturbations of the sample.

Fig R1. Comparison of factor loadings from five random initialization runs or five random 90% subsampling runs with original results in real data example 1

**Real data example 2**: we reran FM-GPT on the genomic regions containing the 60 genes originally selected in the manuscript under two sensitivity settings: (i) five runs with different initialization points using the full dataset, and (ii) five runs using random 90% subsamples of the original dataset, each with different initialization points. We evaluated the robustness of both gene prioritization and factor loading patterns.

Table R6. Comparison of ranking of originally selected 60 genes from five random initialization runs or five random 90% subsampling runs with original ranking in real data example 2

| **Gene** | **Original PIP** | **Original Ranking** | **Average ranking of five random initializations** | **Average ranking of five subsamples** |
| --- | --- | --- | --- | --- |
| **SKI** | 1 | 1 | 2 | 22 |
| **DMAP1** | 1 | 2 | 37 | 30 |
| **EIF2B3** | 1 | 3 | 5 | 2 |
| **PRDX1** | 1 | 4 | 13 | 35 |
| **NSUN4** | 1 | 5 | 33 | 37 |
| **HOOK1** | 1 | 6 | 11 | 3 |
| **PSMD4** | 1 | 7 | 34 | 44 |
| **B4GALT3** | 1 | 8 | 16 | 12 |
| **SDHC** | 1 | 9 | 40 | 5 |
| **FCGR3A** | 1 | 10 | 17 | 14 |
| **RGS5** | 1 | 11 | 4 | 31 |
| **UXS1** | 1 | 12 | 6 | 45 |
| **NUDT16** | 1 | 13 | 28 | 25 |
| **BAG6** | 1 | 14 | 19 | 34 |
| **SRSF3** | 1 | 15 | 54 | 11 |
| **CDC14B** | 1 | 16 | 43 | 6 |
| **PCGF6** | 1 | 17 | 46 | 32 |
| **SFXN4** | 1 | 18 | 30 | 33 |
| **ABCC8** | 1 | 19 | 25 | 19 |
| **GTF2H1** | 1 | 20 | 9 | 24 |
| **TSG101** | 1 | 21 | 21 | 27 |
| **MS4A7** | 1 | 22 | 20 | 7 |
| **MS4A14** | 1 | 23 | 44 | 10 |
| **DDB1** | 1 | 24 | 36 | 28 |
| **EMC9** | 1 | 25 | 35 | 36 |
| **AP4S1** | 1 | 26 | 22 | 39 |
| **HECTD1** | 1 | 27 | 15 | 21 |
|  | 1 | 28 | 7 | 46 |
| **DTD2** | 1 | 29 | 10 | 47 |
| **ARID4A** | 1 | 30 | 31 | 48 |
| **RFX7** | 1 | 31 | 27 | 49 |
| **BLM** | 1 | 32 | 32 | 15 |
| **RAB40B** | 1 | 33 | 41 | 8 |
| **SLC44A2** | 1 | 34 | 18 | 4 |
| **ZNF576** | 1 | 35 | 12 | 16 |
| **SMG9** | 1 | 36 | 3 | 17 |
| **ZNF404** | 1 | 37 | 42 | 18 |
| **ZNF45** | 1 | 38 | 38 | 23 |
| **ZNF234** | 1 | 39 | 8 | 29 |
| **BCL3** | 1 | 40 | 1 | 26 |
| **KCNJ14** | 1 | 41 | 45 | 50 |
| **MAMSTR** | 1 | 42 | 23 | 9 |
| **LIN7B** | 1 | 43 | 39 | 13 |
| **NFATC2** | 1 | 44 | 14 | 1 |
| **SERHL2** | 1 | 45 | 29 | 38 |
| **TTLL12** | 1 | 46 | 24 | 40 |
| **PIM3** | 1 | 47 | 26 | 20 |
| **TMEM218** | 0.7235 | 48 | 48 | 51 |
| **CCN1** | 0.576 | 49 | 55 | 42 |
| **PACRGL** | 0.51 | 50 | 49 | 52 |
| **KCNIP4** | 0.49 | 51 | 56 | 53 |
| **ANKRD6** | 0.4035 | 52 | 47 | 54 |
| **CASP8AP2** | 0.4025 | 53 | 57 | 55 |
| **SEPTIN3** | 0.3595 | 54 | 58 | 56 |
| **IL33** | 0.326 | 55 | 59 | 41 |
| **COPA** | 0.322 | 56 | 50 | 57 |
| **CCDC134** | 0.264 | 57 | 60 | 58 |
| **DAG1** | 0.254 | 58 | 51 | 59 |
| **DHX30** | 0.2465 | 59 | 52 | 43 |
| **DALRD3** | 0.2405 | 60 | 53 | 60 |

For real data example 2, we observed good agreement in PIP-based gene rankings across sensitivity analyses (Table R6). Among the 60 genes originally selected in the manuscript, the average pairwise rank agreement across the $\binom{6}{2}$=15 comparisons among the original ranking and the five random-initialization reruns was **0.74 (95% CI: 0.67–0.81)**. For the 90% subsampling analyses, the corresponding average pairwise rank agreement was **0.81 (95% CI: 0.73–0.88)**.

We further compared the factor loading matrices by examining the heatmap originally included in the manuscript alongside heatmaps averaged across the random-initialization reruns and the random 90% subsampling reruns (Fig R2). The immune–metabolic axis described in the manuscript remained clearly visible across these sensitivity analyses, and the overall loading patterns were largely preserved.

For the random-initialization analyses, the **average Jaccard index** comparing detected nonzero loading patterns with the original manuscript results was **0.65 (95% CI: 0.63–0.67)**, with average sensitivity of **0.73 (95% CI: 0.71–0.75)** and average specificity of **0.88 (95% CI: 0.87–0.89)**. For the 90% subsampling analyses, the average Jaccard index was **0.68 (95% CI: 0.66–0.70)**, with average sensitivity of **0.78 (95% CI: 0.76–0.80)** and average specificity of **0.85 (95% CI: 0.84–0.87)**. Together, these results indicate that the major gene-prioritization and factor-loading structures in real data example 2 are reasonably robust to both initialization choices and moderate sample perturbation.

Fig R2. Comparison of factor loadings from five random initialization runs or five random 90% subsampling runs with original results in real data example 2

6. Figure 1A can be improved by clarifying key model structures. In particular, the relationship between GReX, latent factors F, and phenotypes Y is ambiguous, and may be misinterpreted as standard unsupervised factor analysis. The figure presents the method as a sequential pipeline, whereas the model is actually jointly estimated. Also, key aspects such as region-specific modeling and sparsity-inducing priors are not illustrated.

Response:

Thank you for your comment. We have now modified Figure 1 by removing the sequential pipeline, and including the main characteristics of the model (e.g. region-specific modeling, different types of sparsity inducing/shrinkage priors, as well as the data augmentation part that handles mixed types of outcomes) and reorganizing the panels. See the updated Fig 1 below.

## Figure 1. A schematic overview of the FM-GPT method. (A) Demonstration of phenome-wide TWAS results, we will apply fine mapping to each of those highlighted genomic regions. (B) A directed acyclic graph (DAG) showing the main prior settings that characterize the FM-GPT method. $Z^{R}$ and $X^{R}$ correspond to the genotype and gene expression data from reference panel; $Z^{R}$and $\hat{X}^{G}$ are the genotype estimated GReX data from GWAS; $F$ is the latent phenotypic factors and $Y$ is the multiple phenotypes potentially of mixed data types; (1)-(4) correspond to Equation (1)-(4) in the Methods section. (C-D) Main outputs from the FM-GPT method: the putatively causal genes for the phenotype factors (C); the loadings of phenotypes on each phenotype factor (D).

7. In Figures 2C–D and 3C–D, the reported numbers of TP and FP appear to be aggregated over many simulation replicates. The authors should report the number of replicates for each setting and clarify whether results are summed or averaged. Also, reporting average TP and FP per replicate, with uncertainty intervals, would make the results easier to interpret.

Response:

Thank you for your comment. To make it more clear and avoid confusion, we have now revised panels (C) and (D) in figures 2 and 3 to show the true FDR at the nominal level of 0.1 to evaluate the FDR control of all methods (see updated figures (E)-(F) below and complete figure in the revised manuscript). In addition, we also added two figures showing the power to detect causal genes across varying numbers of underlying factors and phenotype heritability levels (see updated figures (C)-(D) below and complete figure in the revised manuscript).

Revised Fig 2C-D

Revised Fig 2E-F

 Revised Fig 3C-D

Revised Fig 3E-F

8. Since GIFT and MVIWAS are single-trait methods, the authors apply factor analysis to reduce multiple phenotypes into a smaller number of latent traits prior to analysis. However, this is only one possible strategy. An alternative and more standard approach is to apply these methods separately to each phenotype and then combine results, as what they did in real data application 2. The simulation would be strengthened by including results from both approaches, as factor analysis may introduce additional noise and potentially disadvantage single-trait methods.

Response:

Thank you for your suggestions. We have now included the alternative way of combining the individual phenotype using meta-analysis methods (i.e. Fisher’s method that combines p-values) for GIFT and MVIWAS methods (called GIFT Meta and MVIWAS Meta, respectively) as suggested in the main simulation results for comparison. We have updated the figures in the revised manuscript. In short, we found that for continuous outcomes the methods perform similarly well to the original GIFT Factor and MVIWAS Factor. For the mixed outcomes, there is a large increase in the gene detection rate, leading to higher power (see revised Fig 2,3 (C)-(D) above), but with severely inflated FDR (see revised Fig 2,3 (E)-(F) above). Also, their overall AUC is not as high as FM-GPT (see Fig 2,3 panel (A)-(B) in the revised manuscript).

9. The manuscript applies BFDR for Bayesian methods and the BH procedure for frequentist methods, using the same nominal threshold. However, these approaches control different quantities and are not directly comparable. Please clarify this distinction and discuss its implications for benchmarking.

Response:

We thank the reviewer for the comment. We agree that direct comparison between Bayesian and frequentist methods is inherently challenging because they quantify uncertainty using different inferential frameworks. FM-GPT is a Bayesian method that outputs posterior inclusion probabilities (PIP) and Bayesian false discovery rates (BFDR), whereas several competing methods produce p-values. Consequently, FM-GPT does not naturally provide p-values, and converting posterior probabilities into p-values would not be statistically meaningful. For the frequentist methods, we used BH-adjusted p-values to control the false discovery rate because FDR control is the most widely adopted criterion in high-dimensional genomic studies and is conceptually closest to the BFDR criterion used by FM-GPT [concept of BFDR first proposed by Newton 2004 in genomic context]. BH adjustment is approximate under correlated tests and that Bayesian and frequentist FDR measures are not identical. Our primary evaluation in simulations was based on AUC, which is rank-based and do not depend on the specific significance measure or cutoff used by each method (Fig 2,3 (A)-(B)). For a more comprehensive comparison, we additionally applied the Benjamini–Yekutieli (BY) procedure [Benjamini and Yekutieli (2001)], which provides FDR control under arbitrary dependence structures and is generally more robust to correlated p-values than the BH procedure. Furthermore, to avoid potential biases arising from the conversion of p-values to FDR-adjusted significance measures, we performed supplementary comparisons based on raw p-values and family-wise error rate (FWER) control. Table R4 show the comparison of power and true FDR control between our method vs. other frequentist methods in simulation scenario 1 (heritability of 3% and three true underlying factors, continuous only). Across all evaluation criteria, FM-GPT consistently identified a greater number of true causal genes while maintaining appropriate error control regardless than all the frequentist methods regardless of the different measures/cutoffs used. Among the frequentist approaches, BH- and BY-adjusted FDR cutoffs tended to perform best and most closely matched the BFDR-based results. These findings demonstrate that the superior performance of FM-GPT is not driven by the particular significance measure or thresholding strategy used, but rather reflects its improved ability to prioritize truly causal genes.

Table R4. Comparison of different p-value adjustment methods for frequentist methods with FM-GPT using BFDR on true FDR and power in the simulation scenario 1 (heritability of 3% and three true underlying factors, continuous only; nominal FDR=0.1).

True FDR:

| **Adjustment Type** | **GIFT Factor** | **GIFT Meta** | **mvIWAS Factor** | **mvIWAS Meta** | **FM-GPT** |
| --- | --- | --- | --- | --- | --- |
| **Raw P-Values** | 0.32 (0.01) | 0.26 (0.01) | 0.41 (0.01) | 0.37 (0.01) | X |
| **Bonferroni FWER Adjustment** | 0.07 (0.01) | 0.05 (0.01) | 0.09 (0.01) | 0.11 (0.01) | X |
| **BH FDR Adjustment** | 0.11 (0.01) | 0.08 (0.01) | 0.16 (0.01) | 0.16 (0.01) | X |
| **BY FDR Adjustment** | 0.06 (0.01) | 0.04 (0.00) | 0.07 (0.01) | 0.09 (0.01) | X |
| **Bayesian PIP** | X | X | X | X | 0.02 (0.01) |
| **Bayesian FDR** | X | X | X | X | 0.01 (0.01) |

Power:

| **Adjustment Type** | **GIFT Factor** | **GIFT Meta** | **mvIWAS Factor** | **mvIWAS Meta** | **FM-GPT** |
| --- | --- | --- | --- | --- | --- |
| **Raw P-Values** | 0.83 (0.01) | 0.88 (0.01) | 0.86 (0.01) | 0.90 (0.01) | X |
| **Bonferroni FWER Adjustment** | 0.74 (0.01) | 0.83 (0.01) | 0.79 (0.01) | 0.84 (0.01) | X |
| **BH FDR Adjustment** | 0.76 (0.01) | 0.84 (0.01) | 0.81 (0.01) | 0.85 (0.01) | X |
| **BY FDR Adjustment** | 0.71 (0.01) | 0.81 (0.01) | 0.77 (0.01) | 0.83 (0.01) | X |
| **Bayesian PIP** | X | X | X | X | 0.89 (0.01) |
| **Bayesian FDR** | X | X | X | X | 0.89 (0.01) |

10. It is unclear what loadings are plotted in Figure 5E. Each gene can target multiple phenotypes through multiple latent factors.

Response:

Thank you for your comment. You are correct. For visualization purposes only, we displayed the **dominant factor** for each gene, where the dominant factor was defined as the factor with the largest overall loading magnitude for that gene. The heatmap was then constructed using the loadings corresponding to this dominant factor. During model fitting, we specified the same number of latent factors for each genomic region based on an examination of the outcome correlation structure. This allowed us to apply the same procedure for identifying the dominant factor across all regions, providing a consistent basis for visualization. Importantly, this dominant-factor assignment was used only for visualization and does not affect model fitting or gene prioritization. We have revised the figure caption to clarify this distinction.

**Reviewer #3**: This manuscript proposes FM-GPT, a Bayesian fine-mapping framework for phenome-wide transcriptome-wide association studies that models genetically regulated gene expression effects on latent phenotypic factors, allowing joint analysis of multiple correlated traits across different types of outcomes. The authors develop a hierarchical Bayesian model with latent factors, gene–factor effects, factor loadings, and data-augmentation steps for non-Gaussian traits, implemented via Gibbs sampling. Through simulations, the authors show that the proposed method is more accurate in identifying true causal genes as compared to existing methods in various settings. The application of FM-GPT to real UKB and EHR data suggest FM-GPT can decrease the set of potentially causal genes.

Overall, the topic is timely and potentially useful for identifying causal genes across many correlated phenotypes, but several aspects of the simulation design and model assumptions require clarification or additional sensitivity analyses before the evidence fully supports the methodological claims.

*Major:*

1. The manuscript proposes both gene–factor pair tests and a global test across factors. Please clarify which test is used in the simulation evaluation of FDR and power. If both tests are central to the method, both should be evaluated separately, since they answer different inferential questions and may have different calibration properties.

Response:

We thank the reviewer for the comment. The global omnibus test was used in the simulation evaluation of FDR and power. To avoid confusion, we have now removed the factor-specific BFDR from the manuscript. In the revised version, we report only the omnibus gene-level BFDR (and this is what has been used in simulations), which aggregates evidence across all latent factors within a region and serves as the primary criterion for gene prioritization and statistical inference. Since the omnibus BFDR does not rely on alignment or comparability of latent factor indices across regions, it is more consistent with the intended interpretation of our region-specific latent factor framework. We have revised the Methods section accordingly and clarified that all reported discoveries are based on the omnibus gene-level BFDR.

2. The model uses a diagonal cross-study residual covariance matrix, \Sigma, which implicitly assumes independent residual variation across phenotypes or studies. This assumption may be problematic in the real application, where the phenotypes are measured in the same individuals and may share environmental, technical, or ascertainment-related residual correlations. The authors should clarify this modeling assumption and conduct sensitivity analyses to evaluate its impact in settings with correlated residual errors.

Response:

Thank you for your comment. We agree that assuming a diagonal cross-study residual covariance matrix may be restrictive in real applications, particularly when phenotypes are measured in the same individuals and may share environmental, technical, or ascertainment-related residual correlations. We have revised the manuscript to clarify this modeling assumption and additional sensitivity analyses to evaluate its potential impact. We have now included additional sensitivity analyses to simulate data under two forms of correlated residual errors: a compound-symmetric (CS) covariance structure and an AR(1) covariance structure, each evaluated across varying levels of residual correlation ($\rho$). The results, summarized in Table R7 and provided in the Supplementary Materials, show that FM-GPT remains robust across these settings. In particular, even under strong CS and AR(1) residual correlations, FM-GPT maintained high AUC and power while keeping FDR well controlled. These findings suggest that the diagonal residual covariance assumption has limited impact on gene prioritization performance in the scenarios considered.

Table R7. Simulation scenario 1 results (phenotype heritability 5% and true number of factors equal to 3, continuous) for different covariance matrix structures

| **Correlation Type** | **Correlation Coefficient** | **AUC** | **FDR** | **Power** |
| --- | --- | --- | --- | --- |
| **Compound Symmetry (CS)** | **0.15** | 1.00 (0.00) | 0.00 (0.00) | 0.98 (0.01) |
|  | **0.33** | 1.00 (0.00) | 0.00 (0.00) | 0.93 (0.02) |
|  | **0.66** | 0.98 (0.01) | 0.00 (0.00) | 0.91 (0.03) |
| **AR(1)** | **0.15** | 0.99 (0.00) | 0.02 (0.01) | 0.98 (0.01) |
|  | **0.33** | 1.00 (0.00) | 0.00 (0.00) | 1.00 (0.01) |
|  | **0.66** | 0.99 (0.00) | 0.01 (0.01) | 0.96 (0.02) |

3. Clarification of the simulation genotype and genomic-region design is needed. The simulation design is not sufficiently clear. In the supplementary note, the authors simulated 10 independent regions, each containing 10 genes, with each gene having 10 cis-SNPs explaining 10% of the variation in expression. It also describes two genotype-generation strategies: a synthetic LD structure with correlation 0.66 among cis-SNPs within a gene and 0.33 across genes, and an alternative strategy using a reference LD matrix, such as 1000 Genomes. Please clarify which simulation results correspond to which genotype-generation strategy. If real LD was used, how were the 10 genomic regions selected? Were they based on real genotypes and real gene annotations? How were the 10 genes within each region selected, and do they reflect realistic local LD and gene-density structures? The assumed synthetic correlations of 0.66 within genes and 0.33 across genes appear simplified and may not represent realistic cis-regulatory architecture.

Response:

Thank you for this important comment. We agree that the original description of the genotype and genomic-region simulation design was not sufficiently clear. We have revised the Supplementary Methods to explicitly clarify the genotype-generation strategies used in the simulations and which results correspond to which setting. In the original manuscript, the main simulation results were based on synthetically generated genotype data with a prespecified LD structure, where cis-SNPs within the same gene had correlation 0.66 and SNPs across genes within the same region had correlation 0.33. This design was intended to provide a controlled benchmark in which the strength of within-gene and between-gene LD could be specified directly. We agree with the reviewer that this structure is simplified and may not fully capture realistic cis-regulatory architecture. To address this concern, we have now added additional simulation results using more realistic LD based on 1000 Genome reference. Specifically, we considered a reference LD matrix directly from 1000 Genomes haplotypes (we’ve done this sensitivity analysis in the last version but did not include the results in the main text) and then used HAPGEN2 to simulate the genotype data. These analyses were designed to assess whether the performance of FM-GPT was sensitive to the simplified synthetic LD structure used in the main simulations. The genomic regions were selected from chr 15 and required to have at least 10 protein-coding genes based on GENCODE. Haplotypes were extracted from the 1000 Genomes Phase 3 IMPUTE2-formatted reference panel for chr 15, and the genotypes were then simulated using HAPGEN2 (details described in Supplementary methods). We have added these details in the revised Supplementary methods and added the results to the Supplementary results in addition to the synthetic results in the main text (Fig S1-S2, also see below). A representative comparison for simulation scenario 1 was shown in Table R8 below. Overall, the results were broadly consistent across LD and genotype-generation strategies. FM-GPT maintained high AUC and power under both synthetic and reference-based LD structures and for both genotype data generation procedures. The true FDR remained close to the nominal 0.1 level across the reference-based simulations, with slight inflation in mixed with 5% heritability case. These results suggest that the main conclusions are not driven by the simplified synthetic LD structure and remain largely robust under more realistic LD and genotype-generation procedures.

Table R8. Simulation scenario 1 results (phenotype heritability 5% and true number of factors equal to 3, continuous) with different genotype simulation designs (FDR nominal level of 0.1)

| **LD structure** | **Genotype data generation** | **AUC** | **FDR** | **Power** |
| --- | --- | --- | --- | --- |
| **Synthetic LD**  (within=0.66, between=0.33) | **latent normal** | 1.00 (0.00) | 0.01 (0.01) | 0.98 (0.01) |
| **Reference LD (chr15 from 1000 genome)** | **HAPGEN2** | 0.99 (0.01) | 0.11 (0.02) | 0.94 (0.02) |

Added Fig S1. Simulation I homogeneous results using reference LD + HAPGEN2 to generate genotype

Added Fig S2. Simulation 2 heterogeneous results using reference LD + HAPGEN2 to generate genotype

4. Please clarify the definition and number of local SNPs per gene. The author state that each gene includes 10 cis-SNPs, but it is unclear whether these are the only local SNPs considered for each gene or whether they are causal SNPs selected from a larger cis-window. Please clarify the total number of local SNPs per gene and the number of SNPs.

Response:

Thank you for your comment. We have revised the main text and Supplementary methods to clarify the definition and number of local SNPs used in the simulation. In our simulation design, each genomic region contains 10 genes, and each gene is assigned exactly 10 local cis-SNPs. These 10 SNPs represent the full set of local SNPs considered for that gene. For expression generation, all 10 cis-SNPs assigned to a gene are allowed to contribute to that gene’s genetically regulated expression. We did not impose a separate causal/non-causal SNP indicator at the SNP-expression level. Instead, SNP-expression effect sizes were generated from a Gaussian distribution, so some effects may be very small and contribute negligibly in practice. This design is relatively simpler but appropriate for our simulation objective because FM-GPT aims to prioritize causal genes rather than identifying causal SNPs within each gene. This design is also consistent with the TWAS literature (*12-14*), where genetically regulated expression is commonly modeled as a weighted combination of multiple local cis-SNPs within a gene-specific prediction window, rather than as the effect of a single causal SNP. The SNP sets were specified to be non-overlapping across genes for simplicity, so each cis-SNP was assigned to one gene only. We have clarified these details in the revised manuscript and Supplement.

5. Please clarify whether the true gene-expression coefficients, \gamma, were used as input to FM-GPT or whether they were estimated from the reference eQTL data to mimic the real procedure of TWAS. If true \gamma values were used, this would give FM-GPT an unrealistic advantage over competing methods such as GIFT and should be avoided or clearly separated as an oracle analysis.

Response:

Thank you for your comment. We agree that this is an important distinction to make and would unfairly bias our method. We did not provide the true $\gamma$ values as input to FM-GPT. In the simulations, we randomly generate $\gamma$ according to the normal distribution. $\hat{\gamma}$ was estimated using our full Bayesian model for FM-GPT, other methods GIFT and mvIWAS directly used PrediXcan estimated $\hat{\gamma}$ based on elastic net regularization. This ensures a fair comparison between all comparative methods. We have further polished the text in the revised manuscript and Supplement to make it more clear.

6. The simulations vary the number of phenotypic factors across 1, 3, and 5, and the Gibbs sampler includes a step for updating the number of factors by adding or removing factors based on near-zero loading vectors. However, it is unclear whether the number of factors used in the simulation analyses was fixed to the true value, estimated by the model, or selected by the user. If the true number of factors was supplied, this should be stated clearly, and the authors should provide a sensitivity analysis evaluating FDR and power under factor-number misspecification.

Response:

Thank you for this important comment. We have revised the main text and Supplementary Methods to clarify how the number of latent factors was handled in the simulation analyses. In the main simulations, the true number of factors was not supplied to the model. Instead, FM-GPT used an adaptive factor-update step (see Supplement Methods S5 and S6) to add or remove factors based on near-zero loading vectors. Thus, the number of factors used for inference was estimated by the model rather than fixed to the true data-generating value.

To further address the reviewer’s concern, we added a sensitivity analysis evaluating the impact of factor-number misspecification on AUC, true FDR, and power (Table R9). In this analysis, data were generated with 3 true underlying factors. We compared four settings: (i) the default model-based factor estimation procedure, (ii) fitting too few factors (1), (iii) fitting moderately too many factors (5), and (iv) fitting substantially too many factors (10).

As shown in Table R9, the adaptive model-based procedure recovered a median of three factors and achieved strong performance, with high AUC and power and well-controlled true FDR. In contrast, fixing the number of factors to an incorrect value reduced performance. Fitting only one factor led to lower AUC and power and inflated FDR, likely because the model could not adequately capture the true multi-factor phenotypic structure. Similarly, fitting too many factors, particularly 10 factors, led to reduced AUC and power and increased FDR, likely due to over-parameterization and unstable allocation of gene effects across redundant factors. These results indicate that factor-number misspecification can adversely affect performance, supporting the use of the adaptive factor-estimation procedure in FM-GPT.

Table R9. Simulation scenario 1 results (true number of factors equal to 3) of FM-GPT when the number of factors are mis-specified

| **#Factors Fit** | **AUC** | **FDR** | **Power** |
| --- | --- | --- | --- |
| **Model-based estimation (median = 3)** | 0.99 (0.01) | 0.00 (0.00) | 0.99 (0.01) |
| **1** | 0.88 (0.02) | 0.31 (0.05) | 0.52 (0.05) |
| **5** | 0.86 (0.02) | 0.44 (0.04) | 0.64 (0.05) |
| **10** | 0.74 (0.02) | 0.58 (0.04) | 0.46 (0.05) |

We also added a sensitivity analysis assessing how well FM-GPT recovers the true number of factors across simulation settings (Table R10). Specifically, we evaluated the proportion of simulation replicates in which the true number of generated factors was contained within the model’s 95% highest-density interval for the inferred number of factors. Across the settings considered, FM-GPT accurately captured the true number of factors, further supporting the robustness of the adaptive factor-selection procedure.

Table R10. Factor recovery performance of FM-GPT

| **Scenario** | **Outcome data type** | **One Underlying Factor** | **Three Underlying Factors** | **Five Underlying Factors** |
| --- | --- | --- | --- | --- |
| 1. Homogeneous | Continuous | 0.95 (0.01) | 0.97 (0.01) | 0.94 (0.01) |
|  |  | 0.95 (0.01) | 0.97 (0.01) | 0.94 (0.01) |
|  |  | 0.95 (0.01) | 0.95 (0.01) | 0.95 (0.01) |
|  | Mixed | 0.91 (0.01) | 0.89 (0.01) | 0.57 (0.02) |
|  |  | 0.93 (0.01) | 0.81 (0.01) | 0.57 (0.02) |
|  |  | 0.91 (0.01) | 0.82 (0.01) | 0.53 (0.02) |
| 2. Heterogeneous | Continuous | 0.99 (0.00) | 0.97 (0.01) | 0.94 (0.01) |
|  |  | 0.97 (0.01) | 0.97 (0.01) | 0.92 (0.01) |
|  |  | 0.92 (0.01) | 0.96 (0.01) | 0.92 (0.01) |
|  | Mixed | 0.98 (0.00) | 0.95 (0.01) | 0.74 (0.01) |
|  |  | 0.89 (0.01) | 0.91 (0.01) | 0.73 (0.01) |
|  |  | 0.89 (0.01) | 0.91 (0.01) | 0.60 (0.02) |

7. In the real-data analyses, the manuscript should explain how users should determine the number of latent factors. Are results robust to different choices? The authors should report how the findings change when different numbers of factors are used, or provide a principled selection procedure and diagnostic guidance for users.

Response:

Thank you for this important comment. As we showed in the last question above, forcing the model to include too few or too many factors will worsen the performance in simulation (Table R9). We have revised the manuscript to clarify how the number of latent factors was handled in each real-data application and have added additional guidance for users in the Supplementary Methods.

In real-data application 1, we allowed FM-GPT to estimate the number of factors using the adaptive factor-update step and trace plot described in the Supplementary Methods. In real-data application 2, we examined the outcome correlation structure and scree plot before model fitting and specified three latent factors, which reflected the major correlation patterns observed among the phenotypes. We have clarified these choices in the corresponding real-data analysis sections of the revised manuscript.

To provide practical guidance for users, we added a new Supplementary Section S4, “Guidance for Determining the Number of Factors”. Users may allow FM-GPT to adaptively estimate the number of factors during the Gibbs sampler by using the factor birth–death/update step described in the Methods. The adaptation parameters can be tuned to control how aggressively redundant factors are removed or additional factors are explored. Additionally, FM-GPT provides several diagnostic plots (included in the tutorial) to help users assess the appropriate number of factors. First, users can examine the trace plot of the number of factors retained across Gibbs sampling iterations and evaluate the convergence. Second, users can also inspect the phenotype correlation matrix to have some idea of how many factors exist in the latent structure and then examine the eigenvalues of the phenotype correlation matrix in a scree plot, and identify an elbow point for a reasonable number of factors, as in other factor or clustering analysis methods. Because FM-GPT is applied separately to each genomic region, the effective number of factors may differ across regions. Thus, the phenotypic correlation matrix and scree plot should be used as exploratory guidance rather than definitive criteria; we recommend combining them with region-specific trace plots and loading-matrix diagnostics to select the number of factors. Users can also examine the posterior mean loading matrix to identify potential redundant or weak factors, defined as factors with uniformly small loadings relative to the others. Such factors may be interpreted cautiously or excluded from downstream visualization. We have included an example of these diagnostics in the Supplementary methods and updated tutorial on Github.

In practice, specifying a slightly larger number of factors may be acceptable because the gamma process shrinkage prior discourages redundant factors by shrinking weak loadings toward zero. However, the number of factors should not be chosen arbitrarily, and we now recommend that users combine adaptive estimation, outcome-correlation diagnostics, loading-matrix inspection, and sensitivity analyses when applying FM-GPT to new real-data settings.

8. The proposed augmentation strategy for binary outcomes should be discussed in the context of rare diseases and severe case-control imbalance. The UK Biobank phenotype-processing pipeline filters binary phenotypes to those with at least 1% cases, but it is unclear whether the method remains calibrated and powerful for rarer traits. The authors should either provide simulations for rare binary traits or clearly state the limitations of the current implementation.

Response:

Thank you for this important comment. We agree that the performance of the proposed augmentation strategy for binary outcomes should be evaluated under rare-disease and severe case-control imbalance settings. We have therefore added a sensitivity simulation study in which the binary outcome case rate was varied across approximately 1%, 5%, 10%, and 50%. In each setting, the phenotypic heritability was set to 5% and the true number of underlying factors was set to 3. The 50% case-rate setting corresponds to the more balanced binary-outcome scenario considered in the original manuscript and is included here as a benchmark.

As shown in Table R9, FM-GPT maintained well-controlled FDR across all case-rate settings. However, performance was affected by extreme case-control imbalance. In the rare binary trait setting with approximately 1% cases, FM-GPT achieved an AUC of 0.87 and true FDR of 0.01, but power decreased to 0.47. In contrast, for case rates of 5% and 10%, FM-GPT achieved substantially higher AUCs of 0.98 and 0.97, respectively, with power of 0.85 in both settings and true FDR remaining below the nominal level. Under the balanced 50% case-rate setting, FM-GPT achieved an AUC of 0.99 and power of 0.94, with true FDR of 0.03.

These results suggest that FM-GPT remains well calibrated in terms of FDR control under rare binary outcome settings, but statistical power can be substantially reduced when the case rate is extremely low, particularly around 1%. We have added these results to the Supplementary Materials and revised the Discussion to note that the current implementation may have limited power for very rare binary traits or highly imbalanced case-control designs. Accordingly, in real-data applications, we recommend applying appropriate phenotype filters using 1% case-rate threshold (main text real example 2) used in the UK Biobank analysis, and interpreting results for rare binary outcomes with caution.

Table R11. Simulation scenario 1 results (true number of factors equal to 3, phenotypic heritability of 5%) of FM-GPT with varying proportion of cases in binary traits

| **Proportion of cases in binary traits** | **AUC** | **FDR** | **Power** |
| --- | --- | --- | --- |
| **1%** | 0.87 (0.02) | 0.01 (0.01) | 0.47 (0.05) |
| **5%** | 0.98 (0.01) | 0.04 (0.01) | 0.85 (0.03) |
| **10%** | 0.97 (0.01) | 0.03 (0.01) | 0.85 (0.03) |
| **50%** | 0.99 (0.00) | 0.03 (0.01) | 0.94 (0.02) |

9. The codes for reproducing simulation results and simulation data are not available via the github link provided by the authors. Please provide R scripts or a reproducible workflow to regenerate the simulation results and real-data analyses.

Response:

Thank you for your comment. We have now included a reproducible example of the simulation, as well as the R scripts on how we regenerate the simulation and real data analyses on the github page.

*Minor*:

1. The dimensions in Equation 3 appear inconsistent. \tilde{Y}’ is q by n2, \Lambda is q by m but F_i is m by 1.

Response:

Thank you for your comment. We have adjusted the manuscript to fix this typo so that the dimensions now match.

2. In panels C and D of Figures 2 and 3, it would be helpful to directly annotate the observed FDR values, or add reference lines/labels, so readers can more easily assess calibration.

Response:

Thank you for your comment. We have completely redone panels (C) and (D) of figures 2 and 3 so that it is more clear. We now show directly the true FDR values to evaluate how well each method controls FDR at the nominal level of 0.1.

3. The R package would benefit from a more complete tutorial, including a minimal working example, required input formats, parameter choices, interpretation of gene–factor and global-test outputs, and guidance on choosing the number of factors.

Thank you for your comment. We now include a full tutorial showing expected inputs to the model and also how to analyze the output. We also include as part of the tutorial how the user can follow the principled diagnostic procedure to best select the number of factors.

4. The gene labels at the bottom of Figure 5C are not visible.

Thank you for your comment. We have adjusted the figure so that the gene labels are legible.

Reference:

1.	S.-H. Wang, R. Bai, H.-H. Huang, Two-step mixed-type multivariate Bayesian sparse variable selection with shrinkage priors. *Electronic Journal of Statistics* **19**, 397-457 (2025).

2.	N. G. Polson, J. G. Scott, J. Windle, Bayesian inference for logistic models using Pólya–Gamma latent variables. *Journal of the American statistical Association* **108**, 1339-1349 (2013).

3.	M. A. Newton, A. Noueiry, D. Sarkar, P. Ahlquist, Detecting differential gene expression with a semiparametric hierarchical mixture method. *Biostatistics* **5**, 155-176 (2004).

4.	Y. Benjamini, D. Yekutieli, The control of the false discovery rate in multiple testing under dependency. *Annals of statistics*, 1165-1188 (2001).

5.	Y. Liu, J. Xie, Cauchy combination test: a powerful test with analytic p-value calculation under arbitrary dependency structures. *Journal of the American Statistical Association* **115**, 393-402 (2020).

6.	J. Bernardo *et al.*, Bayesian factor regression models in the “large p, small n” paradigm. *Bayesian statistics* **7**, 733-742 (2003).

7.	C. M. Carvalho *et al.*, High-Dimensional Sparse Factor Modeling: Applications in Gene Expression Genomics. *J Am Stat Assoc* **103**, 1438-1456 (2008).

8.	A. Bhattacharya, D. B. Dunson, Sparse Bayesian infinite factor models. *Biometrika* **98**, 291-306 (2011).

9.	E. A. Boyle, Y. I. Li, J. K. Pritchard, An Expanded View of Complex Traits: From Polygenic to Omnigenic. *Cell* **169**, 1177-1186 (2017).

10.	X. Liu, Y. I. Li, J. K. Pritchard, Trans Effects on Gene Expression Can Drive Omnigenic Inheritance. *Cell* **177**, 1022-1034.e1026 (2019).

11.	K. Watanabe *et al.*, A global overview of pleiotropy and genetic architecture in complex traits. *Nat Genet* **51**, 1339-1348 (2019).

12.	E. R. Gamazon *et al.*, A gene-based association method for mapping traits using reference transcriptome data. *Nat Genet* **47**, 1091-1098 (2015).

13.	A. Gusev *et al.*, Integrative approaches for large-scale transcriptome-wide association studies. *Nat Genet* **48**, 245-252 (2016).

14.	A. N. Barbeira *et al.*, Exploring the phenotypic consequences of tissue specific gene expression variation inferred from GWAS summary statistics. *Nat Commun* **9**, 1825 (2018).
