# Supplementary material for: FM-GPT: Bayesian fine mapping for phenome-wide transcriptome-wide association studies
Source: PLoS Genet. 2026 Sep 8;22(9):e1012126. doi: 10.1371/journal.pgen.1012126 (PMC13581217; doi:10.1371/journal.pgen.1012126)
Supplement: Supplementary file 8 [file pgen.1012126.s026.docx]

**Response Letter (Round 2)**

We want to thank all reviewers for taking time to review our manuscript and provide valuable comments and suggestions to improve the quality of our manuscript. We have taken the advice from reviewers to carefully revise the manuscript (revised parts red highlighted). Below please also find a point-by-point response to reviewers' comments for this 2^nd^ round:

**Reviewer #1**: The authors have carefully addressed all my questions. Thanks!

**Reviewer #2**: The authors have addressed most of my comments. I recommend acceptance after minor revision. See details of my remaining comments below.

1. To address my previous comment 4, the authors proposed an omnibus gene-level BFDR. In the revised definition, the discovery indicator is written as I{PIP_j < z}, which appears inconsistent with selecting high PIP genes. Please double check.

Thank you for your comment and attention to detail. This is a typo and the correct definition should be $d_{j}\left( z \right)=I\{1\boldsymbol{-}PIP_{j}<z\}$. We have accordingly updated the formula in the main text.

2. The revised Figure 1B is clearer and now includes region-specific modeling, priors, and mixed-outcome augmentation. One minor issue in the legend: "Z^R and Xhat^G are the genotype estimated GReX data from GWAS". This sentence appears unclear and possibly includes notation errors. Please double check and revise.

Thank you for your comment. This is a typo, we have now fixed the notation and edited the caption: $Z^{G}$ and  $\hat{X}^{G}$ are the genotype and estimated GReX data from GWAS.

3. For Figure 5E, the authors mentioned in the response that they plot the "dominant factor for each gene," but factor loadings are phenotype-by-factor quantities, not gene-specific unless combined with gene-factor effects. The definition "largest overall loading magnitude for that gene" is still ambiguous. I suggest explicitly defining whether the dominant factor is chosen by β_jl, the factor-specific PIP, the product of gene-factor effect and phenotype loading magnitude, only the norm of the factor-loading vector, or other ways. These choices have different interpretations. Please also consider clarifying in the legend that the heatmap is a visualization summary only and does not represent the full gene-phenotype association structure, since genes may act through multiple latent factors.

Thank you for your comment. Yes, the loadings represent phenotype-by-factor quantities. However, FM-GPT was fit separately within each gene-centered genomic region, so each genomic region/gene has its own loading matrix (each row of heatmap in Fig 5E is factor loadings obtained separately for each gene), implying the gene-guided dimension reduction characteristics of our method. For each gene, we selected the dominating factor as the factor with the largest factor-loading vector norm, i.e. $l_{dom}=argmax_{l}\left| \left| \vec{\lambda_{l}} \right| \right|_{2}$, where $\vec{\lambda_{l}}=(\lambda_{1l},\ldots,\lambda_{ql})$. We combined these separately estimated dominant loading profiles in one heatmap to compare the phenotypic loading patterns across genes. Similar loading patterns across genes may indicate that multiple genetic regions influence overlapping sets of phenotypes, consistent with a pleiotropic architecture involving shared biological or regulatory pathways. We have now taken your advice to revise the main text and Fig 5E caption to avoid ambiguity:

(E) Heatmap of identified putative causal genes and the loadings of phenotypes they target at in the dominating factor with the largest factor-loading vector norm. Note that the heatmap is a visual summary only and does not represent the full set of factors by which a gene may be associated with the phenotypes.

**Reviewer #3**: The authors have addressed my major concerns. There are some minor comments.
1. The reference to LDtect is wrong( ref 21). This should refer to PMID: 26395773

Thank you for your comment. We have updated the reference as you suggest.

2. Typo in Section title: 'FM-GPT identifies genes that influence multiple medial conditions derived from EHR data' should read 'multiple medical conditions.'

Thank you for your attention to detail. We have fixed this typo and read the text again to catch any additional typos.

3. In the last paragraph of Introduction, the phrase “In an brain-wide genetic analysis” should be changed to “In a brain-wide genetic analysis.”

Thank you for the correction. We have fixed this typo.
