# Supplementary material for: FM-GPT: Bayesian fine mapping for phenome-wide transcriptome-wide association studies
Source: PLoS Genet. 2026 Sep 8;22(9):e1012126. doi: 10.1371/journal.pgen.1012126 (PMC13581217; doi:10.1371/journal.pgen.1012126)
Supplement: Peer review history

# Peer review: Decision Letter 0

**DOI:** 10.1371/journal.pgen.1012126.r001

**Authors:** Lin S. Chen, Xiaofeng Zhu

27 Apr 2026

PGENETICS-D-26-00334

FM-GPT: Bayesian fine mapping for phenome-wide transcriptome-wide association studies

PLOS Genetics

Dear Dr. Ma,

Thank you for submitting your manuscript to PLOS Genetics. After careful consideration, we feel that it has merit but does not fully meet PLOS Genetics's publication criteria as it currently stands. Therefore, we invite you to submit a revised version of the manuscript that addresses the points raised during the review process.

Please submit your revised manuscript by Jun 26 2026 11:59PM. If you will need more time than this to complete your revisions, please reply to this message or contact the journal office at plosgenetics@plos.org. When you're ready to submit your revision, log on to https://www.editorialmanager.com/pgenetics/ and select the 'Submissions Needing Revision' folder to locate your manuscript file.

Please include the following items when submitting your revised manuscript:

* A letter that responds to each point raised by the editor and reviewer(s). You should upload this letter as a separate file labeled 'Response to Reviewers'. This file does not need to include responses to any formatting updates and technical items listed in the 'Journal Requirements' section below.

* A marked-up copy of your manuscript that highlights changes made to the original version. You should upload this as a separate file labeled 'Revised Manuscript with Track Changes'.

* An unmarked version of your revised paper without tracked changes. You should upload this as a separate file labeled 'Manuscript'.

If you would like to make changes to your financial disclosure, competing interests statement, or data availability statement, please make these updates within the submission form at the time of resubmission. Guidelines for resubmitting your figure files are available below the reviewer comments at the end of this letter.

We look forward to receiving your revised manuscript.

Kind regards,

Lin S. Chen, Ph.D.

Academic Editor

PLOS Genetics

Xiaofeng Zhu

Section Editor

PLOS Genetics

Aimée Dudley

Editor-in-Chief

PLOS Genetics

Anne Goriely

Editor-in-Chief

PLOS Genetics

**Journal Requirements:**

1) Please upload all main figures as separate Figure files in .tif or .eps format. For more information about how to convert and format your figure files please see our guidelines:

https://journals.plos.org/plosgenetics/s/figures

2) We have noticed that you have uploaded Supporting Information files, but you have not included a list of legends. Please add a full list of legends for your Supporting Information files after the references list.

**Reviewers' comments:**

Reviewer's Responses to Questions

**Comments to the Authors:**

**Please note here if the review is uploaded as an attachment.**

Reviewer #1: The authors proposed a new TWAS fine mapping method for multiple traits of possibly mixed types with a fully Bayesian approach. Numerical studies with both simulated and UKB data show both the feasibility and some advantages of the proposed method over several existing methods.

Major comments.

1. A main promise of the proposed method is its capability to handle multiple types of traits, for which the major technical approach is a normal distribution-based latent factor analysis of the multiple traits to be analyzed. The following questions need to be clarified.

1) For a discrete trait, how/why is the proposed normal-based factor analysis reasonable or justified? To be concrete, let’s consider a binary trait (among multiple traits): then the corresponding LHS of eq (3) is a binary random variable (RV) (after subtracting a constant, then divided by another random variable), but the RHD is a normal random variable; how can such a linear transformation is possible (to transform a binary RV to a normal RV)?

2) Since the second real data application involves many and mostly binary traits while the given simulation set-ups did not seem to cover such a case, having some simulation results under a set-up mimicking such a case would help.

3) Given that Eq (3) is the main technical innovation, its discussion is too sketchy. For example, it is not clear what the purpose of the proposed transformation is and how it is done for a discrete RV. It did not help much even after I studied Section 3 and the cited reference [57].

4) Based on the discussion of Eq (3), it seems to me that the proposed method can only handle two types of non-normal traits: binary (Bernoulli) and negative binomial-based count data. It would be better to state it out clearly what types of continuous and discrete traits are covered because “mixed types of traits” can be more general.

5) It is stated: “Unlike standard unsupervised factor analysis which assumes a single global set of latent factors, our model allows the latent factors to be specific to each genomic region under study.” Where and how?

6) Since applying (sparse) PCA seems to be straightforward for GWAS/TWAS of multiple traits, how would the proposed method differ from PCA/sPCA?

I think that one downside of the proposed method, as one based on (s)PCA, is the loss of specificity: even with sparse leadings, one may not know which associations are for one specific trait. Some discussion may be needed.

2. Some proposed methods output p-values (while the proposed is based on FDR); for comparison, the former were transformed to FDR, which is not easy (and is approximate with errors) with correlated p-values. Thus, it may not be fair to compare the methods in that way; one may ask why not compare the methods based on p-values or family-wise type I error control? While I feel that the authors’ comparison might be useful, such limitation has to be acknowledged.

BTW, the authors proposed combining the results of single-trait analyses using Fisher’s method, which, strictly speaking, may not be valid because those traits/p-values are not independent.

3. The quality of some figures is not high: for example, I cannot read the numbers in Figs 2&3, especially panels (C) and (D). Please give concrete results that the proposed method can control FDR effectively.

Reviewer #2: In this manuscript, the authors introduced a Bayesian fine-mapping framework that jointly models multiple correlated phenotypes and prioritizes genes through a GreX-informed factor analysis. The framework is flexible and addresses an important problem in large-scale phenome-wide TWAS analysis. However, several aspects of the methodology, interpretation, and evaluation require further clarification. I recommend major revision. Detailed comments are provided below.

1. The manuscript frequently refers to genes identified by fine-mapping methods as "causal". TWAS-based approaches detect associations between genetically predicted expression and traits, which may not be causal relationships. I recommend consistently using more accurate language, such as "putatively causal" or "prioritized genes".

2. The manuscript highlights "gene-guided dimension reduction" as a key methodological innovation. This is mainly described at a conceptual level but is not sufficiently clarified in terms of the model formulation. From Equation (2), it appears that latent factors are modeled as linear combinations of GReX, which constrains the factor space to lie within the span of predicted gene expression. This is a strong modeling assumption with important implications. The authors should clarify how gene expression "guides" the factor analysis, and how this approach relates to existing frameworks such as supervised factor models.

3. The model assumes region-specific latent factors, such that each genomic region has its own latent factor structure. This provides flexibility in modeling heterogeneity, but it makes the factors identified in different regions not directly comparable. It is not clear how the inferred factors are interpreted biologically and how "shared mechanisms" across regions are defined. Please clarify.

4. In Methods subsection 4 (page 12-13), the factor-specific BFDR is defined by aggregating PIPs across all genes from all genomic regions for a given factor index l. However, factor l in different regions may correspond to different latent structures. Please clarify how factor indices are aligned across regions, or alternatively restrict BFDR calculations to be region-specific, that is, indexed by both l and t. If only the omnibus gene-level BFDR is used in practice, this should be explicitly stated.

5. Latent factor models are inherently non-identifiable due to rotation and sign invariance. While sparsity constraints in priors partially mitigate this issue, they do not fully resolve it. The orientation and sign of inferred factors are not uniquely defined, and the biological interpretation may depend on arbitrary choices. I suggest the authors to assess the stability of inferred factors. In particular, they can repeat model fitting with different random seeds and initializations, examine whether selected genes and factor loading patterns are stable, and report concordance of inferred factors across runs. For real data applications, they can also perform bootstrap to assess whether the reported phenotype axes, such as the immune-metabolic axis, are reproducible.

6. Figure 1A can be improved by clarifying key model structures. In particular, the relationship between GReX, latent factors F, and phenotypes Y is ambiguous, and may be misinterpreted as standard unsupervised factor analysis. The figure presents the method as a sequential pipeline, whereas the model is actually jointly estimated. Also, key aspects such as region-specific modeling and sparsity-inducing priors are not illustrated.

7. In Figures 2C–D and 3C–D, the reported numbers of TP and FP appear to be aggregated over many simulation replicates. The authors should report the number of replicates for each setting and clarify whether results are summed or averaged. Also, reporting average TP and FP per replicate, with uncertainty intervals, would make the results easier to interpret.

8. Since GIFT and MVIWAS are single-trait methods, the authors apply factor analysis to reduce multiple phenotypes into a smaller number of latent traits prior to analysis. However, this is only one possible strategy. An alternative and more standard approach is to apply these methods separately to each phenotype and then combine results, as what they did in real data application 2. The simulation would be strengthened by including results from both approaches, as factor analysis may introduce additional noise and potentially disadvantage single-trait methods.

9. The manuscript applies BFDR for Bayesian methods and the BH procedure for frequentist methods, using the same nominal threshold. However, these approaches control different quantities and are not directly comparable. Please clarify this distinction and discuss its implications for benchmarking.

10. It is unclear what loadings are plotted in Figure 5E. Each gene can target multiple phenotypes through multiple latent factors.

Reviewer #3: This manuscript proposes FM-GPT, a Bayesian fine-mapping framework for phenome-wide transcriptome-wide association studies that models genetically regulated gene expression effects on latent phenotypic factors, allowing joint analysis of multiple correlated traits across different types of outcomes. The authors develop a hierarchical Bayesian model with latent factors, gene–factor effects, factor loadings, and data-augmentation steps for non-Gaussian traits, implemented via Gibbs sampling. Through simulations, the authors show that the proposed method is more accurate in identifying true causal genes as compared to existing methods in various settings. The application of FM-GPT to real UKB and EHR data suggest FM-GPT can decrease the set of potentially causal genes.

Overall, the topic is timely and potentially useful for identifying causal genes across many correlated phenotypes, but several aspects of the simulation design and model assumptions require clarification or additional sensitivity analyses before the evidence fully supports the methodological claims.

1. The manuscript proposes both gene–factor pair tests and a global test across factors. Please clarify which test is used in the simulation evaluation of FDR and power. If both tests are central to the method, both should be evaluated separately, since they answer different inferential questions and may have different calibration properties.

2. The model uses a diagonal cross-study residual covariance matrix, \Sigma, which implicitly assumes independent residual variation across phenotypes or studies. This assumption may be problematic in the real application, where the phenotypes are measured in the same individuals and may share environmental, technical, or ascertainment-related residual correlations. The authors should clarify this modeling assumption and conduct sensitivity analyses to evaluate its impact in settings with correlated residual errors.

3. Clarification of the simulation genotype and genomic-region design is needed. The simulation design is not sufficiently clear. In the supplementary note, the authors simulated 10 independent regions, each containing 10 genes, with each gene having 10 cis-SNPs explaining 10% of the variation in expression. It also describes two genotype-generation strategies: a synthetic LD structure with correlation 0.66 among cis-SNPs within a gene and 0.33 across genes, and an alternative strategy using a reference LD matrix, such as 1000 Genomes. Please clarify which simulation results correspond to which genotype-generation strategy. If real LD was used, how were the 10 genomic regions selected? Were they based on real genotypes and real gene annotations? How were the 10 genes within each region selected, and do they reflect realistic local LD and gene-density structures? The assumed synthetic correlations of 0.66 within genes and 0.33 across genes appear simplified and may not represent realistic cis-regulatory architecture.

4. Please clarify the definition and number of local SNPs per gene. The author state that each gene includes 10 cis-SNPs, but it is unclear whether these are the only local SNPs considered for each gene or whether they are causal SNPs selected from a larger cis-window. Please clarify the total number of local SNPs per gene and the number of SNPs.

5. Please clarify whether the true gene-expression coefficients, \gamma, were used as input to FM-GPT or whether they were estimated from the reference eQTL data to mimic the real procedure of TWAS. If true \gamma values were used, this would give FM-GPT an unrealistic advantage over competing methods such as GIFT and should be avoided or clearly separated as an oracle analysis.

6. The simulations vary the number of phenotypic factors across 1, 3, and 5, and the Gibbs sampler includes a step for updating the number of factors by adding or removing factors based on near-zero loading vectors. However, it is unclear whether the number of factors used in the simulation analyses was fixed to the true value, estimated by the model, or selected by the user. If the true number of factors was supplied, this should be stated clearly, and the authors should provide a sensitivity analysis evaluating FDR and power under factor-number misspecification.

7. In the real-data analyses, the manuscript should explain how users should determine the number of latent factors. Are results robust to different choices? The authors should report how the findings change when different numbers of factors are used, or provide a principled selection procedure and diagnostic guidance for users.

8. The proposed augmentation strategy for binary outcomes should be discussed in the context of rare diseases and severe case-control imbalance. The UK Biobank phenotype-processing pipeline filters binary phenotypes to those with at least 1% cases, but it is unclear whether the method remains calibrated and powerful for rarer traits. The authors should either provide simulations for rare binary traits or clearly state the limitations of the current implementation.

9. The codes for reproducing simulation results and simulation data are not available via the github link provided by the authors. Please provide R scripts or a reproducible workflow to regenerate the simulation results and real-data analyses.

Minor:

1. The dimensions in Equation 3 appear inconsistent. \tilde{Y}’ is q by n2, \Lambda is q by m but F_i is m by 1.

2. In panels C and D of Figures 2 and 3, it would be helpful to directly annotate the observed FDR values, or add reference lines/labels, so readers can more easily assess calibration.

3. The R package would benefit from a more complete tutorial, including a minimal working example, required input formats, parameter choices, interpretation of gene–factor and global-test outputs, and guidance on choosing the number of factors.

4. The gene labels at the bottom of Figure 5C are not visible.

**********

**Have all data underlying the figures and results presented in the manuscript been provided?**

Large-scale datasets should be made available via a public repository as described in the *PLOS Genetics*  data availability policy, and numerical data that underlies graphs or summary statistics should be provided in spreadsheet form as supporting information.

Reviewer #1: None

Reviewer #2: None

Reviewer #3: Yes

**********

PLOS authors have the option to publish the peer review history of their article (what does this mean?). If published, this will include your full peer review and any attached files.

If you choose “no”, your identity will remain anonymous but your review may still be made public.

**Do you want your identity to be public for this peer review?**  For information about this choice, including consent withdrawal, please see our Privacy Policy.

Reviewer #1: No

Reviewer #2: No

Reviewer #3: No

[NOTE: If reviewer comments were submitted as an attachment file, they will be attached to this email and accessible via the submission site. Please log into your account, locate the manuscript record, and check for the action link "View Attachments". If this link does not appear, there are no attachment files.]

**Figure resubmission:**

While revising your submission, we strongly recommend that you use PLOS’s NAAS tool (https://ngplosjournals.pagemajik.ai/artanalysis) to test your figure files. NAAS can convert your figure files to the TIFF file type and meet basic requirements (such as print size, resolution), or provide you with a report on issues that do not meet our requirements and that NAAS cannot fix.

After uploading your figures to PLOS’s NAAS tool - https://ngplosjournals.pagemajik.ai/artanalysis, NAAS will process the files provided and display the results in the "Uploaded Files" section of the page as the processing is complete. If the uploaded figures meet our requirements (or NAAS is able to fix the files to meet our requirements), the figure will be marked as "fixed" above. If NAAS is unable to fix the files, a red "failed" label will appear above. When NAAS has confirmed that the figure files meet our requirements, please download the file via the download option, and include these NAAS processed figure files when submitting your revised manuscript.

**Reproducibility:**

To enhance the reproducibility of your results, we recommend that authors of applicable studies deposit laboratory protocols in protocols.io, where a protocol can be assigned its own identifier (DOI) such that it can be cited independently in the future. Additionally, PLOS ONE offers an option to publish peer-reviewed clinical study protocols. Read more information on sharing protocols at https://plos.org/protocols?utm_medium=editorial-email&utm_source=authorletters&utm_campaign=protocols

# Author comment: Author response to Decision Letter 1

**DOI:** 10.1371/journal.pgen.1012126.r002

14 Jul 2026

Attachment Submitted filename: FM-GPT_ResponseLetter_vf.docx

# Peer review: Decision Letter 1

**DOI:** 10.1371/journal.pgen.1012126.r003

**Authors:** Lin S. Chen, Xiaofeng Zhu

3 Aug 2026

PGENETICS-D-26-00334R1

FM-GPT: Bayesian fine mapping for phenome-wide transcriptome-wide association studies

PLOS Genetics

Dear Dr. Ma,

Thank you for submitting your manuscript to PLOS Genetics. The three reviewers are largely satisfied with the revised manuscript, although a few minor points remain to be addressed. We invite you to submit a revised version that addresses these remaining comments.

Please submit your revised manuscript within by Sep 02 2026 11:59PM. If you will need more time than this to complete your revisions, please reply to this message or contact the journal office at plosgenetics@plos.org. When you're ready to submit your revision, log on to https://www.editorialmanager.com/pgenetics/ and select the 'Submissions Needing Revision' folder to locate your manuscript file.

Please include the following items when submitting your revised manuscript:

* A letter that responds to each point raised by the editor and reviewer(s). You should upload this letter as a separate file labeled 'Response to Reviewers'. This file does not need to include responses to formatting updates and technical items listed in the 'Journal Requirements' section below.

* A marked-up copy of your manuscript that highlights changes made to the original version. You should upload this as a separate file labeled 'Revised Manuscript with Track Changes'.

* An unmarked version of your revised paper without tracked changes. You should upload this as a separate file labeled 'Manuscript'.

If you would like to make changes to your financial disclosure, competing interests statement, or data availability statement, please make these updates within the submission form at the time of resubmission. Guidelines for resubmitting your figure files are available below the reviewer comments at the end of this letter.

As the corresponding author, your ORCID iD is verified in the submission system and will appear in the published article. PLOS supports the use of ORCID, and we encourage all coauthors to register for an ORCID iD and use it as well. Please encourage your coauthors to verify their ORCID iD within the submission system before final acceptance, as unverified ORCID iDs will not appear in the published article. Only the individual author can complete the verification step; PLOS staff cannot verify ORCID iDs on behalf of authors.

We look forward to receiving your revised manuscript.

Kind regards,

Lin S. Chen, Ph.D.

Academic Editor

PLOS Genetics

Xiaofeng Zhu

Section Editor

PLOS Genetics

Aimée Dudley

Editor-in-Chief

PLOS Genetics

Anne Goriely

Editor-in-Chief

PLOS Genetics

**Additional Editor Comments (if provided):**

**Journal Requirements:**

If the reviewer comments include a recommendation to cite specific previously published works, please review and evaluate these publications to determine whether they are relevant and should be cited. There is no requirement to cite these works unless the editor has indicated otherwise.

1) Please amend your detailed Financial Disclosure statement. This is published with the article. It must therefore be completed in full sentences and contain the exact wording you wish to be published.

1) State the initials, alongside each funding source, of each author to receive each grant. For example: "This work was supported by the National Institutes of Health (####### to AM; ###### to CJ) and the National Science Foundation (###### to AM).".

If you did not receive any funding for this study, please simply state: u201cThe authors received no specific funding for this work.u201d

**Reviewers' comments:**

Reviewer's Responses to Questions

**Comments to the Authors:**

**Please note here if the review is uploaded as an attachment.**

Reviewer #1: The authors have carefully addressed all my questions. Thanks!

Reviewer #2: The authors have addressed most of my comments. I recommend acceptance after minor revision. See details of my remaining comments below.

1. To address my previous comment 4, the authors proposed an omnibus gene-level BFDR. In the revised definition, the discovery indicator is written as I{PIP_j < z}, which appears inconsistent with selecting high PIP genes. Please double check.

2. The revised Figure 1B is clearer and now includes region-specific modeling, priors, and mixed-outcome augmentation. One minor issue in the legend: "Z^R and Xhat^G are the genotype estimated GReX data from GWAS". This sentence appears unclear and possibly includes notation errors. Please double check and revise.

3. For Figure 5E, the authors mentioned in the response that they plot the "dominant factor for each gene," but factor loadings are phenotype-by-factor quantities, not gene-specific unless combined with gene-factor effects. The definition "largest overall loading magnitude for that gene" is still ambiguous. I suggest explicitly defining whether the dominant factor is chosen by β_jl, the factor-specific PIP, the product of gene-factor effect and phenotype loading magnitude, only the norm of the factor-loading vector, or other ways. These choices have different interpretations. Please also consider clarifying in the legend that the heatmap is a visualization summary only and does not represent the full gene-phenotype association structure, since genes may act through multiple latent factors.

Reviewer #3: The authors have addressed my major concerns. There are some minor comments.

1. The reference to LDtect is wrong( ref 21). This should refer to PMID: 26395773

2. Typo in Section title: 'FM-GPT identifies genes that influence multiple medial conditions derived from EHR data' should read 'multiple medical conditions.'

3. In the last paragraph of Introduction, the phrase “In an brain-wide genetic analysis” should be changed to “In a brain-wide genetic analysis.”

**********

**Have all data underlying the figures and results presented in the manuscript been provided?**

Large-scale datasets should be made available via a public repository as described in the *PLOS Genetics*  data availability policy, and numerical data that underlies graphs or summary statistics should be provided in spreadsheet form as supporting information.

Reviewer #1: None

Reviewer #2: None

Reviewer #3: Yes

**********

PLOS authors have the option to publish the peer review history of their article (what does this mean?). If published, this will include your full peer review and any attached files.

If you choose “no”, your identity will remain anonymous but your review may still be made public.

**Do you want your identity to be public for this peer review?**  For information about this choice, including consent withdrawal, please see our Privacy Policy.

Reviewer #1: No

Reviewer #2: No

Reviewer #3: No

[NOTE: If reviewer comments were submitted as an attachment file, they will be attached to this email and accessible via the submission site. Please log into your account, locate the manuscript record, and check for the action link "View Attachments". If this link does not appear, there are no attachment files.]

**Figure resubmission:**

While revising your submission, we strongly recommend that you use PLOS’s NAAS tool (https://ngplosjournals.pagemajik.ai/artanalysis) to test your figure files. NAAS can convert your figure files to the TIFF file type and meet basic requirements (such as print size, resolution), or provide you with a report on issues that do not meet our requirements and that NAAS cannot fix.

After uploading your figures to PLOS’s NAAS tool - https://ngplosjournals.pagemajik.ai/artanalysis, NAAS will process the files provided and display the results in the "Uploaded Files" section of the page as the processing is complete. If the uploaded figures meet our requirements (or NAAS is able to fix the files to meet our requirements), the figure will be marked as "fixed" above. If NAAS is unable to fix the files, a red "failed" label will appear above. When NAAS has confirmed that the figure files meet our requirements, please download the file via the download option, and include these NAAS processed figure files when submitting your revised manuscript.

**Reproducibility:**

To enhance the reproducibility of your results, we recommend that authors deposit laboratory protocols in protocols.io, where a protocol can be assigned its own identifier (DOI) such that it can be cited independently in the future. Additionally, PLOS ONE offers an option to publish peer-reviewed clinical study protocols. Read more information on sharing protocols at https://plos.org/protocols?utm_medium=editorial-email&utm_source=authorletters&utm_campaign=protocols

# Author comment: Author response to Decision Letter 2

**DOI:** 10.1371/journal.pgen.1012126.r004

22 Aug 2026

Attachment Submitted filename: FM-GPT_ResponseLetter_pgenetics_R2.docx

# Editor report: Decision Letter 2

**DOI:** 10.1371/journal.pgen.1012126.r005

**Authors:** Lin S. Chen, Xiaofeng Zhu

24 Aug 2026

Dear Dr Ma,

We are pleased to inform you that your manuscript entitled "FM-GPT: Bayesian fine mapping for phenome-wide transcriptome-wide association studies" has been editorially accepted for publication in PLOS Genetics. Congratulations!

Before your submission can be formally accepted and sent to production you will need to complete our formatting changes, which you will receive in a follow up email. Please be aware that it may take several days for you to receive this email; during this time no action is required by you. Please note: the accept date on your published article will reflect the date of this provisional acceptance, but your manuscript will not be scheduled for publication until the required changes have been made.

Once your paper is formally accepted, an uncorrected proof of your manuscript will be published online ahead of the final version, unless you’ve already opted out via the online submission form. If, for any reason, you do not want an earlier version of your manuscript published online or are unsure if you have already indicated as such, please let the journal staff know immediately at plosgenetics@plos.org.

In the meantime, please log into Editorial Manager at https://www.editorialmanager.com/pgenetics/, click the "Update My Information" link at the top of the page, and update your user information to ensure an efficient production and billing process. Note that PLOS requires an ORCID iD for all corresponding authors. Therefore, please ensure that you have an ORCID iD and that it is validated in Editorial Manager. To do this, go to ‘Update my Information’ (in the upper left-hand corner of the main menu), and click on the Fetch/Validate link next to the ORCID field.  This will take you to the ORCID site and allow you to create a new iD or authenticate a pre-existing iD in Editorial Manager.

If you have a press-related query, or would like to know about making your underlying data available (as you will be aware, this is required for publication), please see the end of this email. If your institution or institutions have a press office, please notify them about your upcoming article at this point, to enable them to help maximise its impact. Inform journal staff as soon as possible if you are preparing a press release for your article and need a publication date.

Thank you again for supporting open-access publishing; we are looking forward to publishing your work in PLOS Genetics!

Yours sincerely,

Lin S. Chen, Ph.D.

Academic Editor

PLOS Genetics

Xiaofeng Zhu

Section Editor

PLOS Genetics

Aimée Dudley

Editor-in-Chief

PLOS Genetics

Anne Goriely

Editor-in-Chief

PLOS Genetics

www.plosgenetics.org

BlueSky: @plos.bsky.social

----------------------------------------------------

Comments from the reviewers (if applicable):

----------------------------------------------------

**Data Deposition**

If you have submitted a Research Article or Front Matter that has associated data that are not suitable for deposition in a subject-specific public repository (such as GenBank or ArrayExpress), one way to make that data available is to deposit it in the Dryad Digital Repository. As you may recall, we ask all authors to agree to make data available; this is one way to achieve that. A full list of recommended repositories can be found on our website.

The following link will take you to the Dryad record for your article, so you won't have to re‐enter its bibliographic information, and can upload your files directly:

http://datadryad.org/submit?journalID=pgenetics&manu=PGENETICS-D-26-00334R2

More information about depositing data in Dryad is available at http://www.datadryad.org/depositing. If you experience any difficulties in submitting your data, please contact help@datadryad.org for support.

Additionally, please be aware that our data availability policy requires that all numerical data underlying display items are included with the submission, and you will need to provide this before we can formally accept your manuscript, if not already present.

----------------------------------------------------

**Press Queries**

If you or your institution will be preparing press materials for this manuscript, or if you need to know your paper's publication date for media purposes, please inform the journal staff as soon as possible so that your submission can be scheduled accordingly. Your manuscript will remain under a strict press embargo until the publication date and time. This means an early version of your manuscript will not be published ahead of your final version. PLOS Genetics may also choose to issue a press release for your article. If there's anything the journal should know or you'd like more information, please get in touch via plosgenetics@plos.org.

# Editor report: Acceptance letter

**DOI:** 10.1371/journal.pgen.1012126.r006

**Authors:** Lin S. Chen, Xiaofeng Zhu

PGENETICS-D-26-00334R2

FM-GPT: Bayesian fine mapping for phenome-wide transcriptome-wide association studies

Dear Dr Ma,

We are pleased to inform you that your manuscript entitled "FM-GPT: Bayesian fine mapping for phenome-wide transcriptome-wide association studies" has been formally accepted for publication in PLOS Genetics! Your manuscript is now with our production department and you will be notified of the publication date in due course.

The corresponding author will soon be receiving a typeset proof for review, to ensure errors have not been introduced during production. Please review the PDF proof of your manuscript carefully, as this is the last chance to correct any errors. Please note that major changes, or those which affect the scientific understanding of the work, will likely cause delays to the publication date of your manuscript.

Soon after your final files are uploaded, unless you have opted out or your manuscript is a front-matter piece, the early version of your manuscript will be published online. The date of the early version will be your article's publication date. The final article will be published to the same URL, and all versions of the paper will be accessible to readers.

For Research Articles, you will receive an invoice from PLOS for your publication fee after your manuscript has reached the completed accept phase. If you receive an email requesting payment before acceptance or for any other service, this may be a phishing scheme. Learn how to identify phishing emails and protect your accounts at https://explore.plos.org/phishing.

Thank you again for supporting PLOS Genetics and open-access publishing. We are looking forward to publishing your work!

With kind regards,

Sharmila Kamatchi

PLOS Genetics

On behalf of:

The PLOS Genetics Team

Carlyle House, Carlyle Road, Cambridge CB4 3DN | United Kingdom

plosgenetics@plos.org | +44 (0) 1223-442823

plosgenetics.org | Twitter: @PLOSGenetics
